# Heterogeneity-aware Clustered Distributed Learning for Multi-source Data Analysis

**Published:** 2024

**Authors:** Yuanxing Chen, Qingzhao Zhang, Shuangge Ma, Kuangnan Fang

**Affiliations:** Department of Statistics and Data Science, School of Economics, Xiamen University, Xiamen, 361005, China; Department of Statistics and Data Science, School of Economics, The Wang Yanan Institute for Studies in Economics, Xiamen University, Xiamen, 361005, China; Department of Biostatistics, Yale University, New Haven, CT 06520, USA; Department of Statistics and Data Science, School of Economics, Xiamen University, Xiamen, 361005, China

**Keywords:** high dimensionality, data heterogeneity, clustering structure, sparsity, penalization

## Abstract

In diverse fields ranging from finance to omics, it is increasingly common that data is distributed with multiple individual sources (referred to as “clients” in some studies). Integrating raw data, although powerful, is often not feasible, for example, when there are considerations on privacy protection. Distributed learning techniques have been developed to integrate summary statistics as opposed to raw data. In many existing distributed learning studies, it is stringently assumed that all the clients have the same model. To accommodate data heterogeneity, some federated learning methods allow for client-specific models. In this article, we consider the scenario that clients form clusters, those in the same cluster have the same model, and different clusters have different models. Further considering the clustering structure can lead to a better understanding of the “interconnections” among clients and reduce the number of parameters. To this end, we develop a novel penalization approach. Specifically, group penalization is imposed for regularized estimation and selection of important variables, and fusion penalization is imposed to automatically cluster clients. An effective ADMM algorithm is developed, and the estimation, selection, and clustering consistency properties are established under mild conditions. Simulation and data analysis further demonstrate the practical utility and superiority of the proposed approach.

## 1. Introduction

In diverse fields, it is increasingly common that data is distributed with multiple individual sources (referred to as “clients” in this article and some published studies). For example, in financial studies, it is common that data is with, for example, multiple individual bank branches. In omics studies, it is common that multiple independent studies have generated their own data and address the same scientific question. The power of integrating data from multiple sources has been well identified (Liu et al., 2014). A family of studies/methods integrate raw data (Tang and Song, 2016; Huang et al., 2017). Such integrative analysis methods, although effective, are not always feasible due to the need for privacy protection.

Since the huge financial loss of Facebook due to its privacy breach (Kelleher, 2018), privacy issues have once again attracted widespread attention from both the industry and academia. The innovations in a wide range of industries, such as smart healthcare, financial technology, and surveillance systems, rely on newly developing machine learning methods, and then, the development of machine learning methods needs to take privacy protection into full consideration (Liu et al., 2021). In machine learning, privacy protection can be roughly divided into three mechanisms, including homomorphic encryption, obfuscation, and aggregation (Liu et al., 2021). Homomorphic encryption facilitates the processing of the encrypted data without the need to access the raw data. Such technique has been successfully applied to regression (Chen et al., 2018), classification (Bost et al., 2015), and deep neural networks (Gilad-Bachrach et al., 2016). Obfuscation mechanism can be achieved by adding noises to the model parameters or the original data set, and differential privacy (DP) (Dwork, 2006; Agarwal et al., 2018) is the most popular scheme in obfuscation. Aggregation organizes multiple parties to join a machine learning task while avoiding the transmission of the raw data (Zhang et al., 2021).

Distributed learning (DL), as the most famous framework in aggregation, aims to train the global model by aggregating the summary statistics from all clients without sharing the raw data. The existing works can be divided into two categories based on whether the number of iterations is once or multiple times (Zhou et al., 2024). One-shot approaches require just one communication round between the local clients and the central server, in which the divide-and-conquer (DC) strategy is the most popular one designed to reduce communication burden and improve feasibility and performance in the analysis of big data (Lee et al., 2017; Battey et al., 2018). Although one-shot approaches have the lowest communication costs, to obtain the same convergence rate with a centralized estimator, a sufficient sample size of each client relative to the number of clients is necessary (Wang et al., 2017). To further relax this constraint, communication-iterative approaches, such as distributed approximate Newton-type method (Shamir et al., 2014) and communication-efficient surrogate likelihood (Jordan et al., 2019), have been developed. Additionally, when data is large, sending raw data from individual clients to a central server and constructing a statistical model with the pooled data may lead to considerable computational cost (Bhowmick et al., 2018). However, the transmission and aggregation of summary statistics in DL can alleviate communication and computation burden simultaneously.

The DL methods described above all assume that the individual clients share the same data generation model. This assumption, although convenient, may be overly stringent. In raw data-based integrative analysis (Tang and Song, 2016; Huang et al., 2017), it has been well established that data may be heterogeneous and demand different models. The issue of data heterogeneity, known as non-i.i.d data, in the distributed learning setting has attracted widespread attention. For example, different hospitals usually store electronic health records (EHR) in their local sites and are unwilling to share their raw data with others. The data heterogeneity (reflected by heterogeneous outcome-covariate relationships) due to different patient populations should be further considered (Liu et al., 2022; Duan et al., 2022). Besides, Yu et al. (2020) further confirmed that incorrectly borrowing information from other sites with large heterogeneity leads to unreliable inferences and/or low prediction power. As the data heterogeneity can be caused by differences in sample characteristics, data collection techniques, and multiple other factors (Ghosh et al., 2020), the personalized DL methods that borrow strength from similar individual clients have attracted growing interest (Smith et al., 2017).

Federated learning (FL), as a popular DL paradigm, pays more attention to the problem of data islands in a collaborative manner compared to general distributed learning (Kaissis et al., 2020; Liu et al., 2021). It provides a novel method to build personalized models without violating user privacy (Zhang et al., 2021). It typically involves multiple rounds of communication between the central server and local clients to obtain the final model estimates. Clustered federated learning (CFL), as a special case of FL, aims to classify clients into multiple clusters such that clients in the same cluster share the same model, and different clusters have different models (Ghosh et al., 2020; Marfoq et al., 2021). This type of heterogeneity analysis may have been more popular in computer science than statistics and is crucial in applications such as recommendation systems and personalized advertisement placement (Ghosh et al., 2020). Intuitively, assuming and identifying a clustering structure can lead to a better understanding of the “interconnections” among clients (those in the same cluster are more alike and can be more closely related to each other) and a smaller number of model parameters. For instance, mobile phone users (clients) may focus on different clusters of news, like politics, sports, or fashion. Besides, different groups of customers are interested in different categories of ads. Thus, having a deeper understanding of the “interconnections” within a cluster can benefit more accurate personalized recommendations.

A common limitation shared by the existing CFL methods is that it is usually challenging to determine the number of clusters. For example, Ghosh et al. (2020) and Marfoq et al. (2021) first pre-specified the number of clusters and then alternately updated the cluster membership of each client and model parameters for each cluster. Similar to classic clustering analysis, results can be sensitive to the number of clusters, and in practice, usually, there is not enough information to accurately specify this number. Specifically, Ghosh et al. (2020) mentioned in one experiment that by setting a larger number of clusters, their algorithm can identify the correct number by emptying the excess clusters. However, in most cases (refer to the numerical results in Section 4), the ultimately estimated number of clusters remains unchanged, staying at the initially pre-specified number. In addition, since the membership updating algorithm in Ghosh et al. (2020) is similar to K-means, the final clustering structure is highly sensitive to the initial clustering segmentation, which Chen, Zhang, Ma, and Fang further leads to unstable estimation results (refer to the numerical results in Section 4). Moreover, both Ghosh et al. (2020) and Marfoq et al. (2021) focused on the dense setting with all parameters being nonzero. Therefore, to deal with high-dimensional scenarios with sparsity settings, we should develop a new clustered distributed learning method, which can generate stable and sparse estimates (for interpretation) and identify the true number of clusters in a data-driven way. Of course, this method should also accommodate data heterogeneity and privacy protection at the same time.

In the statistical literature, there are also a few heterogeneous distributed learning methods that allow for client-specific models. For example, Zhao et al. (2016) proposed a heterogeneous distributed learning method with a partially linear model, under which the nonparametric parameter is assumed to be shared by all clients, while the parametric parameters are allowed to be client-specific. Duan et al. (2022) extended the surrogate likelihood function approach to allow client-specific nuisance parameters by adopting a surrogate estimating equation technique. It is noted that these two (and some other) studies are limited to low-dimensional settings. Cai et al. (2022) further studied the high-dimensional heterogeneous setting by aggregating local summary statistics under a generalized linear model. As recognized in Tang et al. (2021), allowing all clients to have individual models may lead to a large number of redundant parameters, negatively affecting estimation and inference.

In this article, we consider the integrative analysis of multi-source data under privacy protection. We utilize the summary statistics instead of the raw individual-level data to avoid privacy breaches while learning parametric models based on the distributed learning framework. Here the summary statistics can contain initial parameter estimates, gradient vectors, hessian matrices, and so on. To sufficiently accommodate data heterogeneity, captured by different model parameters, clients are allowed to have different models. Specifically, motivated by the success of clustered federated learning, we consider the scenario where clients form clusters, and the models are cluster-specific. Besides, to address the high-dimensional issues with sparsity assumption, we focus on the scenario where those models have the same sparsity structure (set of important variables) and note that the proposed strategy can be extended to accommodate different sparsity structures.

To achieve the goal of simultaneous estimation, variable selection, and clustering, we develop an integrative clustered regression (ICR) method, which may advance from the existing literature in multiple important ways. First, compared to methods that assume homogeneity (Lee et al., 2017), it is more flexible and can effectively accommodate data heterogeneity. Second, compared to methods that allow for client-specific models (Zhao et al., 2016; Duan et al., 2022), it can lead to a better understanding of the similarity/differences among data sets and a smaller number of parameters (and hence improved estimation). Third, compared to the existing CFL methods (Ghosh et al., 2020; Marfoq et al., 2021), it can data-dependently and conveniently determine the number of clusters, and the estimated cluster memberships are not sensitive to the initial cluster partition, with the assistance of penalized fusion. Fourth, compared to the dense setting applied to neutral network (Ghosh et al., 2020), the sparse parameter estimates due to sparsity penalty can facilitate model interpretability as well as efficient inference and training in neutral network (Hoefler et al., 2021). Last but not least, it can accommodate multiple types of data/models, and our computational and theoretical developments can shed broader insights.

The rest of the article is organized as follows. In Section 2, we introduce the data/model settings, the proposed approach, and an effective proximal ADMM algorithm. In Section 3, we rigorously establish that the proposed approach enjoys the estimation, variable selection, and clustering consistency properties. Numerical studies, including simulation in Section 4 and data analysis in Section 5, demonstrate the practical utilization and superiority of the proposed approach. Brief discussions are provided in Section 6. The proofs of theoretical results and additional numerical results are relegated to the Appendix.

## 2. Methods

In this section, we first introduce the integrative clustered model in a distributed setup and then develop a proximal ADMM algorithm to obtain the ICR estimator.

### 2.1 Integrative Analysis under Privacy Constraints

Suppose that there are K independent clients, and for the kth client, there are $n_{k}$ observations. The total sample size is $N=\sum_{k=1}^{K}n_{k}$. For the kth client, let $y_{i}^{\left(k\right)}$ and $x_{i}^{\left(k\right)}={\left(x_{i1}^{\left(k\right)},\ldots ,x_{ip}^{\left(k\right)}\right)}^{⊤}\in R^{p}$ be the response and covariate vector of the ith observation, respectively, where the first element of $x_{i}^{\left(k\right)}$ is fixed as $x_{i1}^{\left(k\right)}\equiv 1$ to accommodate intercept. Accordingly, let $X^{\left(k\right)}={\left(x_{1}^{\left(k\right)},\ldots ,x_{n_{k}}^{\left(k\right)}\right)}^{⊤}$ and $Y^{\left(k\right)}={\left(y_{1}^{\left(k\right)},\ldots ,y_{n_{k}}^{\left(k\right)}\right)}^{⊤}$ denote the design matrix and response vector of the kth client, respectively. Let f(⋅) be the pre-specified twice-differentiable loss function, and define the true population coefficients as

$$\theta^{*(k)}=\underset{\theta^{\left(k\right)}\in R^{p}}{\text{arg}\;\text{min}}\;{ℒ}_{k}\left(\theta^{\left(k\right)}\right)\;\text{and}\;{ℒ}_{k}\left(\theta^{\left(k\right)}\right)=E\left[f\left(\theta^{(k)⊤}x_{i}^{\left(k\right)},y_{i}^{\left(k\right)}\right)\right],\;k\in \left[K\right],$$

where $\theta^{\left(k\right)}={\left(\theta_{1}^{\left(k\right)},\ldots ,\theta_{p}^{\left(k\right)}\right)}^{⊤}$ is the p-dimensional coefficient vector, and [d] denotes the index set {1,…,d} for an integer d. Accordingly, the empirical local and global loss functions are defined as

$${\hat{ℒ}}_{k}\left(\theta^{\left(k\right)}\right)=\frac{1}{n_{k}}\sum_{i=1}^{n_{k}}f\left(x_{i}^{(k)⊤}\theta^{\left(k\right)},y_{i}^{\left(k\right)}\right),\;k\in [K]\;\text{and}\;\hat{ℒ}(\theta )=\frac{1}{N}\sum_{k=1}^{K}n_{k}{\hat{ℒ}}_{k}\left(\theta^{\left(k\right)}\right),$$

respectively, where $\theta =\left(\theta^{\left(1\right)},\cdots ,\theta^{\left(K\right)}\right)$ is a p×K coefficient matrix with the jth row $\theta_{j}={\left(\theta_{j}^{\left(1\right)},\cdots ,\theta_{j}^{\left(K\right)}\right)}^{⊤}$. Assume that $𝒢=\left\{{𝒢}^{\left(1\right)},\ldots ,{𝒢}^{\left(M\right)}\right\}$ forms a non-overlapping partition of {1,…,K}, and that clients from the same cluster share the same coefficient vector. That is, given m∈[M], for any $k\in {𝒢}^{\left(m\right)},\theta^{*(k)}=\psi^{*(m)}$, where $\psi^{*(m)}$ is the cluster-specific coefficient vector for cluster m. Additionally, for each covariate, its coefficients across the K clients can be viewed as a group (Cai et al., 2022), leading to p groups corresponding to the covariates.

For simultaneous regularized estimation, variable selection, and identification of the clustering structure of clients, we propose the objective function with the ideal pooling (IP) strategy

(1) $${\hat{𝒬}}_{\text{IP}}(\theta )=\hat{ℒ}(\theta )+{𝒫}_{\lambda_{1}}(\theta )+{𝒫}_{\lambda_{2}}(\theta )\;=\frac{1}{N}\sum_{k=1}^{K}n_{k}{\hat{ℒ}}_{k}\left(\theta^{\left(k\right)}\right)+\sum_{j=2}^{p}p_{\tau }\left({\left\|\theta_{j}\right\|}_{2},\lambda_{1}\right)+\sum_{k<k^{\prime }}p_{\tau }\left({\left\|\theta^{\left(k\right)}-\theta^{\left(k^{\prime }\right)}\right\|}_{2},\lambda_{2}\right),$$

where penalty ${𝒫}_{\lambda_{1}}(\theta )$ is mainly for regularized estimation and variable selection, and penalty ${𝒫}_{\lambda_{2}}(\theta )$ is mainly for clustering. Here $p_{\tau }(,)$ is a penalty function with concavity parameter $\tau ,\|\cdot {\|}_{2}$ is the $L_{2}$ norm, and $\lambda_{1},\lambda_{2}$ are two non-negative tuning parameters.

**Figure 1:**
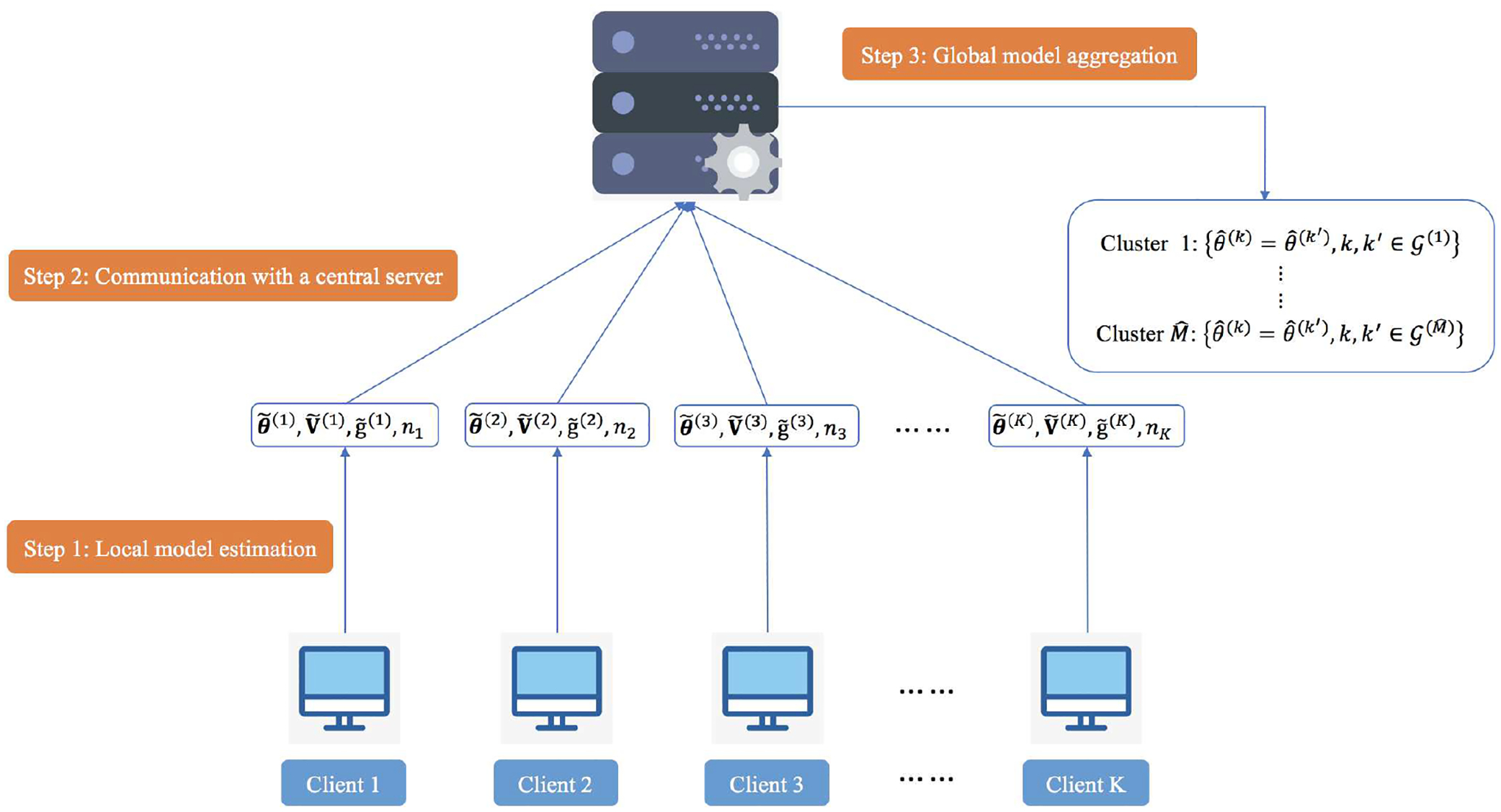
Scheme of the proposed analysis.

With the privacy-preservation constraints, raw data of the individual client is not available, and hence objective function ${\hat{𝒬}}_{\text{IP}}(\theta )$ in (1) cannot be directly implemented. To tackle this problem, we adopt the least-square approximation (LSA) of He et al. (2016) and Zhu et al. (2021), which leads to the objective function

(2) $${\hat{𝒬}}_{1}(\theta )=\frac{1}{N}\sum_{k=1}^{K}n_{k}{\left(\theta^{\left(k\right)}-{\tilde{\theta }}^{\left(k\right)}\right)}^{⊤}{\tilde{V}}^{\left(k\right)}\left(\theta^{\left(k\right)}-{\tilde{\theta }}^{\left(k\right)}\right)+{𝒫}_{\lambda_{1}}(\theta )+{𝒫}_{\lambda_{2}}(\theta ),$$

where ${\tilde{\theta }}^{\left(k\right)}$ is the local estimator of the kth client, and ${\tilde{V}}^{\left(k\right)}=\partial^{2}{\hat{ℒ}}_{k}\left({\tilde{\theta }}^{\left(k\right)}\right)/\partial \theta^{\left(k\right)}\partial \theta^{(k)⊤}$ is the Hessian matrix of ${\hat{ℒ}}_{k}\left(\theta^{\left(k\right)}\right)$ with respect to $\theta^{\left(k\right)}$ at ${\tilde{\theta }}^{\left(k\right)}$. He et al. (2016) recommended adopting ordinary least square (OLS) estimates as the local estimators when $p<n_{k}$. Under high-dimensional settings, OLS estimates are not available, and a “straightforward” approach is to replace the OLS estimates with the Lasso estimates. However, the computationally efficient ordinary Lasso estimates are usually biased, and the debiased Lasso estimates (van de Geer et al., 2014) are often computationally expensive. Inspired by Cai et al. (2022), we propose the ICR estimator $\hat{\theta }$ by minimizing the following objective function

(3) $${\hat{𝒬}}_{\text{ICR}}\left(\theta \right)=\frac{1}{N}\sum_{k=1}^{K}n_{k}\left(\theta^{\left(k\right)⊤}{\tilde{V}}^{\left(k\right)}\theta^{\left(k\right)}-2\theta^{\left(k\right)⊤}{\tilde{\zeta }}^{\left(k\right)}\right)+{𝒫}_{\lambda_{1}}\left(\theta \right)+{𝒫}_{\lambda_{2}}\left(\theta \right),$$

where ${\tilde{\zeta }}^{\left(k\right)}={\tilde{V}}^{\left(k\right)}{\tilde{\theta }}^{\left(k\right)}-{\tilde{g}}^{\left(k\right)}$ and ${\tilde{g}}^{\left(k\right)}=\partial {\hat{ℒ}}_{k}\left({\tilde{\theta }}^{\left(k\right)}\right)/\partial \theta^{\left(k\right)}$ is the gradient of ${\hat{ℒ}}_{k}\left(\theta^{\left(k\right)}\right)$ with respect to $\theta^{\left(k\right)}$ at ${\tilde{\theta }}^{\left(k\right)}$. Here we use Lasso estimators as local estimators ${\tilde{\theta }}^{\left(k\right)}$. For the penalty function, viable choices include SCAD (Fan and Li, 2001), MCP (Zhang, 2010), and others. We adopt MCP in our numerical studies. Note that, with the first loss term in (3), we can achieve debiasing without actually resorting to the computationally expensive debiased estimates (we refer to Cai et al., 2022 for more details). The overall analysis approach is schematically presented in Figure 1. It consists of generating individual estimates based on raw data by individual clients, sending summary estimates from local clients to a central server, conducting the proposed estimation, and outputting the final estimators to guide downstream analysis/actions.

This approach has been motivated by the following considerations. In ${\hat{𝒬}}_{\text{ICR}}(\theta )$, we only make use of four summary statistics, namely the initial local estimators ${\left\{{\tilde{\theta }}^{\left(k\right)}\right\}}_{k=1}^{K}$, corresponding gradient vectors ${\left\{{\tilde{g}}^{\left(k\right)}\right\}}_{k=1}^{K}$, Hessian matrices ${\left\{{\tilde{V}}^{\left(k\right)}\right\}}_{k=1}^{K}$, and local sample sizes ${\left\{n_{k}\right\}}_{k=1}^{K}$. That is, the proposed approach and estimate are fully based on the summary statistics as opposed to the raw data – data privacy protection is thus achieved. In (3), the first term measures lack-of-fit, and similar forms have been considered in the literature (Zhu et al., 2021; Cai et al., 2022). When higher-order estimation properties are not of interest, the estimates and Hessian matrices from the local clients contain sufficient information. The first penalty determines which covariates have overall nonzero effects, under the assumptions that one covariate may have different effects/coefficients for different clients, but the effects are either all nonzero or all zero. It is possible to replace it with more complex penalties, for example, those that can conduct two-level selection (Huang et al., 2017), to obtain “more subtle” information. The second is a fusion penalty (Ma and Huang, 2017), with which some clients may have exactly equal estimates. Clients k and $k^{\prime }$ are clustered together if and only if their estimates are equal. For identifying clustering structures, fusion penalization has been recognized to have multiple unique advantages and has been popular in the recent literature (Ma and Huang, 2017; Yang et al., 2019; Chen et al., 2021). For example, it translates clustering to an “easier” estimation problem and can more conveniently determine the number/structure of clusters (by examining the estimates). It is worth noting that most of the existing studies, such as Ma and Huang (2017) and Chen et al. (2021), focus on heterogeneity analysis with a single data set, while here we study the subgrouping structure of multiple clients (data sets). The identification of subgrouping can facilitate “personalized” analysis and improve individual analysis by reducing the number of parameters/increasing sample size. Additionally, different from Yang et al. (2019) and others that demand raw data, we can achieve the goal of privacy protection and reduce computational cost by analyzing summary statistics.

**Remark 1** *We claim that taking into account the clustering structure can reduce the number of parameters and we are going to further explain this from two perspectives. From the theoretical perspective, after assuming a latent cluster partition within* K *clients and the clients from the same cluster share the same parameters, the true number of model parameters depends on the number of clusters. To see this, note that if we allow client-specific parameters for all* K *clients, there are a total of* Kp *parameters that need to be estimated. However, the existing clustering structure leads to M cluster-specific parameters, which results in Mp parameters being estimated. Since* M *is usually much smaller than* K, *the proposed ICR method can utilize samples from all clients belonging to a cluster to estimate cluster-specific parameters, thus improving the theoretical convergence rate of estimation errors* (*see* Section 3 *for more details*). *From the computational perspective, although there are still Kp parameters involved in the estimation process, through penalized fusion, parameters belonging to the same cluster tend to be the same. As a result, the number of distinct parameters is greatly reduced. Based on this, for subsequent observations generated by clients from cluster* m, *predictions can be made based on the estimated* m*th cluster-specific parameters*.

**Remark 2** *It is worth emphasizing that the local estimators* ${\left\{{\tilde{\theta }}^{\left(k\right)}\right\}}_{k=1}^{K}$, *obtained by solving the corresponding local penalized loss functions, serve only as a part of summary statistics for obtaining the final ICR estimators ${\left\{{\hat{\theta }}^{\left(k\right)}\right\}}_{k=1}^{K}$*. *For the local estimators, the sparsity structures vary across different clients and there is no clustering structure among clients. On the contrary, the proposed ICR estimators share the same sparsity structure and clients from the same cluster share the same estimated parameters. This difference leads to better variable selection performance and higher estimation accuracy for the ICR estimators compared to the local estimators* (*see the numerical results in* Section 4).

### 2.2 Computational Algorithm

We use local linear approximation — LLA (Zou and Li, 2008) to approximate the fused penalty and propose an iterative algorithm. Specifically, in the tth iteration, we update the coefficients by solving

$$\underset{\theta \in R^{p\times K}}{\text{arg}\;\text{min}}\;\frac{1}{N}\sum_{k=1}^{K}n_{k}\left(\theta^{(k)⊤}{\tilde{V}}^{\left(k\right)}\theta^{\left(k\right)}-2\theta^{(k)⊤}{\tilde{\zeta }}^{\left(k\right)}\right)+\sum_{k<k^{\prime }}\omega_{kk^{\prime }}^{t-1}{\left\|\theta^{\left(k\right)}-\theta^{\left(k^{\prime }\right)}\right\|}_{2}+\sum_{j=2}^{p}p_{\tau }\left({\left\|\theta_{j}\right\|}_{2},\lambda_{1}\right),$$

where $\omega_{kk^{\prime }}^{t-1}=p_{\tau }^{\prime }\left({\left\|\theta^{(k),t-1}-\theta^{\left(k^{\prime }\right),t-1}\right\|}_{2},\lambda_{2}\right)$ denotes the weight and $p_{\tau }^{\prime }(x,\lambda )$ is the derivative of $p_{\tau }(x,\lambda )$ with respect to x. The above minimization problem can be reformulated as a constrained minimization problem

$$\underset{\theta \in R^{p\times K}}{\text{arg}\;\text{min}}\;\{\ell (\theta )≔\overset{g(\theta )}{\overbrace{\frac{1}{N}\sum_{k=1}^{K}n_{k}\left(\theta^{(k)⊤}{\tilde{V}}^{\left(k\right)}\theta^{\left(k\right)}-2\theta^{(k)⊤}{\tilde{\zeta }}^{\left(k\right)}\right)}}+\underset{h_{1}(\alpha )}{\underbrace{\sum_{k<k^{\prime }}\omega_{kk^{\prime }}^{t-1}{\left\|\alpha_{kk^{\prime }}\right\|}_{2}}}+\underset{h_{2}(\theta )}{\underbrace{\sum_{j=2}^{p}p_{\tau }\left({\left\|\theta_{j}\right\|}_{2},\lambda_{1}\right)}}\},\;\text{subject}\;\text{to}\;\theta^{\left(k\right)}-\theta^{\left(k^{\prime }\right)}=\alpha_{kk^{\prime }},\;1\leq k<k^{\prime }\leq K,$$

where $\alpha =\left(\alpha_{12},\ldots ,\alpha_{(K-1)K}\right)$ is a p×K(K−1)/2 matrix composed of the auxiliary variables. This optimization problem is equivalent to the minimization of the augmented Lagrangian

(4) $$\ell_{\nu }(\theta ,\alpha ,\xi )=\ell (\theta )+\sum_{k<k^{\prime }}\xi_{kk^{\prime }}^{⊤}\left(\theta^{\left(k\right)}-\theta^{\left(k^{\prime }\right)}-\alpha_{kk^{\prime }}\right)+\frac{\nu }{2}\sum_{k<k^{\prime }}{\left\|\theta^{\left(k\right)}-\theta^{\left(k^{\prime }\right)}-\alpha_{kk^{\prime }}\right\|}_{2}^{2},$$

where $\xi =\left(\xi_{12},\ldots ,\xi_{(K-1)K}\right)$ is a p×K(K−1)/2 matrix composed of the dual variables. ν is a small positive constant. Following Shimmura and Suzuki (2022), we can minimize objective function $\ell_{\nu }(\theta ,\alpha ,\xi )$ in (4) via the following iterations

(5) $$\left(\theta^{t},\alpha^{t}\right)=\underset{\theta ,\alpha }{\text{arg}\;\text{min}}\;\ell_{\nu }\left(\theta ,\alpha ,\xi^{t-1}\right),\;\xi_{kk^{\prime }}^{t}=\xi_{kk^{\prime }}^{t-1}+\nu \left(\theta^{(k),t}-\theta^{\left(k^{\prime }\right),t}-\alpha_{kk^{\prime }}^{t}\right),\;1\leq k<k^{\prime }\leq K.$$

To update (θ,α) via (4), we minimize η(θ) defined by

(6) $$\eta (\theta )≔\underset{\alpha }{\text{min}}\;\ell_{\nu }\left(\theta ,\alpha ,\xi^{t-1}\right)\;=\underset{\alpha }{\text{min}}\;\{\overset{\eta_{1}(\theta ,\alpha )}{\overbrace{\sum_{k<k^{\prime }}\left[\omega_{kk^{\prime }}^{t-1}{\left\|\alpha_{kk^{\prime }}\right\|}_{2}+\xi_{kk^{\prime }}^{t-1^{⊤}}\left(\theta^{\left(k\right)}-\theta^{\left(k^{\prime }\right)}-\alpha_{kk^{\prime }}\right)+\frac{\nu }{2}{\left\|\theta^{\left(k\right)}-\theta^{\left(k^{\prime }\right)}-\alpha_{kk^{\prime }}\right\|}_{2}^{2}\right]}}\}+g(\theta )+h_{2}(\theta )\;=\eta_{2}\left(\theta \right)+h_{2}\left(\theta \right).$$

Following Chi and Lange (2015), we define the proximal map with respect to Ω(v) as

$${\text{prox}}_{\sigma \Omega }\left(u\right)=\underset{v}{\text{arg}\;\text{min}}\left[\sigma \Omega (v)+\frac{1}{2}\|u-v{\|}_{2}^{2}\right].$$

Besides, the conjugate function of Ω(v) is defined by $\Omega^{*}(u)={\text{sup}}_{v}\left[u^{⊤}v-\Omega (v)\right]$. Then, it is easy to show that $\eta_{1}(\theta ,\alpha )$ is minimized when

(7) $$\alpha \left(\theta \right)={\text{prox}}_{\nu^{-1}h_{1}}\left(\theta A+\nu^{-1}\xi^{t-1}\right),$$

where $A=\left(e_{12},\ldots ,e_{(K-1)K}\right)$ is a K×K(K−1)/2 matrix and $e_{kk^{\prime }}=e_{k}-e_{k^{\prime }}$, in which $e_{k}$ is a K×1 vector whose kth element is 1 and the remaining elements are 0. Plugging (7) into $\eta_{1}(\theta ,\alpha )$ in (6) and combining the results of Theorem 1 and Lemmas 1—2 of Shimmura and Suzuki (2022), we can show that $\eta_{2}(\theta )$ is differentiable, and the gradient of $\eta_{2}(\theta )$ is

$$\frac{\partial \eta_{2}(\theta )}{\partial \theta }=\theta_{g}+\left[{\text{prox}}_{\nu h_{1}^{*}}\left(\nu \theta A+\xi^{t-1}\right)\right]A^{⊤},$$

where $\theta_{g}=2/N\left[n_{1}\left({\tilde{V}}^{\left(1\right)}\theta^{\left(1\right)}-{\tilde{\zeta }}^{\left(1\right)}\right),\ldots ,n_{K}\left({\tilde{V}}^{\left(K\right)}\theta^{\left(K\right)}-{\tilde{\zeta }}^{\left(K\right)}\right)\right]$ is a p×K matrix and $h_{1}^{*}$ is the conjugate function of $h_{1}$. Then, we can adopt one proximal gradient technique, called Fast Iterative Shrinkage-Thresholding Algorithm (FISTA) (Parikh et al., 2014), to obtain the solution of the first minimization problem in (5). Further, similar to equations (26) and (27) in Shimmura and Suzuki (2022), the second iterative step in (5) can be reformulated as

$$\xi^{t}=\left[{\text{prox}}_{\nu h_{1}^{*}}\left(\nu \theta^{t}A+\xi^{t-1}\right)\right].$$

The proposed proximal ADMM algorithm is summarized as follows.

Step 1. Obtain the initial estimates with $\left(\theta^{0},\xi^{0}\right)$.
Step 2. At iteration t,t=1,2,…, update $\theta^{t}$ as follows.
  Step 2.1. Initialize $u^{t-1,0}=\theta^{t-1,0}=\theta^{t-1}$ and $\rho_{0}=1$.
  Step 2.2. At iteration s,s=1,2,…, compute
$$\omega_{kk^{\prime }}^{t-1,s}\leftarrow p_{\tau }^{\prime }\left({\left\|u^{(k),t-1,s-1}-u^{\left(k^{\prime }\right),t-1,s-1}\right\|}_{2},\lambda_{2}\right),\;1\leq k<k^{\prime }\leq K,$$
$$\theta^{t-1,s}\leftarrow {\text{prox}}_{\varsigma h_{2}}\left[u^{t-1,s-1}-\varsigma \frac{\partial \eta_{2}\left(u^{t-1,s-1}\right)}{\partial \theta }\right],$$
$$\rho_{s}\leftarrow \frac{1+\sqrt{1+4\rho_{s-1}^{2}}}{2},\;u^{t-1,s}\leftarrow \theta^{t-1,s}+\frac{\rho_{s-1}-1}{\rho_{s}}\left(\theta^{t-1,s}-\theta^{t-1,s-1}\right).$$
  Step 2.3. Repeat Step 2.2 until convergence, and set $\theta^{t}\leftarrow \theta^{t-1,s}$.
Step 3. For $1\leq k<k^{\prime }\leq K$, update $\omega_{kk^{\prime }}^{t}\leftarrow p_{\tau }^{\prime }\left({\left\|\theta^{(k),t}-\theta^{\left(k^{\prime }\right),t}\right\|}_{2},\lambda_{2}\right)$.
Step 4. Update $\xi^{t}\leftarrow {\text{prox}}_{\nu h_{1}^{*}}\left(\nu \theta^{t}A+\xi^{t-1}\right)$.
Step 5. Repeat Steps 2—4 until convergence, and set $\alpha^{t}\leftarrow {\text{prox}}_{\nu^{-1}h_{1}}\left(\theta^{t}A+\nu^{-1}\xi^{t}\right)$.

In the above calculation, we conclude convergence if the absolute difference of estimates from two consecutive iterations is smaller than a predefined cutoff.

Remark 3 *There exist closed-form solutions for the proximal maps of* $\nu h_{1}^{*},\nu^{-1}h_{1}$, *and* $\varsigma h_{2}$. *Specifically, the proximal map of* $\nu h_{1}^{*}$ *is a projection function. And the proximal maps of* $\nu^{-1}h_{1}$ *and* $\varsigma h_{2}$ *can be easily derived as in* Ma and Huang (2017) *with* τ>ς. *In Step 2.2,* ς *denotes the step size. As in* Shimmura and Suzuki (2022), *we can derive the Lipschitz constant of* $\eta_{2}(\theta )$, *denoted by* $L_{\eta }=1+2\nu \;{\text{max}}_{k\in [K]}{\left(AA^{⊤}\right)}_{k,k}$, *and then set* $\varsigma =L_{\eta }^{-1}$. *With* ν=1 *and* τ=3,τ>ς *since* ς≤1/3. *Here, although superlinear convergence may be achieved if* ν→∞ (Rockafellar, 1976), *it is difficult to prove the convergence of ADMM when* ν *varies by iteration* (*see* Boyd et al., 2011, *Section 3.4.1*). *Therefore, while there may be improvements in convergence rate, varying* ν *may also lead to the algorithm failing to converge. In practice*, ν=1 *is widely adopted and achieves good convergence in the implementation of the ADMM algorithm for extensive studies* (Ma and Huang, 2017; Zhu and Qu, 2018; Ren et al., 2023).

**Remark 4** *The basic framework of our algorithm is ADMM* (Boyd et al., 2011), *of which one variant accommodates a differential loss function plus nonconvex penalties, and its convergence properties have been studied in* Ma and Huang (2017) *and* Tang et al. (2021). *The “standard” method for minimizing the first problem in* (5) *involves inverting a matrix* (Ma and Huang, 2017; Zhu and Qu, 2018), *which can be computationally difficult if* p *and* K *are large. A novelty in our algorithm is to replace this with the proximal gradient method, and convergence of the FISTA technique has been studied in* Beck and Teboulle (2009). *Therefore, by combining the convergence properties of* Beck and Teboulle (2009) *and* Tang et al. (2021), *the proposed algorithm is also expected to have satisfactory convergence*.

#### Tuning parameter selection

Following the literature, we set ν=1 and the concavity related parameter τ=3. Following Yang et al. (2019), we select $\lambda_{1}$ and $\lambda_{2}$ by minimizing the modified BIC defined as

$$\text{mBIC}\left(\lambda_{1},\lambda_{2}\right)=\frac{1}{N}\sum_{k=1}^{K}n_{k}\left({\left[{\hat{\theta }}^{\left(k\right)}\left(\lambda_{1},\lambda_{2}\right)\right]}^{⊤}{\tilde{V}}^{\left(k\right)}\left[{\hat{\theta }}^{\left(k\right)}\left(\lambda_{1},\lambda_{2}\right)\right]-2{\left[{\hat{\theta }}^{\left(k\right)}\left(\lambda_{1},\lambda_{2}\right)\right]}^{⊤}{\tilde{\zeta }}^{\left(k\right)}\right)+C_{N}\frac{\text{log}\;N}{N}\hat{q}\left(\lambda_{1},\lambda_{2}\right),$$

where $\hat{q}\left(\lambda_{1},\lambda_{2}\right)$ is the number of nonzero distinct coefficient vectors, and $C_{N}$ is a positive constant depending on N. Following Ma and Huang (2017), we adopt $C_{N}=\text{log}(\text{log}(Kp))$, which can automatically adapt to a diverging number of parameters.

## 3. Theoretical Properties

Here we establish that the proposed ICR estimator has the well-desired estimation consistency, model selection consistency, and clustering consistency properties. Although sharing some similar spirit with the existing studies, with a significantly different problem and penalized estimation, our theoretical development can have a unique value.

### 3.1 Notations and Definitions

For a vector $z=\left(z_{1},\ldots ,z_{p}\right)\in R^{p}$, and 1≤l<∞, define $\|z{\|}_{l}={\left(\sum_{j=1}^{p}{\left|z_{j}\right|}^{l}\right)}^{1/l}$ and $\|z{\|}_{\infty }={\text{max}}_{j\in [p]}\left|z_{j}\right|$. Given an index set 𝒮, let $z_{𝒮}$ denote the subvector of z corresponding to the elements of 𝒮. For a matrix $Z_{s\times p}$, let $\|Z{\|}_{2}={\text{sup}}_{v\in R^{p},\|v{\|}_{2}=1}\|Zv{\|}_{2},\|Z{\|}_{\infty }={\text{max}}_{1\leq i\leq s}\sum_{j=1}^{p}\left|Z_{ij}\right|,\|Z{\|}_{\text{max}}={\text{max}}_{1\leq i\leq s,1\leq j\leq p}\left|Z_{ij}\right|$, and $\|Z{\|}_{F}=\sqrt{\sum_{i=1}^{s}\sum_{j=1}^{p}Z_{ij}^{2}}$. For two index sets ${𝒮}_{1}$ and ${𝒮}_{2}$, let $Z_{{𝒮}_{1}{𝒮}_{2}}$ denote the submatrix of Z corresponding to the rows in ${𝒮}_{1}$ and columns in ${𝒮}_{2}$, and $Z_{{𝒮}_{1}}$ denote the submatrix of Z corresponding to the rows in ${𝒮}_{1}$. For a vector $v_{0}\in R^{p}$, let ${ℬ}_{r}\left(v_{0}\right)=\left\{v\in R^{p}:{\left\|v-v_{0}\right\|}_{2}\leq r\right\}$ be the $\ell_{2}$-ball around $v_{0}$ with radius r>0. For a random variable X, its sub-Gaussian norm is defined by $\|X{\|}_{\psi_{2}}={\text{sup}}_{s\geq 1}s^{-1/2}{\left(E|X{|}^{s}\right)}^{1/s}$. For a random vector $z\in R^{p}$, its sub-Gaussian norm is defined by $\|\bar{z}{\|}_{\psi_{2}}={\text{sup}}_{v\in {ℬ}_{1}(0)}{\left\|v^{⊤}z\right\|}_{\psi_{2}}$. For a symmetric matrix H, its maximum and minimum eigenvalues are denoted by $\Lambda_{\text{max}}(H)$ and $\Lambda_{\text{min}}(H)$, respectively. For two sequences of real numbers $\left\{a_{n}\right\}\geq 1$ and $\left\{b_{n}\right\}\geq 1,b_{n}\ll a_{n}$ (or $b_{n}=o\left(a_{n}\right)$) means that ${\text{lim}\;\text{sup}}_{n\to \infty }b_{n}/a_{n}=0,b_{n}≲a_{n}$ (or $b_{n}=O\left(a_{n}\right)$) means that ∃C>0 such that $b_{n}\leq Ca_{n}$ for all n, and we use $a_{n}≍b_{n}$ if $a_{n}≲b_{n}$ and $b_{n}≲a_{n}$. Similarly, we let $o_{p}(\cdot )$ and $O_{p}(\cdot )$ represent each of the corresponding rates with probability approaching 1 as n→∞. Let $f^{\prime }(a,y)=\partial f(a,y)/\partial a$ and $f^{\prime \prime }(a,y)=\partial^{2}f(a,y)/\partial a^{2}$, where ∂f(a,y)/∂a and $\partial^{2}f(a,y)/\partial a^{2}$ denote the first and second order derivatives of f(a,y) with respect to a, respectively.

Let ${ℳ}_{𝒢}$ be a subspace of $R^{p\times K}$ defined as

$${ℳ}_{𝒢}=\left\{\theta \in R^{p\times K}:\theta^{\left(k\right)}=\psi^{\left(m\right)},\;\text{for}\;\text{any}\;k\in {𝒢}^{\left(m\right)},1\leq m\leq M\right\},$$

where $\psi^{\left(m\right)}$ is the distinct coefficient vector for the mth cluster. Further, we define the p×M common coefficient matrix $\psi =\left(\psi^{\left(1\right)},\ldots ,\psi^{\left(M\right)}\right)={\left(\psi_{1},\ldots ,\psi_{p}\right)}^{⊤}$, where $\psi^{\left(m\right)}={\left(\psi_{1}^{\left(m\right)},\ldots ,\psi_{p}^{\left(m\right)}\right)}^{⊤}$ and $\psi_{j}={\left(\psi_{j}^{\left(1\right)},\ldots ,\psi_{j}^{\left(M\right)}\right)}^{⊤}$. Let $\theta^{*}$ and $\psi^{*}$ be the true coefficient matrices corresponding to θ and ψ, respectively. Without loss of generality, assume the first q groups of covariates have nonzero effects, and the rest (p−q) have zero effects. Let 𝒜={1,…,q} and ${𝒜}^{c}=\{q+1,\ldots ,p\}$. Further, denote $d_{1}={\text{min}}_{j\in 𝒜}{\left\|\psi_{j}^{*}\right\|}_{2}$ and $d_{2}={\text{min}}_{m,m^{\prime }\in [M],m\neq m^{\prime }}{\left\|\psi_{𝒜}^{*(m)}-\psi_{𝒜}^{*\left(m^{\prime }\right)}\right\|}_{2}$.

Let $V^{\left(k\right)}\left(\theta^{\left(k\right)}\right)=\partial^{2}{\hat{ℒ}}_{k}\left(\theta^{\left(k\right)}\right)/\partial \theta^{\left(k\right)}\partial \theta^{(k)⊤}$ and $g^{\left(k\right)}\left(\theta^{\left(k\right)}\right)=\partial {\hat{ℒ}}_{k}\left(\theta^{\left(k\right)}\right)/\partial \theta^{\left(k\right)}$. We further denote ${\tilde{V}}^{\left(k\right)}=V^{\left(k\right)}\left({\tilde{\theta }}^{\left(k\right)}\right),V^{*(k)}=V^{\left(k\right)}\left(\theta^{*(k)}\right),{\tilde{g}}^{\left(k\right)}=g^{\left(k\right)}\left({\tilde{\theta }}^{\left(k\right)}\right)$ and $g^{*(k)}=g^{\left(k\right)}\left(\theta^{*(k)}\right)$ for simplicity. Let $\varphi^{\left(k\right)}={\left\|E\left(V_{{𝒜}^{c}𝒜}^{*(k)}\right){\left[E\left(V_{𝒜𝒜}^{*(k)}\right)\right]}^{-1}\right\|}_{\infty }$ for any k∈[K] and $\varphi_{\text{max}}={\text{max}}_{k\in [K]}\varphi^{\left(k\right)}$. Let $N_{m}=\sum_{k\in {𝒢}^{\left(m\right)}}n_{k},N_{\text{max}}={\text{max}}_{m\in [M]}N_{m}$, and $N_{\text{min}}={\text{min}}_{m\in [M]}N_{m}$. Let $\left|{𝒢}^{\left(m\right)}\right|$ be the cardinality of index set ${𝒢}^{\left(m\right)}$ with m∈[M], and denote $\left|{𝒢}_{\text{max}}\right|={\text{max}}_{m\in [M]}\left|{𝒢}^{\left(m\right)}\right|$ and $\left|{𝒢}_{\text{min}}\right|={\text{min}}_{m\in [M]}\left|{𝒢}^{\left(m\right)}\right|$.

When the underlying true clustering structure $𝒢=\left\{{𝒢}^{\left(1\right)},\ldots ,{𝒢}^{\left(M\right)}\right\}$ is known, we can define the cluster-oracle objective function for θ by

(8) $$\underset{\theta \in {ℳ}_{𝒢}}{\text{arg}\;\text{min}}\left\{ℒ(\theta )=\frac{1}{N}\sum_{k=1}^{K}n_{k}\left(\theta^{(k)⊤}{\tilde{V}}^{\left(k\right)}\theta^{\left(k\right)}-2\theta^{(k)⊤}{\tilde{\zeta }}^{\left(k\right)}\right)+\sum_{j=2}^{p}p_{\tau }\left({\left\|\theta_{j}\right\|}_{2},\lambda_{1}\right)\right\}.$$

Accordingly, the cluster-oracle objective function for the common coefficient matrix ψ is

(9) $${ℒ}^{𝒢}\left(\psi \right)=\frac{1}{N}\sum_{m=1}^{M}\left[\psi^{\left(m\right)⊤}\left(\sum_{k\in {𝒢}^{\left(m\right)}}n_{k}{\tilde{V}}^{\left(k\right)}\right)\psi^{\left(m\right)}-2\psi^{\left(m\right)⊤}\left(\sum_{k\in {𝒢}^{\left(m\right)}}n_{k}{\tilde{\zeta }}^{\left(k\right)}\right)\right]+\sum_{j=2}^{p}p_{\tau }\left(\sqrt{\sum_{m=1}^{M}{\left({\left|{𝒢}^{\left(m\right)}\right|}^{1/2}\psi_{j}^{\left(m\right)}\right)}^{2}},\lambda_{1}\right).$$

### 3.2 Asymptotic Properties

Assume that $n_{k}≍N/K$ for k∈[K], and we denote $n^{*}≍n_{k}$. We further assume the following mild conditions.

(C1) For each k∈[K] and $i\in \left[n_{k}\right],\left\{x_{i}^{\left(k\right)},y_{i}^{\left(k\right)}\right\}$’s are independent and identically distributed. There exists a constant $C_{x}>0$ such that ${\text{max}}_{k\in [K],i\in \left[n_{k}\right]}{\left\|x_{i}^{\left(k\right)}\right\|}_{\infty }\leq C_{x}$ and ${\text{max}}_{x\in {ℬ}_{1}(0)}E{\left(x^{⊤}x_{i}^{\left(k\right)}\right)}^{2}\leq C_{x}^{2}$.
(C2) For each k∈[K] and $i\in \left[n_{k}\right],f^{\prime }\left(\theta^{*(k)⊤}x_{i}^{\left(k\right)},y_{i}^{\left(k\right)}\right)$’s are sub-Gaussian. That is, there exists a constant $\kappa_{x}>0$ such that ${\left\|f^{\prime }\left(\theta^{*(k)⊤}x_{i}^{\left(k\right)},y_{i}^{\left(k\right)}\right)\right\|}_{\psi_{2}}\leq \kappa_{x}$.
(C3) For each k∈[K], there exist two constants $C_{\text{min}}$ and $C_{\text{max}}$ such that $0<C_{\text{min}}\leq \Lambda_{\text{min}}\;\left(E\left(V_{𝒜𝒜}^{*(k)}\right)\right)\leq \Lambda_{\text{max}}\left(E\left(V_{𝒜𝒜}^{*(k)}\right)\right)\leq C_{\text{max}}$.
(C4) For each k∈[K], if δ=o(1), then there exists a constant $C_{L}>0$ such that
$$\left|f^{\prime \prime }\left(\theta^{(k)⊤}x_{i}^{\left(k\right)},y_{i}^{\left(k\right)}\right)\right|\leq C_{L},\;\text{for}\;\text{all}\;\theta^{\left(k\right)}\in {ℬ}_{\delta }\left(\theta^{*\left(k\right)}\right).$$
  Further, the second-order derivatives are Lipschitz continuous. That is,
$$\left|f^{\prime \prime }(a,y)-f^{\prime \prime }(b,y)\right|\leq C_{L}|a-b|,\;\text{for}\;\text{any}\;a,b,y\in R.$$
(C5) The local estimators satisfy
$$\underset{k\in [K]}{\text{max}}{\left\|{\tilde{\theta }}^{\left(k\right)}-\theta^{*(k)}\right\|}_{2}≍\underset{k\in [K]}{\text{max}}\;n_{k}^{-1/2}{\left\|X^{\left(k\right)}\left({\tilde{\theta }}^{\left(k\right)}-\theta^{*(k)}\right)\right\|}_{2}=O_{p}\left(\sqrt{\frac{q\;\text{log}\;p}{n^{*}}}\right).$$
(C6) The penalty function $p_{\tau }(t,\lambda )$ is non-decreasing and concave in t for t∈[0,∞). For $\tau >0,\lambda^{-1}p_{\tau }(t,\lambda )$ is a constant for all t≥τλ, and $p_{\tau }(0,\lambda )=0$. In addition, $p_{\tau }^{\prime }(t,\lambda )$ exists and is continuous except for a finite number of t values and $\lambda^{-1}p_{\tau }^{\prime }(0+,\lambda )=1$.
(C7) ${\left(\left|{𝒢}_{\text{max}}\right|/\left|{𝒢}_{\text{min}}\right|\right)}^{2}q^{4}\;\text{log}\;p\ll n^{*}$ and K≪p.

Condition (C1) assumes that all covariates are uniformly bounded. Similar conditions have been commonly assumed in the literature, especially including Cai et al. (2022). It is satisfied under many practical scenarios. Condition (C2) controls the tail behavior of $x_{ij}^{\left(k\right)}f^{\prime }(a,y)$ and bounds the random error $g^{*(k)}$. Condition (C3) has been commonly assumed to ensure that the eigenvalues of $E\left(V_{𝒜𝒜}^{*(k)}\right)$ are bounded above and below. The first part of Condition (C4) assumes that the second-order derivatives of the loss function are bounded, and the second part is a Lipschitz condition to ensure that the loss function is sufficiently smooth. Condition (C5) provides the error bounds for the local estimators and similar conditions have been assumed in Cai et al. (2022) and Battey et al. (2018). It is noted that such error bounds have been established in Negahban et al. (2012). Condition (C6) is commonly assumed under high-dimensional settings, and it can be easily verified that both MCP and SCAD satisfy this condition.

It is noted that in Cai et al. (2022) and Jordan et al. (2019), restricted strong convexity has been assumed to derive the upper bound of distributed estimators with full p dimensions. Different from such studies, we follow another framework designed for nonconvex penalties (Fan and Lv, 2011) to study the upper bound of the proposed ICR estimator constrained on the true q-dimensional variables and achieve sparsity by the KKT conditions.

**Theorem 1** *Suppose that Conditions (C1)-(C7) hold*. *If* $\lambda_{1}\gg {\left|{𝒢}_{\text{max}}\right|}^{1/2}r_{1N}+\varphi_{\text{max}}r_{2N},{\left|{𝒢}_{\text{min}}\right|}^{1/2}d_{1}>\tau \lambda_{1}$ *and* $r_{1N}=o(1)$, *then there exists a strictly local minimizer* ${\hat{\psi }}^{\text{or}}$ *of* ${ℒ}^{𝒢}(\psi )$ *in* (9) *such that*

$${\left\|{\hat{\psi }}_{𝒜}^{or}-\psi_{𝒜}^{*}\right\|}_{F}=O_{p}\left(r_{1N}\right),\;P\left({\hat{\psi }}_{{𝒜}^{c}}^{or}=0\right)\to 1\;\text{as}\;N\to \infty ,$$

*where*

$$r_{1N}=\sqrt{\frac{\left(K/\left|{𝒢}_{\text{min}}\right|\right)q}{N_{\text{min}}}}+\frac{\left|{𝒢}_{\text{max}}\right|M^{1/2}q^{3/2}\text{log}\;p}{N_{\text{min}}},$$

$$r_{2N}=\sqrt{\frac{\left(\left|{𝒢}_{\text{max}}\right|/\left|{𝒢}_{\text{min}}\right|\right)M\;\text{log}\;p}{KN}}+\frac{\left(\left|{𝒢}_{\text{max}}\right|/{\left|{𝒢}_{\text{min}}\right|}^{1/2}\right)M^{1/2}q\;\text{log}\;p}{N}.$$

Theorem 1 establishes the estimation consistency and model selection consistency of the cluster-oracle estimator ${\hat{\psi }}^{or}$. Note that the second term of $r_{1N}$ is the additional error due to the aggregation of summary statistics as opposed to raw data. If $M\left|{𝒢}_{\text{max}}\right|=o\left(\sqrt{N/\left[q^{2}(\text{log}\;p{)}^{2}\right]}\right)$, the second term in the error bound can be dominated by the first term, which means that the additional errors are asymptotically negligible. Furthermore, if M is fixed and $\left|{𝒢}_{m}\right|≍K/M$ for m∈[M], then $r_{1N}$ turns to be $\sqrt{q/N}+Kq^{3/2}\;\text{log}\;p/N$. Similarly, the additional errors are asymptotically negligible if $K=o\left(\sqrt{N/q^{2}(\text{log}\;p{)}^{2}}\right)$.

Similar constraints regarding the number of clients and sample sizes of clients are widely recognized in the existing literature (Jordan et al., 2019; Cai et al., 2022). In theory, one-shot algorithms require a sufficient sample size for each client to achieve the same statistical accuracy as centralized algorithms (Battey et al., 2018). However, this condition can be relaxed in communication-iterative algorithms via multiple rounds of communication (Jordan et al., 2019). As a result, if the sample size of each client is not large enough, compared with communication-iterative algorithms with a sufficient number of rounds, one-shot algorithms may yield poorer estimation results. In this paper, the proposed ICR method belongs to one-shot algorithms and then the asymptotic equivalence between ICP and IP holds if such constraint can be satisfied.

Based on Theorem 1 and the equivalence of ℒ(θ) in (8) and ${ℒ}^{𝒢}(\psi )$ in (9), we can similarly construct a strictly local minimizer ${\hat{\theta }}^{\text{or}}$ of ℒ(θ) such that ${\left\|{\hat{\theta }}_{𝒜}^{\text{or}}-\theta_{𝒜}^{*}\right\|}_{F}=O_{p}\left({\left|{𝒢}_{\text{max}}\right|}^{1/2}r_{1N}\right)$ and $P\left({\hat{\theta }}_{{𝒜}^{c}}^{\text{or}}=0\right)\to 1$. Furthermore, in the following Theorem 2, we will show that ${\hat{\theta }}^{\text{or}}$ is a strictly local minimizer of ${\hat{𝒬}}_{\text{ICR}}(\theta )$ in (3) with probability approaching 1. Consequently, with probability approaching 1, the ICR estimator $\hat{\theta }$ is equal to the cluster-oracle estimator ${\hat{\theta }}^{\text{or}}$, which indicates that $\hat{\theta }$ also possesses estimation consistency with the same convergence rate as ${\hat{\theta }}^{\text{or}}$ and model selection consistency. As clustering is based on estimation, we can obtain $P(\hat{M}=M)\to 1$ and $P(\hat{𝒢}=𝒢)\to 1$, which indicates clustering consistency.

**Theorem 2** *Suppose that the conditions of Theorem 1 hold. If* $\lambda_{1}\gg \varphi_{\text{max}}(\text{log}\;p/N{)}^{1/2}$, $d_{2}>\tau \lambda_{2}$ *and* $\lambda_{2}\gg {\left|{𝒢}_{\text{max}}\right|}^{1/2}r_{1N}$, *then there exists a strictly local minimizer* $\hat{\theta }$ *of* ${\hat{𝒬}}_{\text{ICR}}(\theta )$ *in* (3) *such that*

$$P\left(\hat{\theta }={\hat{\theta }}^{or}\right)\to 1\;\text{as}\;N\to \infty .$$

It is noted that, compared to the local estimators with convergence rate $O_{p}\left(\sqrt{q\;\text{log}\;p/n^{*}}\right)$ as shown in Condition (C5), each ${\hat{\theta }}^{\left(k\right)}$ possesses a much faster convergence attributable to information aggregation across clients sharing common coefficients. To see this, note that if $\left|{𝒢}_{m}\right|≍K/M$ for m∈[M] and $M≍\text{log}\;p,r_{1N}$ turns to be $\sqrt{q\;\text{log}\;p/N_{\text{min}}}$. This rate is the same as that of the cluster-oracle estimator ${\hat{\theta }}^{\text{or}(k)}$, which has a $\sqrt{\left|{𝒢}_{\text{min}}\right|}$ times faster convergence compared to the local estimators.

## 4. Simulation Study

We conduct abundant simulations to gauge the performance of the proposed approach. For benchmarking, we consider three classes of alternatives, which include local methods, homogeneous integrative methods (homoIM), and heterogeneous integrative methods (heterIM). A straightforward local method is (a) the Local estimator obtained by minimizing a local penalized loss function for each client separately. For the class of homoIM, we consider (b) the distributed least square approximation (DLSA) estimator (Zhu et al., 2021); and (c) the weighted one-shot distributed ridge (WONDER) estimator (Dobriban and Sheng, 2020). For the class of heterIM, we consider (d) the Sparse K-means (SK) estimator obtained by applying the sparse K-means approach (Witten and Tibshirani, 2010) to the local estimators in (a), of which the process can be achieved via R package *sparcl*; (e) the clustered federated learning (CFL) estimator (Ghosh et al., 2020); (f) the data-Shielding High dimensional Integrative Regression (SHIR) estimator Cai et al. (2022); and (g) the Sparse Meta-Analysis (SMA) estimator (He et al., 2016) obtained after executing the sure independent screening procedure (Fan and Lv, 2008) to reduce dimension to n/(3logn) as recommended by He et al. (2016). For the SK estimator, we adopt two criteria, namely the Hartigan statistic (Hartigan, 1975) and gap statistic (Tibshirani et al., 2001), to choose the number of clusters – this is realized using R package NbClust. The corresponding two variants are referred to as SK(har) and SK(gap), respectively. For the CFL estimator, we separately analyze one-shot CFL (OCFL) and iterative CFL with multiple rounds (ICFL), where the number of clusters is specified as the true value for them. Here, both ICFL and OCFL correspond to Algorithm 2 of Ghosh et al. (2020), but the former sets the number of communication rounds as R=100, while the latter sets R=1. Since WONDER and CFL methods all focus on dense estimation, for comparison in variable selection, we apply a hard threshold of 0.1 to their dense estimators for obtaining sparse estimates. For the alternatives, tuning parameters are chosen in a way compatible with the proposed.

In addition to these alternatives, we also consider two ideal golden methods, including (h) the Oracle estimator obtained by minimizing objective function ${ℒ}^{\text{or},𝒢}(\psi )$ in (A.3); and (i) the ideal pooling (IP) estimator obtained by minimizing objective function (1). Note that the Oracle method is not realistic in practice and the IP method is not feasible in a distributed framework. Here, they serve as the ideal targets and help to verify the theoretical properties established in Section 3.

### 4.1 Simulation Settings

In this subsection, we design six examples to observe the performance of the proposed method and the other alternatives. Examples 1–2 and 5–6 are on logistic regression and logistic loss, and Examples 3–4 are on linear regression and squared loss. The true number of clusters is M=2 in Examples 1 and 3, and M=4 in Examples 2, 4 and 5. For Examples 1–4, we let $n_{1}=\cdots =n_{K}=n$. Example 5 is an imbalanced setting with varying $n_{k}$. To match the real data of the anomaly detection study (used in Section 5), we further design Example 6, in which both the sample sizes and the cluster sizes are imbalanced, and the proportion of anomalies can vary significantly across clients. More specific settings are as follows.

**Example 1.** $\psi^{\left(1\right)}={\left(0.4\times 1_{8}^{⊤},0_{p-8}^{⊤}\right)}^{⊤}$ and $\psi^{\left(2\right)}={\left(-0.4\times 1_{8}^{⊤},0_{p-8}^{⊤}\right)}^{⊤}$. We generate $x_{i,-1}^{\left(k\right)},i\in \left[n_{k}\right],k\in [K]$, where $x_{i,-1}^{\left(k\right)}={\left(x_{i2}^{\left(k\right)},\ldots ,x_{ip}^{\left(k\right)}\right)}^{⊤}$, from a multivariate normal distribution with mean 0 and $\text{cov}\left(X_{w},X_{t}\right)=\rho^{\left|w-t\right|}$ for w,t∈{2,⋯,p} and ρ=0.5. Given $X^{\left(k\right)}$, we generate $Y^{\left(k\right)}$ from a logistic model. We set the number of clients in each cluster as $\left|{𝒢}_{1}\right|=\left|{𝒢}_{2}\right|=K/2$. We further set n=200 and consider K∈{16,32,64} and p∈{100,500}.

**Example 2.** $\psi^{\left(1\right)}={\left(0.6\times 1_{4}^{⊤},-0.6\times 1_{4}^{⊤},0_{p-8}^{⊤}\right)}^{⊤},\psi^{\left(2\right)}=\left(0.6\times 1_{2}^{⊤},-0.6\times 1_{2}^{⊤},0.6\times \right.{\left.1_{2}^{⊤},-0.6\times 1_{2}^{⊤},0_{p-8}^{⊤}\right)}^{⊤},\psi^{\left(3\right)}={\left(-0.6\times 1_{2}^{⊤},0.6\times 1_{2}^{⊤},-0.6\times 1_{2}^{⊤},0.6\times 1_{2}^{⊤},0_{p-8}^{⊤}\right)}^{⊤}$, and $\psi^{\left(4\right)}={\left(-0.6\times 1_{4}^{⊤},0.6\times 1_{4}^{⊤},0_{p-8}^{⊤}\right)}^{⊤}$. We set the number of clients in each cluster as $\left|{𝒢}_{1}\right|=\left|{𝒢}_{2}\right|=\left|{𝒢}_{3}\right|=\left|{𝒢}_{4}\right|=K/4$. $X^{\left(k\right)}$ and $Y^{\left(k\right)}$ are generated in a similar manner as in Example 1. We consider K∈{64,128} and n∈{200,400,800} and set p=100.

**Example 3.** The data generation is the same as in Example 1. The difference is that the response is generated from a linear regression model, where the random error has a normal distribution $𝒩\left(0,\sigma^{2}\right)$. We consider K∈{16,32,64} and σ∈{1,2} and set p=100 and n=100.

**Example 4.** The data generation is the same as in Example 2. The difference is that the response is generated from a linear regression model, where the random error has a normal distribution $𝒩\left(0,\sigma^{2}\right)$. We consider σ∈{1,2} and n∈{100,200,400} and set p=100 and K=64.

**Example 5.** The data generation is the same as in Example 2. We sample each $n_{k}$ from $\left\{n_{0}\cdot a:a\in [U]\right\}$ to allow imbalanced sample sizes across different clients. Here, larger U indicates greater imbalance. We consider U∈{5,10} and p∈{200,500,800} as well as fixed K=40 and $n_{0}=100$.

**Example 6.** We consider M=6 clusters with coefficients $\psi^{\left(1\right)}={\left(1.5,\beta^{(1)⊤},0_{p-11}^{⊤}\right)}^{⊤},\psi^{\left(2\right)}={\left(1.0,\beta^{(2)⊤},0_{p-11}^{⊤}\right)}^{⊤},\psi^{\left(3\right)}={\left(0.5,\beta^{(3)⊤},0_{p-11}^{⊤}\right)}^{⊤},\psi^{\left(4\right)}={\left(-0.5,\beta^{(4)⊤},0_{p-11}^{⊤}\right)}^{⊤},\psi^{\left(5\right)}={\left(-1.0,\beta^{(5)⊤},0_{p-11}^{⊤}\right)}^{⊤}$, and $\psi^{\left(6\right)}={\left(-1.5,\beta^{(6)⊤},0_{p-11}^{⊤}\right)}^{⊤}$, where $\beta^{\left(m\right)}={\left(\beta_{1}^{\left(m\right)},\ldots ,\beta_{10}^{\left(m\right)}\right)}^{⊤}$. Here, for all m∈[M], we let $\beta_{j}^{\left(m\right)}=Z_{1}\cdot \text{sign}(W)$ if j∈[5], and $\beta_{j}^{\left(m\right)}=Z_{2}\cdot \text{sign}(W)$ otherwise, where $Z_{1},Z_{2}$ are normally distributed with $Z_{1}\sim 𝒩\left(0.4,0.1^{2}\right)$ and $Z_{2}\sim 𝒩\left(0.8,0.1^{2}\right)$, and W is uniform distributed with W~𝒰(−0.5,0.5). We set the number of clients in each cluster as $\left|{𝒢}_{1}\right|=\left|{𝒢}_{2}\right|=10,\left|{𝒢}_{3}\right|=\left|{𝒢}_{4}\right|=15$ as well as $\left|{𝒢}_{5}\right|=\left|{𝒢}_{6}\right|=20$. We generate $X^{\left(k\right)}$ and $Y^{\left(k\right)}$ in a similar manner as in Example 1, and each $n_{k}$ is sampled from $\left\{n_{0}\cdot a:a\in [U]\right\}$. We consider $n_{0}\in \{200,400\}$ and set p=100 and U=5.

For each example, we generate 100 replicates. We first observe that the proposed computational algorithm has satisfactory convergence properties. With all of our simulated data sets, convergence is achieved within 100 iterations. Additionally, the proposed approach is computationally affordable. For example, the analysis of one simulated data set under Example 1 with K=32,p=100, and 25 candidate tuning parameter values takes about 3 minutes using a desktop with standard configurations – here we note that penalized fusion estimation is in general computationally more expensive. For evaluation and comparison, we comprehensively consider the following measures. Denote the set of selected variables as $\hat{𝒜}=\left\{j:{\hat{\theta }}_{j}\neq 0\right\}$. For evaluating variable selection accuracy, we consider (1) TPR, the percentage of correctly identified important variables across the K studies; (2) FPR, the percentage of falsely identified important variables across the K studies; and (3) MS, the model size defined by $\text{MS}=\sum_{j=1}^{p}{\hat{q}}_{j}$, where ${\hat{q}}_{j}$ is the number of distinct nonzero coefficients of the jth variable. For evaluating clustering accuracy, we consider: (4) $\hat{M}$, the number of identified clusters; (5) Per, the percentage of fully accurate identification; (6) RI, the Rand Index defined as RI = (TP + TN)/(TP + FP + FN + TN), where TP (true positive) is the number of pairs of data sets from the same cluster classified into the same cluster, and TN (true negative), FP (false positive), and FN (false negative) are defined accordingly. Since Rand index tends to be large even under random clusterings, we also adopt (7) ARI, the adjusted Rand index defined by ARI=(RI−E(RI))/(max(RI)−E(RI)), where E(RI) and max(RI) are the expected value and maximum value of R and index, respectively. Rand index ranges from 0 to 1, adjusted Rand index ranges from −1 to 1, and higher values indicate a better agreement between the identified and true clustering structures. For evaluating estimation, we consider (8) RMSE, the root mean squared error of $\hat{\theta }$ defined as $\text{RMSE}=\sqrt{\sum_{j\in 𝒜}{\left\|{\hat{\theta }}_{j}-\theta_{j}^{*}\right\|}_{2}^{2}/K}$.

### 4.2 Simulations Results

**Figure 2:**
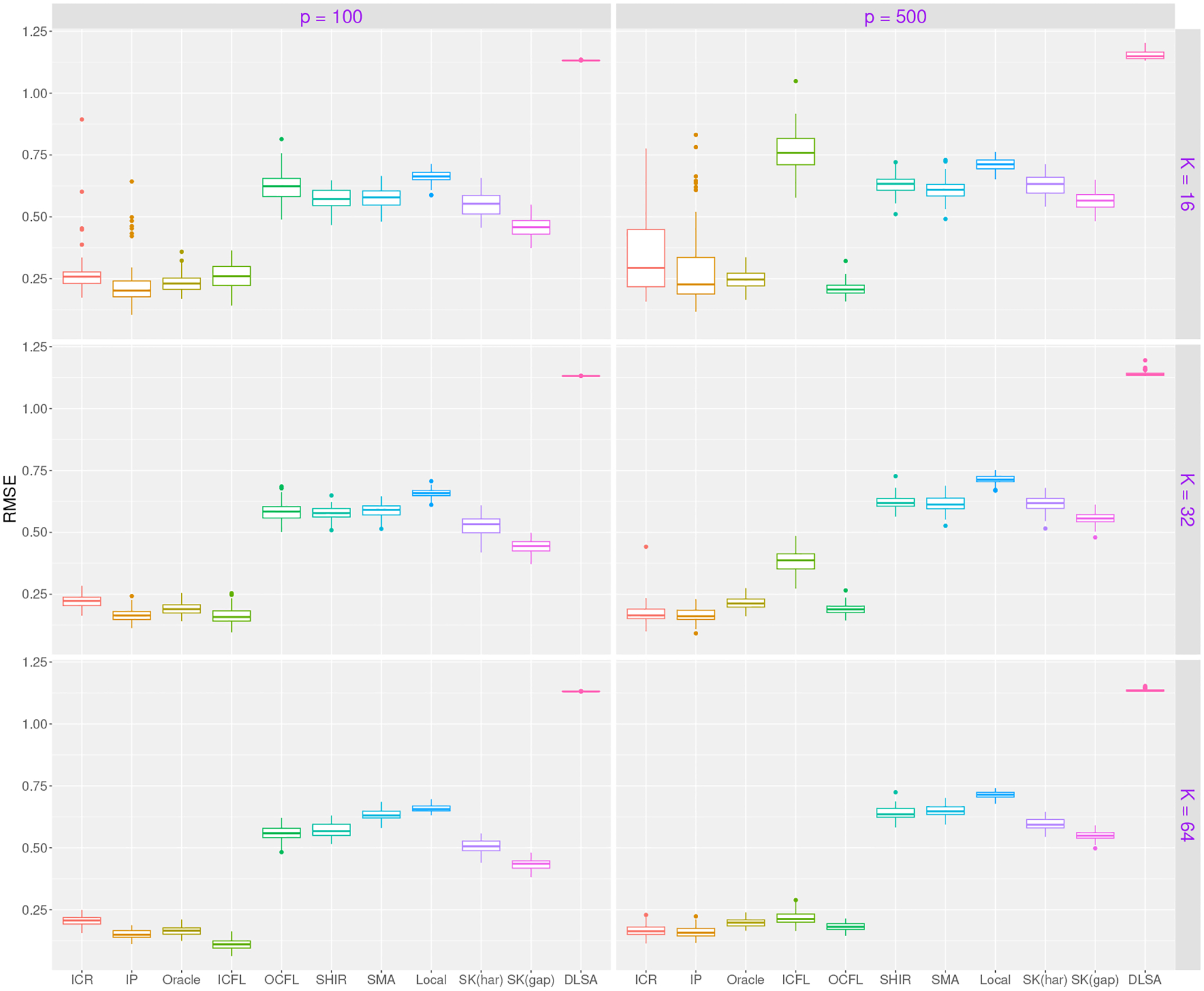
Boxplots of RMSE in Example 1.

Results for Example 1 are summarized in Table 1, Table 2, and Figure 2. It is observed that the proposed ICR approach tends to have larger TPR and smaller FPR values as K increases. It has an average MS value of around 16, which is the true model size, while the alternatives (in the categories of heterIM or local) generate much larger models and DLSA (in the categories of homoIM) generates much smaller models. When K is sufficiently large, it outperforms the alternatives (except for IP) in variable selection. Compared to IP, ICR has slightly worse performance in variable selection and estimation accuracy, which is a reasonable result. Figure 2 suggests that the estimation accuracy of ICR is very close to that of Oracle, especially when K is large, which is consistent with our theoretical results. Compared with ICFL and OCFL (clustered heterIM) with a pre-specified true number of clusters, ICR shows the same performance in the estimation of the number of clusters with $\hat{M}=2$ (the true number of clusters) and clustering accuracy and comparable performance in estimation accuracy. Compared to Local, ICR shows much better performance in both variable selection and estimation accuracy. This further illustrates why we need to integrate clustering structure into distributed learning. Besides, compared to the SK(har) and SK(gap) (two-step clustered heterIM), ICR has better variable selection performance. ICR, ICFL, and OCFL all perform better than SK(har) in clustering and estimation accuracy since SK(har) often overestimates the number of clusters.

Results for Example 2 are summarized in Table 4, Table 5, and Figure 5 (Appendix B). It is observed that ICR also outperforms the other alternatives (except for IP) in variable selection and clustering accuracy and the performance improves as n increases. The performance of ICR approaches IP for larger n, which further verifies the asymptotic equivalence between ICR and IP. In this setting, although both ICFL and OCFL set the correct number of clusters, they show much worse performance in clustering accuracy compared with ICR, which leads to extremely unstable estimation results. Besides, for larger p in Example 5, compared to ICR, both ICFL and OCFL have poorer and more unstable performance (see Figure 8), since the estimation of important variables is significantly influenced by a large number of abundant parameters due to dense estimation. These results explain why we should develop a new clustered distributed learning method to address sparsity issues in high-dimensional data. Compared to Local, SHIR, and SMA have worse estimation performance, which suggests that inappropriate data integration may not help. Table 5 shows that, with ICR and SK(har), the identified number of clusters is close to the true. Further, the RI and ARI values are close to 1, indicating satisfactory clustering performance. In comparison, SK(gap) usually underestimates the number of clusters and has much lower clustering accuracy. Thus, SK methods are very sensitive to the number of clusters. This further illustrates how crucial it is to automatically select the correct value for M. Finally, due to model misspecification, DLSA (in the category of homoIM) has the worst variable selection and estimation performance in both Examples 1 and 2. Since WONDER (also in the category of homoIM), is only feasible in linear regression, so its performance can be observed in Examples 3–4. Similar to DLSA, it also shows much worse performance compared with alternatives in the category of heterIM.

Results for Examples 3–6 are summarized in Tables 6–15 and Figures 6–9 (Appendix B). The overall findings are very similar to Examples 1–2.

## 5. Data Application

With the emergence of technological innovations, cyberattacks (generally carried out by abnormal requests) are becoming increasingly serious and may hinder enterprise operations or interrupt critical infrastructure systems. Web logs, which are generated by systems to record detailed access information, have been widely used to detect abnormal requests in system monitoring and intrusion detection (also called anomaly detection) systems (Hu et al., 2017; Guo et al., 2021; Ünal and Dağ, 2022). Large-scale web logs are usually stored with distributed clients, and the transmission of raw logs from local clients to a central server is often infeasible. As discussed in Guo et al. (2021), on one hand, only a small part of raw logs contains useful information, and hence the full transmission of raw logs, which is very time-consuming, is not necessary. On the other hand, to facilitate log analysis, raw logs are often transferred to third-party analytic services, which increases the risks of privacy leakage. To tackle this problem, Guo et al. (2021) resorted to federated learning for anomaly detection under distributed settings. A limitation of this study is that homogeneity among clients is assumed. Hu et al. (2017) and Ünal and Dağ (2022) pointed out the heterogeneity among clients and constructed client-specific models. This study can be limited with too many redundant parameters. In this section, we apply the proposed method, which takes into consideration both multi-source heterogeneity and estimation efficiency, to a bank website logs data, which is stored in multiple interfaces (clients).

In this analysis, the request type is the binary response and takes values “normal” and “abnormal”. Our goal is to distinguish the abnormal requests from the normal ones based on the log contents, which poses a binary classification problem. There are a total of K=123 URL interfaces, and the sample sizes range from 60 to 25,552. The total sample size is N=306,377, and the overall percentage of abnormal requests is 21.6%. Among the 123 interfaces, 76 have the percentages of abnormal requests equal to 50%, and for the rest 47 interfaces, the percentages range from 1.2% to 75.9%. In Figure 10 (Appendix B), we present the percentages of abnormal requests for these 47 URL interfaces. The significant differences across interfaces suggest the possibility of heterogeneity.

The collected request logs can only be observed in the form of character strings. In particular, each request log contains the interface name and two submitted parameters from “POST” and “GET” access, respectively. In addition, each parameter from POST or GET access consists of a series of key-value pairs separated by “&”. For demonstration, in Table 17, we present one representative record of the initial request logs from URL interface “ajaxNoSessionGetSmsAction”. The unstructured parameters can be difficult to model, and we extract statistical features from the submitted parameters as follows. First, we generate two features, namely Gnum and Pnum, which are defined as the number of GET and POST key-value pairs, respectively. Second, we generate two features, namely Glen and Plen, which are defined as the total length of the GET and POST parameters, respectively. Third, we generate a series of features to measure the lengths of some key-value pairs in the GET and POST parameters, denoted by Glx and Plx, which are defined as the lengths of the (x+1)-th key-value pairs, respectively. Finally, we generate a series of features to measure the types of some key-value pairs in the GET and POST parameters, denoted by Gtx and Ptx, which are defined as the types of the (x+1)-th key-value pairs, respectively. There are three types of key-value pairs, namely “na”, “str”, and “num”, which indicate that the key-value pair is missing, string and numeric, respectively. Take the record in Table 17 (Appendix B) as an example. We can obtain the following feature values: Gnum = 1, Glen = 9, Pnum = 4, Plen = 72, Gl0=9,Gl1=⋯=Gl19=0,Pl0=19,Pl1=16,Pl2=10,Pl3=24,Pl4=⋯=Pl19=0,Gt0=“str”,Gt1=⋯=Gt19=“na”,Pt0=⋯=Pt2=“str”,Pt3=“num”,Pt4=⋯=Pt19=“na”. Since the values of Gl1,…,Gl19 are 0 for all requests, we delete these features. To further utilize the character strings, we concatenate Gt0−Gt19 and Pt0−Pt19 consecutively into a sequence of strings and train the Skip-gram model (which is a popular model of word2vec) to obtain an 80-dimensional continuous word vector features, denoted by GPw1,…,GPw80. Overall, there are 105 features available for analysis.

Prior to analysis, we standardize the p=105 continuous variables to have means 0 and variances 1. The proposed method identifies five nontrivial clusters (with sizes larger than one and denoted as ICR^(1)^, …, ICR^(5)^), which have sizes 37, 30, 11, 7, and 4, respectively. Additionally, there are 34 interfaces forming their own individual clusters. In Table 3, we present important variables identified by the proposed method and/or the four integrative analysis alternatives ICFL, SHIR, SMA, and DLSA, where the DLSA method identifies another 46 important variables (due to space limitation, they are not listed in Table 3). For the ICFL method, we separately pre-specify the number of clusters as 5 or 10 and the corresponding estimators are denoted by ICFL(5) and ICFL(10). Besides, due to space limitation, we only present the cluster-specific parameters ${\text{ICFL}}_{5}^{\left(1\right)},\ldots ,{\text{ICFL}}_{5}^{\left(5\right)}$ (with cluster sizes 47, 39, 25, 9 and 3) corresponding to 5 clusters in the ICFL(5) method, and the estimated parameters ${\text{ICFL}}_{10}^{\left(1\right)},\ldots ,{\text{ICFL}}_{10}^{\left(9\right)}$ (with cluster sizes 42, 25, 23, 14, 6, 5, 4, 2 and 2) in the ICFL(10) method are reported in Table 16 (Appendix B). The ICFL(10) method identifies a cluster with 0 members, which leads to 9 final clusters. It is observed that different methods lead to quite different identification and selection results. Note that, for the proposed method, we only present estimates for the nontrivial clusters. The results for the trivial clusters are omitted and available from the authors. Besides, to obtain sparse estimates in two ICFL methods, we similarly introduce a hard threshold of 0.1, as in the simulation.

At first, both our method and the two variants of ICFL result in highly imbalanced clustering structures, which partially contribute to the heterogeneity. From Table 3, we can see that, with the proposed method, Gl0,Pl0,Pl1, and Pl2 all have strong positive effects for the five identified clusters, while Gnum, Glen, Pnum, and Plen have heterogeneous effects across the five clusters. For example, Pnum has negative effects in clusters ICR^(3)^, ICR^(4)^ and positive effects in clusters ICR^(1)^, ICR^(2)^, ICR^(5)^. This suggests that requests with a longer length of the first key-value pair in GET and longer lengths of the first three key-value pairs in POST are more likely to be abnormal for most of the interfaces. This can potentially lead to a general security rule for the initial screening of abnormal requests. The heterogeneous security rules for the specific interfaces should be derived cluster-by-cluster. Here we note that the traditional anomaly detection for logs is to extract security rules from samples (El Hadj et al., 2018). Moreover, the numbers of important variables identified by the proposed ICR and the other two heterogeneous integrative methods (SHIR and SMA) are much less than that by the homogeneous DLSA method. Too many selected variables can lead to poor prediction performance, which can be further observed by the latter analysis. Additionally, the proposed analysis can reduce the number of models to 39 (5 clustered ones and 34 individual ones), which corresponds to a lower cost of maintaining models than the client-specific modeling methods (with 123 models).

**Figure 3:**
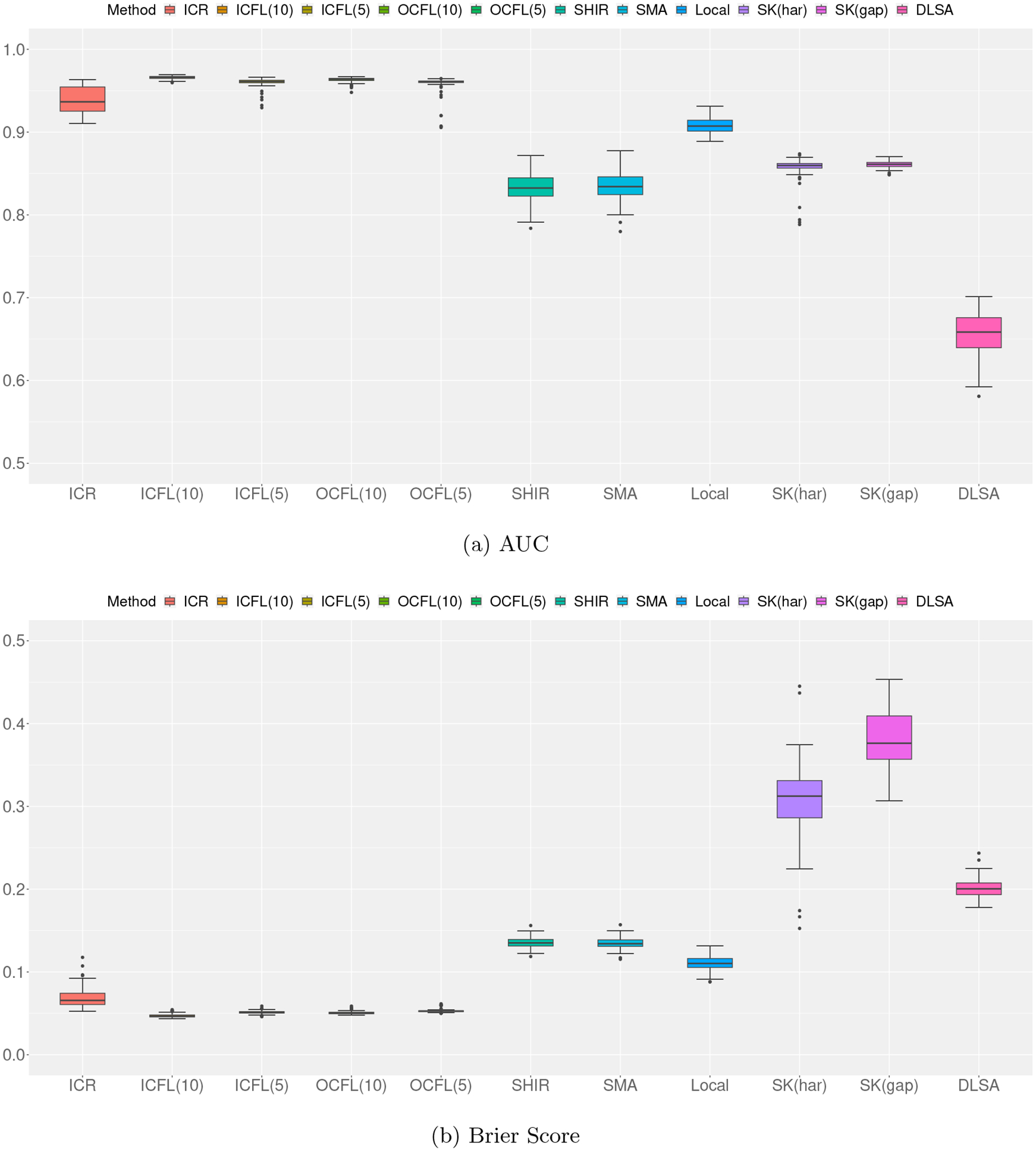
Boxplots of (a) AUC and (b) Brier Score based on 100 random splits in data analysis.

**Figure 4:**
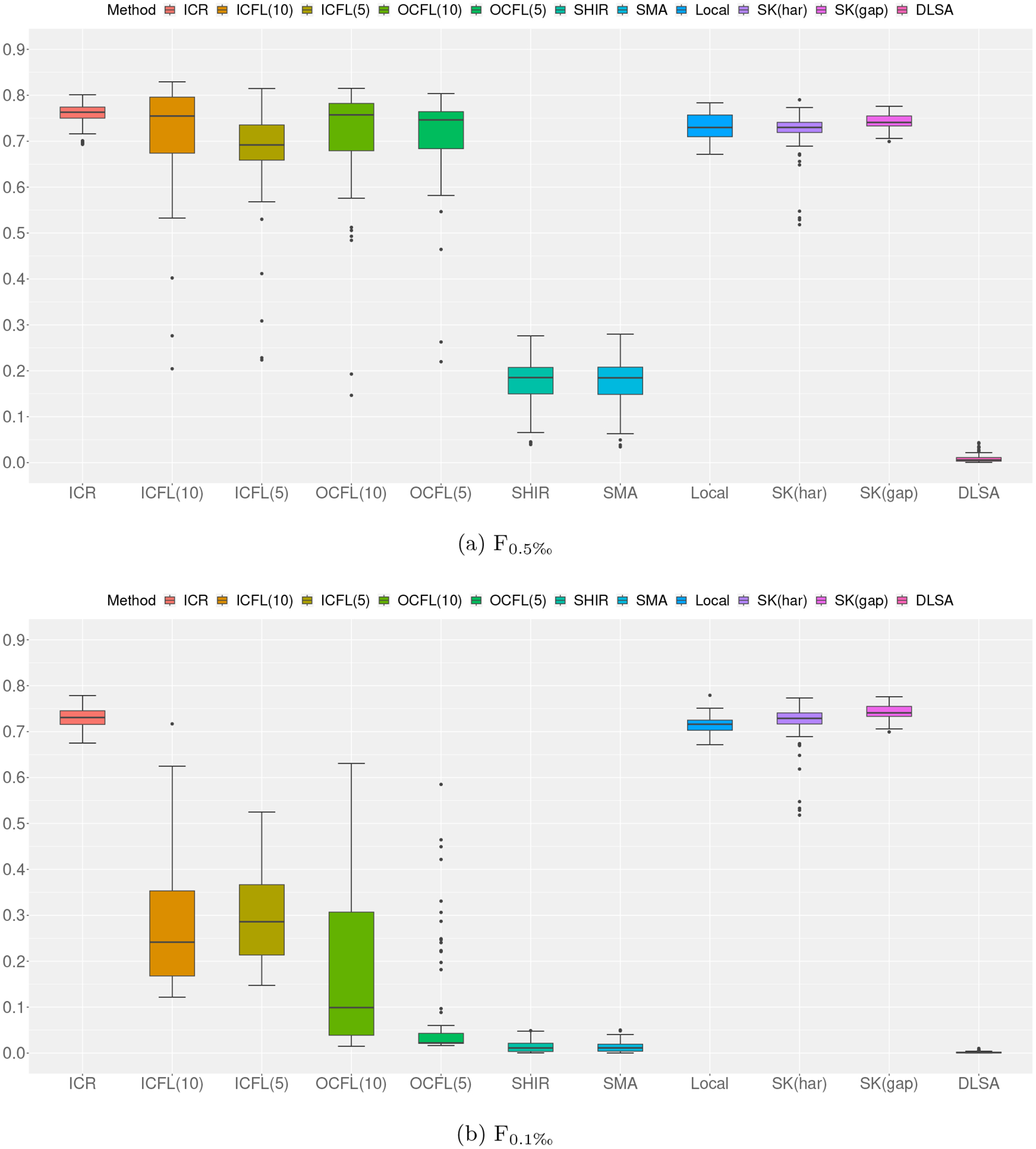
Boxplots of (a) F_0.5‰_ and (b) F_0.1‰_ based on 100 random splits in data analysis.

With practical data, it is difficult to objectively evaluate identification and estimation results. To support our findings, we conduct a prediction evaluation. Specifically, we randomly select 4/5 of the samples and form the training data. In this selection, the normal: abnormal ratio is retained. The remaining samples form the testing data. Estimation is conducted using the training data, and we evaluate prediction performance on the testing data via several accuracy measures, which include the area under the receiver operating characteristic curve (AUC), the Brier score defined as the mean squared residuals, as well as the $F_{1}$-score at threshold value chosen to attain a false positive rate of 0.1‰ and 0.5‰ (denoted by F_0.1‰_ and F_0.5‰_). The $F_{1}$-score is defined as the harmonic mean of the sensitivity and positive predictive value. Note that AUC and the Brier score measure the prediction accuracy across the entire range of class distributions and error costs, while $F_{1}$-score is used to evaluate the prediction accuracy under a deterministic class distribution, which is usually obtained after using a cutoff for the predicted probabilities to enable the separation of the positive and negative classes. For comparison, we also consider the OCFL method with the pre-specified number of clusters 5 or 10, denoted by OCFL(5) and OCFL(10), respectively. This process is repeated 100 times, and the results are summarized in Figures 3 and 4.

It is observed that the proposed method outperforms all alternatives except for the two variants of ICFL and OCFL (referred to as CFLs), in terms of AUC, brier score as well as two $F_{1}$-scores. From the AUC and Brier score, the CFLs show slightly better performance than the proposed method. Nevertheless, two $F_{1}$-scores reveal a completely different phenomenon. Specifically, by Figure 4(a), the proposed method outperforms the CFLs in terms of F_0.5‰_, meanwhile, the CFLs exhibit significant volatility, which is consistent with their simulation results. Moreover, Figure 4(b) shows that this situation appears much more severe on the F_0.1‰_. However, the proposed method shows much higher and more stable $F_{1}$-scores even if the FPR is controlled in 0.1‰. Therefore, in terms of overall prediction performance, the proposed method also beats the CFLs. Additionally, the Local method also has competitive performance, which suggests that inappropriate integration may lead to inferior prediction. Compared to the Local method, the proposed one can have better interpretability and prediction performance.

## 6. Conclusion

In this article, we have developed a new integrative data analysis method that is based on summary statistics and hence can sufficiently protect the privacy of individual clients’ data. The most significant advancement is that it allows for data/model heterogeneity and can automatically identify the underlying clustering structure. Our rigorous theoretical investigation has shown that the proposed method has multiple much-desired consistency properties. Additionally, simulation and data analysis have shown its competitive numerical performance.

This study can be potentially extended in multiple directions. The same as the existing one-shot methods, the proposed analysis only demands one communication round between the local clients and the central server. Our Theorem 1 suggests that the additional error due to the aggregation of summary statistics is asymptotically negligible when we properly restrict the divergence rate of K. If we allow multiple communication rounds (which may lead to higher computational costs), this condition can be relaxed, and there is also a possibility of further improving numerical performance. The proposed method demands mild conditions on the lack-of-fit function. In numerical studies, we have investigated the logistic and linear regressions, which are involved in the framework of generalized linear models. The proposed strategy can be potentially applied to much broader models/loss functions. For example, given the loss function specified as $f\left(\theta^{(k)⊤}x_{i}^{\left(k\right)},y_{i}^{\left(k\right)}\right)$, we can further extend it to survival data with Cox proportional hazards model (Li et al., 2023) and others. Besides, if we consider a more general loss function $f\left(\theta^{\left(k\right)},x_{i}^{\left(k\right)},y_{i}^{\left(k\right)}\right)$, we can extend the proposed method to generalized additive model (Wood, 2017) or generalized additive partial linear model (Wang et al., 2011) for heterogeneity identification.

Another possible extension, as previously mentioned, is to consider different sparsity structures. For example, two-level penalized selection (Huang et al., 2017) can be conducted to allow different sparsity structures for multiple clients. Besides, for clients in which the observed variables have a spatial or temporal order, the same sparsity for a group of adjacent variables within a client can be further assumed (Li and Sang, 2019; Park et al., 2023). These aforementioned extensions will be postponed for future research.

## Supplementary Material

1

## Figures and Tables

**Table 1:** The clustering accuracy: mean (sd) based on 100 replicates in Example 1.

	Method	K=16				K=32				K=64			
		$\hat{M}$	Per	RI	ARI	$\hat{M}$	Per	RI	ARI	$\hat{M}$	Per	RI	ARI
p=100	ICR	**2.000**	**1.000**	**1.000**	**1.000**	**2.000**	**1.000**	**1.000**	**1.000**	**2.000**	**1.000**	**1.000**	**1.000**
		(0.000)	(-)	(0.000)	(0.000)	(0.000)	(-)	(0.000)	(0.000)	(0.000)	(-)	(0.000)	(0.000)
	IP	**2.000**	**1.000**	**1.000**	**1.000**	**2.000**	**1.000**	**1.000**	**1.000**	**2.000**	**1.000**	**1.000**	**1.000**
		(0.000)	(-)	(0.000)	(0.000)	(0.000)	(-)	(0.000)	(0.000)	(0.000)	(-)	(0.000)	(0.000)
	ICFL	**2.000**	**1.000**	**1.000**	**1.000**	**2.000**	**1.000**	**1.000**	**1.000**	**2.000**	**1.000**	**1.000**	**1.000**
		(0.000)	(-)	(0.000)	(0.000)	(0.000)	(-)	(0.000)	(0.000)	(0.000)	(-)	(0.000)	(0.000)
	OCFL	**2.000**	**1.000**	**1.000**	**1.000**	**2.000**	**1.000**	**1.000**	**1.000**	**2.000**	**1.000**	**1.000**	**1.000**
		(0.000)	(-)	(0.000)	(0.000)	(0.000)	(-)	(0.000)	(0.000)	(0.000)	(-)	(0.000)	(0.000)
	SK(har)	4.640	0.000	0.779	0.539	5.230	0.000	0.759	0.508	5.220	0.000	0.734	0.462
		(1.508)	(-)	(0.097)	(0.208)	(1.847)	(-)	(0.098)	(0.202)	(1.495)	(-)	(0.065)	(0.133)
	SK(gap)	**2.000**	**1.000**	**1.000**	**1.000**	**2.000**	**1.000**	**1.000**	**1.000**	**2.000**	**1.000**	**1.000**	**1.000**
		(0.000)	(-)	(0.000)	(0.000)	(0.000)	(-)	(0.000)	(0.000)	(0.000)	(-)	(0.000)	(0.000)
p=500	ICR	**2.000**	**1.000**	**1.000**	**1.000**	**2.000**	**1.000**	**1.000**	**1.000**	**2.000**	**1.000**	**1.000**	**1.000**
		(0.000)	(-)	(0.000)	(0.000)	(0.000)	(-)	(0.000)	(0.000)	(0.000)	(-)	(0.000)	(0.000)
	IP	**2.000**	**1.000**	**1.000**	**1.000**	**2.000**	**1.000**	**1.000**	**1.000**	**2.000**	**1.000**	**1.000**	**1.000**
		(0.000)	(-)	(0.000)	(0.000)	(0.000)	(-)	(0.000)	(0.000)	(0.000)	(-)	(0.000)	(0.000)
	ICFL	**2.000**	**1.000**	**1.000**	**1.000**	**2.000**	**1.000**	**1.000**	**1.000**	**2.000**	**1.000**	**1.000**	**1.000**
		(0.000)	(-)	(0.000)	(0.000)	(0.000)	(-)	(0.000)	(0.000)	(0.000)	(-)	(0.000)	(0.000)
	OCFL	**2.000**	**1.000**	**1.000**	**1.000**	**2.000**	**1.000**	**1.000**	**1.000**	**2.000**	**1.000**	**1.000**	**1.000**
		(0.000)	(-)	(0.000)	(0.000)	(0.000)	(-)	(0.000)	(0.000)	(0.000)	(-)	(0.000)	(0.000)
	SK(har)	4.580	0.000	0.793	0.570	5.440	0.000	0.754	0.499	5.150	0.000	0.766	0.528
		(1.505)	(-)	(0.101)	(0.216)	(1.684)	(-)	(0.097)	(0.199)	(1.720)	(-)	(0.098)	(0.199)
	SK(gap)	2.010	0.990	0.999	0.999	**2.000**	**1.000**	**1.000**	**1.000**	**2.000**	**1.000**	**1.000**	**1.000**
		(0.100)	(-)	(0.006)	(0.012)	(0.000)	(-)	(0.000)	(0.000)	(0.000)	(-)	(0.000)	(0.000)

**Table 2:** The variable selection accuracy: mean (sd) based on 100 replicates in Example 1.

	Method	K=16			K=32			K=64		
		TPR	FPR	MS	TPR	FPR	MS	TPR	FPR	MS
p=100	ICR	0.990	**0.000**	**15.840**	**1.000**	**0.000**	**16.000**	**1.000**	**0.000**	**16.000**
		(0.058)	(0.000)	(0.929)	(0.000)	(0.000)	(0.000)	(0.000)	(0.000)	(0.000)
	IP	0.990	**0.000**	**15.840**	**1.000**	**0.000**	**16.000**	**1.000**	**0.000**	**16.000**
		(0.038)	(0.000)	(0.615)	(0.000)	(0.000)	(0.000)	(0.000)	(0.000)	(0.000)
	ICFL	**1.000**	0.257	63.320	**1.000**	0.093	33.100	**1.000**	0.017	19.040
		(0.000)	(0.050)	(9.137)	(0.000)	(0.034)	(6.204)	(0.000)	(0.013)	(2.470)
	OCFL	**1.000**	0.337	78.020	**1.000**	0.175	48.260	**1.000**	0.056	26.360
		(0.000)	(0.046)	(8.502)	(0.000)	(0.040)	(7.378)	(0.000)	(0.021)	(3.912)
	SHIR	**1.000**	0.010	128.900	**1.000**	0.008	256.730	**1.000**	0.007	512.670
		(0.000)	(0.012)	(1.087)	(0.000)	(0.009)	(0.863)	(0.000)	(0.010)	(0.922)
	SMA	**1.000**	0.008	128.780	**1.000**	0.007	256.660	**1.000**	0.003	512.920
		(0.000)	(0.011)	(0.991)	(0.000)	(0.008)	(0.742)	(0.000)	(0.007)	(6.402)
	Local	0.889	0.102	264.180	0.893	0.100	524.470	0.891	0.102	1056.990
		(0.026)	(0.020)	(29.586)	(0.018)	(0.013)	(37.805)	(0.013)	(0.009)	(54.532)
	SK(har)	0.985	0.350	173.690	0.996	0.516	268.430	1.000	0.728	379.450
		(0.051)	(0.161)	(57.414)	(0.021)	(0.210)	(87.220)	(0.000)	(0.183)	(104.023)
	SK(gap)	**1.000**	0.571	121.000	**1.000**	0.817	166.400	**1.000**	0.964	193.300
		(0.000)	(0.109)	(20.013)	(0.000)	(0.075)	(13.775)	(0.000)	(0.025)	(4.613)
	DLSA	0.125	0.001	1.060	0.125	**0.000**	1.020	0.125	**0.000**	1.010
		(0.000)	(0.003)	(0.239)	(0.000)	(0.002)	(0.141)	(0.000)	(0.001)	(0.100)
p=500	ICR	0.921	**0.000**	14.740	0.999	**0.000**	**16.000**	**1.000**	**0.000**	16.340
		(0.100)	(0.000)	(1.599)	(0.013)	(0.000)	(0.284)	(0.000)	(0.001)	(0.945)
	IP	0.958	**0.000**	**15.320**	**1.000**	**0.000**	**16.000**	**1.000**	**0.000**	**16.000**
		(0.084)	(0.000)	(1.340)	(0.000)	(0.000)	(0.000)	(0.000)	(0.000)	(0.000)
	ICFL	**1.000**	0.447	455.520	**1.000**	0.190	203.120	**1.000**	0.036	51.680
		(0.000)	(0.028)	(27.275)	(0.000)	(0.021)	(20.981)	(0.000)	(0.009)	(9.197)
	OCFL	**1.000**	0.017	32.660	**1.000**	0.002	17.960	**1.000**	0.001	17.440
		(0.000)	(0.006)	(5.498)	(0.000)	(0.001)	(1.449)	(0.000)	(0.001)	(0.903)
	SHIR	0.999	0.004	129.730	0.999	0.004	257.730	**1.000**	0.002	513.100
		(0.013)	(0.003)	(2.206)	(0.013)	(0.003)	(3.681)	(0.000)	(0.002)	(1.185)
	SMA	0.999	0.004	129.580	**1.000**	0.003	257.230	**1.000**	0.001	512.610
		(0.013)	(0.003)	(2.180)	(0.000)	(0.002)	(1.230)	(0.000)	(0.002)	(0.886)
	Local	0.849	0.034	373.360	0.846	0.033	741.420	0.844	0.034	1493.910
		(0.029)	(0.006)	(49.342)	(0.021)	(0.004)	(71.624)	(0.016)	(0.003)	(107.543)
	SK(har)	0.984	0.119	281.850	0.993	0.181	487.520	**1.000**	0.354	865.340
		(0.052)	(0.067)	(121.622)	(0.043)	(0.106)	(211.896)	(0.000)	(0.180)	(357.825)
	SK(gap)	**1.000**	0.235	248.190	**1.000**	0.418	427.740	**1.000**	0.662	667.620
		(0.000)	(0.061)	(59.641)	(0.000)	(0.061)	(59.943)	(0.000)	(0.050)	(49.399)
	DLSA	0.477	0.540	269.717	0.449	0.531	264.800	0.443	0.510	254.370
		(0.163)	(0.055)	(27.438)	(0.171)	(0.050)	(25.376)	(0.157)	(0.049)	(24.475)

**Table 3:** The identified important variables and their estimates using the five integrative analysis methods in data analysis. For the proposed method, only estimates for the nontrivial clusters are shown.

Variable	ICR^(1)^	ICR^(2)^	ICR^(3)^	ICR^(4)^	ICR^(5)^	${\text{ICFL}}_{5}^{\left(1\right)}$	${\text{ICFL}}_{5}^{\left(2\right)}$	${\text{ICFL}}_{5}^{\left(3\right)}$	${\text{ICFL}}_{5}^{\left(4\right)}$	${\text{ICFL}}_{5}^{\left(5\right)}$	SHIR	SMA	DLSA
Intercept	3.321	−0.303	7.530	−1.003	8.345	1.266	1.572	−2.545	−0.254	0.981	−1.651	−1.764	−0.362
Gnum	−0.437	0.002	−0.025	0.184	−0.093	−0.213	−0.213	0.142	0.819	−0.189	–	–	0.213
Glen	−0.145	0.009	−0.281	0.008	−0.156	−0.233	−0.184	0.117	−0.175	−0.271	−0.057	−0.059	−0.362
Pnum	0.096	0.066	−0.040	−0.114	0.213	0.102	–	−0.120	0.455	–	0.044	0.004	0.139
Plen	−0.087	0.123	−0.507	−0.834	−0.348	−0.358	−0.171	−0.338	0.274	–	−0.034	–	0.070
Gl0	1.686	0.034	1.304	0.622	1.834	1.252	1.535	0.594	−0.528	2.021	0.270	0.159	0.134
Pl0	1.393	0.131	2.266	1.731	2.840	1.661	0.404	1.026	0.452	2.882	0.261	0.179	−0.081
Pl1	4.115	0.338	8.460	4.488	11.026	2.420	2.486	1.778	1.611	1.083	0.575	0.503	0.167
Pl2	3.376	0.264	7.736	6.376	3.162	2.117	2.720	1.712	1.031	0.224	0.478	0.411	0.107
Pl3	0.013	0.013	0.089	0.087	0.061	0.142	–	–	0.112	–	–	–	–
Pl4	0.018	0.030	0.010	−0.031	0.007	–	–	0.101	0.787	–	0.080	0.062	–
Pl5	0.005	0.013	0.004	0.175	0.067	–	0.104	–	0.332	–	0.035	0.024	–
Pl6	0.011	−0.002	0.013	0.041	−0.082	–	–	–	–	–	–	–	–
Pl7	–	–	–	–	–	–	–	–	–	–	–	–	−0.008
Pl8	–	–	–	–	–	–	–	–	−0.685	–	–	–	–
Pl9	–	–	–	–	–	–	–	–	0.257	–	0.017	0.028	–
Pl10	–	–	–	–	–	–	–	–	−0.161	–	0.016	0.004	–
Pl11	–	–	–	–	–	–	–	–	0.123	–	–	–	–
Pl13	–	–	–	–	–	–	–	–	0.143	–	–	–	–
Pl14	–	–	–	–	–	–	–	–	0.134	–	0.013	0.008	−0.011
Pl19	–	–	–	–	–	–	–	–	–	–	–	–	−0.035
GPw1	−0.032	0.017	0.069	0.119	−0.001	–	–	–	–	–	–	–	−0.028
GPw4	–	–	–	–	–	–	–	–	0.113	–	–	–	–
GPw6	0.004	0.011	0.135	−0.028	−0.241	–	–	–	–	–	–	–	0.044
GPw11	–	–	–	–	–	–	–	–	0.140	–	–	–	–
GPw15	–	–	–	–	–	–	–	–	–	–	−0.007	–	−0.024
GPw22	–	–	–	–	–	–	–	–	−0.114	–	–	–	–
GPw26	–	–	–	–	–	–	–	–	–	–	−0.009	–	–
GPw27	–	–	–	–	–	–	–	–	–	–	–	−0.029	–
GPw32	–	–	–	–	–	–	–	–	–	–	0.017	–	−0.036
GPw33	–	–	–	–	–	–	–	–	–	–	−0.010	–	−0.013
GPw34	–	–	–	–	–	–	–	–	–	–	0.006	–	0.049
GPw38	–	–	–	–	–	–	–	–	−0.103	–	–	–	–
GPw39	–	–	–	–	–	–	–	0.118	–	–	–	–	–
GPw40	–	–	–	–	–	–	–	–	–	–	−0.015	–	0.016
GPw43	–	–	–	–	–	–	–	–	−0.150	–	–	–	–
GPw50	–	–	–	–	–	–	–	–	−0.112	–	–	–	–
GPw53	–	–	–	–	–	–	–	–	−0.128	–	–	–	–
GPw54	–	–	–	–	–	–	–	–	−0.103	–	–	–	–
GPw59	–	–	–	–	–	–	–	–	0.112	–	–	–	–
GPw63	–	–	–	–	–	–	–	–	–	–	0.009	–	0.093
GPw64	–	–	–	–	–	–	–	–	–	–	−0.010	–	0.063
GPw68	–	–	–	–	–	–	–	0.111	−0.113	–	–	–	–
GPw74	–	–	–	–	–	–	–	−0.112	–	–	–	–	–
