# Supplementary material for: Heterogeneity-aware Clustered Distributed Learning for Multi-source Data Analysis
Source: J Mach Learn Res. Author manuscript; Available in PMC 2026 Jun 16. (PMC13267879)
Supplement: 1 [file NIHMS2172198-supplement-1.pdf]

Appendix A. Proofs

This section includes two lemmas and the proofs of Theorems 1 and 2.

A.1 Auxiliary Lemmas

Lemma 1 *Suppose that $z_1, \ldots, z_n \in \mathbb{R}$ are independent and centered sub-Gaussian random variables. Let $\mathbf{z} = (z_1, \ldots, z_n)^\top$ and $\kappa = \max_{i\in[n]} \|z_i\|_{\psi_2}$. Then for any $\mathbf{a} = (a_1, \ldots, a_n)^\top \in \mathbb{R}^n$ and $t > 0$, there exists a constant $C_1 > 0$ such that*

$$P(|\mathbf{a}^\top \mathbf{z}| \geq t) \leq 2\exp\bigg(-\frac{C_1 t^2}{\kappa^2\|\mathbf{a}\|_2^2}\bigg).$$

Proof. Lemma 1 follows directly from Lemma 14.3, Chapter 14.2.2 of Bühlmann and Van De Geer (2011). □

Lemma 2 *Under Conditions (C1), (C4), (C5) and (C7), we have*

$$\max_{k\in[K]} \Big\|\widetilde{\mathbf{V}}^{(k)} - \mathbb{E}(\mathbf{V}^{*(k)})\Big\|_{\max} = O_p\left(\sqrt{\frac{q\log p}{n^*}}\right).$$

Proof. Note that,

$$\begin{aligned} &\max_{k\in[K]} \Big\|\widetilde{\mathbf{V}}^{(k)} - \mathbb{E}(\mathbf{V}^{*(k)})\Big\|_{\max} \\ \leq\; &\underbrace{\max_{k\in[K]} \Big\|\widetilde{\mathbf{V}}^{(k)} - \mathbb{E}(\widetilde{\mathbf{V}}^{(k)})\Big\|_{\max}}_{I_1} + \underbrace{\max_{k\in[K]} \Big\|\mathbb{E}(\widetilde{\mathbf{V}}^{(k)}) - \mathbb{E}(\mathbf{V}^{*(k)})\Big\|_{\max}}_{I_2}. \end{aligned} \tag{A.1}$$

At first, we derive the upper bound of I_1. Note that, under Conditions (C5) and (C7), $\max_{k\in[K]} \|\widetilde{\boldsymbol{\theta}}^{(k)} - \boldsymbol{\theta}^{*(k)}\|_2 = o_p(1)$. Then by Conditions (C1) and (C4), for all $k \in [K]$ and $j_1, j_2 \in [p]$, with probability approaching 1,

$$\Big|\widetilde{\mathbf{V}}^{(k)}_{j_1 j_2}\Big| = \Big|n_k^{-1}\sum_{i=1}^{n_k} f''(\widetilde{\boldsymbol{\theta}}^{(k)\top}\boldsymbol{x}_i^{(k)}, y_i^{(k)}) x_{ij_1} x_{ij_2}\Big| \leq C_L C_x^2.$$

Then, for any given $t > 0$, Hoeffding's inequality and the union bound yield that

$$P(I_1 > t) \leq 2Kp^2 \exp\bigg\{-\frac{\min_{k\in[K]} n_k t^2}{2C_x^4 C_L^2}\bigg\},$$

which leads to $I_1 = O_p(\sqrt{\log p/n^*})$.

Next, we derive the bound of I_2. For all $k \in [K]$ and $j_1, j_2 \in [p]$, under Conditions (C1) and (C4),

$$\begin{aligned} \Big|\mathbb{E}(\widetilde{\mathbf{V}}^{(k)}_{j_1 j_2}) - \mathbb{E}(\mathbf{V}^{*(k)}_{j_1 j_2})\Big| &\leq \mathbb{E}\Big[\Big|f''(\widetilde{\boldsymbol{\theta}}^{(k)\top}\boldsymbol{x}_i^{(k)}, y_i^{(k)}) x_{ij_1}x_{ij_2} - f''(\boldsymbol{\theta}^{*(k)\top}\boldsymbol{x}_i^{(k)}, y_i^{(k)}) x_{ij_1}x_{ij_2}\Big|\Big] \\ &\leq C_L C_x^2 \mathbb{E}\Big[\Big|\boldsymbol{x}_i^{(k)\top}(\widetilde{\boldsymbol{\theta}}^{(k)} - \boldsymbol{\theta}^{*(k)})\Big|\Big] \\ &\leq C_L C_x^2 \bigg\{\max_{\boldsymbol{v}\in\mathcal{B}_1(\mathbf{0})} \mathbb{E}\big[(\boldsymbol{v}^\top \boldsymbol{x}_i)^2\big] \Big\|\widetilde{\boldsymbol{\theta}}^{(k)} - \boldsymbol{\theta}^{*(k)}\Big\|_2^2\bigg\}^{1/2} \\ &\leq C_L C_x^3 \Big\|\widetilde{\boldsymbol{\theta}}^{(k)} - \boldsymbol{\theta}^{*(k)}\Big\|_2. \end{aligned}$$

Thus,

$$I_2 \le C_L C_x^3 \max_{k\in[K]} \left\|\widetilde{\boldsymbol{\theta}}^{(k)} - \boldsymbol{\theta}^{*(k)}\right\|_2 = O_p\left(\sqrt{\frac{q\log p}{n^*}}\right).$$

Combining the bounds of I_1 and I_2 as well as (A.1), we can prove the result. □

A.2 Proof of Theorem 1

Recall the definitions of $\mathcal{L}(\boldsymbol{\theta})$ in (8) and $\mathcal{L}^{\mathcal{G}}(\boldsymbol{\psi})$ in (9), we are going to define another two objective functions. If both the true clustering structure and important covariate set are known, we can define the oracle estimator $\widehat{\boldsymbol{\theta}}^{or} = (\widehat{\boldsymbol{\theta}}_{\mathcal{A}}^{or\top}, \mathbf{0}_{(p-q)\times K}^\top)^\top$ for $\boldsymbol{\theta}$ as

$$\mathop{\arg\min}_{\boldsymbol{\theta}\in\mathcal{M}_{\mathcal{G}},\boldsymbol{\theta}_{\mathcal{A}^c}=\mathbf{0}} \mathcal{L}^{or}(\boldsymbol{\theta}) := \frac{1}{N}\sum_{k=1}^K n_k\Big(\boldsymbol{\theta}^{(k)\top}\widetilde{\mathbf{V}}^{(k)}\boldsymbol{\theta}^{(k)} - 2\boldsymbol{\theta}^{(k)\top}\widetilde{\boldsymbol{\zeta}}^{(k)}\Big). \tag{A.2}$$

Accordingly, the oracle estimator $\widehat{\boldsymbol{\psi}}^{or} = (\widehat{\boldsymbol{\psi}}_{\mathcal{A}}^{or\top}, \mathbf{0}_{(p-q)\times M}^\top)^\top$ for $\boldsymbol{\psi}$ can be defined as

$$\mathop{\arg\min}_{\boldsymbol{\psi}\in\mathbb{R}^{p\times M},\boldsymbol{\psi}_{\mathcal{A}^c}=\mathbf{0}} \mathcal{L}^{or,\mathcal{G}}(\boldsymbol{\psi}) := \frac{1}{N}\sum_{m=1}^M \left[\boldsymbol{\psi}^{(m)\top}\left(\sum_{k\in\mathcal{G}^{(m)}} n_k\widetilde{\mathbf{V}}^{(k)}\right)\boldsymbol{\psi}^{(m)} - 2\boldsymbol{\psi}^{(m)\top}\left(\sum_{k\in\mathcal{G}^{(m)}} n_k\widetilde{\boldsymbol{\zeta}}^{(k)}\right)\right]. \tag{A.3}$$

The results in Theorem 1 can be proved via two steps. In Step 1, we want to show that $\|\widehat{\boldsymbol{\psi}}_{\mathcal{A}}^{or} - \boldsymbol{\psi}_{\mathcal{A}}^*\|_2 = O_p(r_{1N})$, where r_{1N} is defined in (A.15). In Step 2, we further show that $\widehat{\boldsymbol{\psi}}^{or}$ is a strictly local minimizer of $\mathcal{L}^{\mathcal{G}}(\boldsymbol{\psi})$ with probability approaching 1. As a result, combining Steps 1 and 2, the sparsity and upper bound of estimation error for the nonzero coefficients can be naturally obtained.

Step 1: Let $\boldsymbol{\psi}_{\mathcal{A}} = (\boldsymbol{\psi}_{\mathcal{A}}^{(1)}, \ldots, \boldsymbol{\psi}_{\mathcal{A}}^{(M)})$ with $\boldsymbol{\psi}_{\mathcal{A}}^{(m)} = (\psi_1^{(m)}, \ldots, \psi_q^{(m)})^\top$. Based on the definition of $\mathcal{L}^{or,\mathcal{G}}(\boldsymbol{\psi})$, we can further define

$$\mathcal{L}_{\mathcal{A}}^{or,\mathcal{G}}(\boldsymbol{\psi}_{\mathcal{A}}) = \frac{1}{N}\sum_{m=1}^M \left[\boldsymbol{\psi}_{\mathcal{A}}^{(m)\top}\left(\sum_{k\in\mathcal{G}^{(m)}} n_k\widetilde{\mathbf{V}}_{\mathcal{A}\mathcal{A}}^{(k)}\right)\boldsymbol{\psi}_{\mathcal{A}}^{(m)} - 2\boldsymbol{\psi}_{\mathcal{A}}^{(m)\top}\left(\sum_{k\in\mathcal{G}^{(m)}} n_k\widetilde{\boldsymbol{\zeta}}_{\mathcal{A}}^{(k)}\right)\right]. \tag{A.4}$$

The solution of (A.4) is denoted by $\widehat{\boldsymbol{\psi}}_{\mathcal{A}}^{or} = (\widehat{\boldsymbol{\psi}}_{\mathcal{A}}^{or(1)}, \ldots, \widehat{\boldsymbol{\psi}}_{\mathcal{A}}^{or(M)})$, and the corresponding true coefficient matrix is denoted by $\boldsymbol{\psi}_{\mathcal{A}}^* = (\boldsymbol{\psi}_{\mathcal{A}}^{*(1)}, \ldots, \boldsymbol{\psi}_{\mathcal{A}}^{*(M)})$. Then we have $\mathcal{L}_{\mathcal{A}}^{or,\mathcal{G}}(\widehat{\boldsymbol{\psi}}_{\mathcal{A}}^{or}) \le \mathcal{L}_{\mathcal{A}}^{or,\mathcal{G}}(\boldsymbol{\psi}_{\mathcal{A}}^*)$, and accordingly,

$$\sum_{m=1}^M \Big(\widehat{\boldsymbol{\psi}}_{\mathcal{A}}^{or(m)\top}\widetilde{\mathbf{V}}_{\mathcal{A}\mathcal{A}}^{\mathcal{G}(m)}\widehat{\boldsymbol{\psi}}_{\mathcal{A}}^{or(m)} - 2\widehat{\boldsymbol{\psi}}_{\mathcal{A}}^{or(m)\top}\widetilde{\boldsymbol{\zeta}}_{\mathcal{A}}^{\mathcal{G}(m)}\Big) \le \sum_{m=1}^M \Big(\boldsymbol{\psi}_{\mathcal{A}}^{*(m)\top}\widetilde{\mathbf{V}}_{\mathcal{A}\mathcal{A}}^{\mathcal{G}(m)}\boldsymbol{\psi}_{\mathcal{A}}^{*(m)} - 2\boldsymbol{\psi}_{\mathcal{A}}^{*(m)\top}\widetilde{\boldsymbol{\zeta}}_{\mathcal{A}}^{\mathcal{G}(m)}\Big), \tag{A.5}$$

where

$$\widetilde{\mathbf{V}}^{\mathcal{G}(m)} = N^{-1}\sum_{k\in\mathcal{G}^{(m)}} n_k\widetilde{\mathbf{V}}^{(k)}, \quad \widetilde{\boldsymbol{\zeta}}^{\mathcal{G}(m)} = N^{-1}\sum_{k\in\mathcal{G}^{(m)}} n_k\widetilde{\boldsymbol{\zeta}}^{(k)}.$$

Motivated by Cai et al. (2022), for a vector or matrix $A(t)$ whose (i,j)th entry $A_{ij}(t)$ is a function of a scalar $t\in[0,1]$, we define $\int_0^1 A(t)dt$ as the vector or matrix with its (i,j)th

entry being $\int_0^1 A_{ij}(t)dt$. Then, we can transform the term $-\widetilde{\boldsymbol{\zeta}}^{(k)}$ in (A.5) into

$$\begin{aligned}\widetilde{\mathbf{g}}^{(k)} - \widetilde{\mathbf{V}}^{(k)}\widetilde{\boldsymbol{\theta}}^{(k)} &= \mathbf{g}^{*(k)} - \widetilde{\mathbf{V}}^{(k)}\boldsymbol{\theta}^{*(k)} \\ &+ \int_0^1 \Big\{\mathbf{V}^{(k)}\Big(\big[\boldsymbol{\theta}^{*(k)} + t(\widetilde{\boldsymbol{\theta}}^{(k)} - \boldsymbol{\theta}^{*(k)})\big]\Big) - \widetilde{\mathbf{V}}^{(k)}\Big\}(\widetilde{\boldsymbol{\theta}}^{(k)} - \boldsymbol{\theta}^{*(k)})\ dt.\end{aligned} \tag{A.6}$$

Plugging (A.6) into (A.5), we have

$$\begin{aligned}&\sum_{m=1}^{M}\Big[\Big(\widehat{\boldsymbol{\psi}}_{\mathcal{A}}^{or(m)} - \boldsymbol{\psi}_{\mathcal{A}}^{*(m)}\Big)^\top \widetilde{\mathbf{V}}_{\mathcal{A}\mathcal{A}}^{\mathcal{G}(m)}\Big(\widehat{\boldsymbol{\psi}}_{\mathcal{A}}^{or(m)} - \boldsymbol{\psi}_{\mathcal{A}}^{*(m)}\Big)\Big] \\ &\le 2\sum_{m=1}^{M}\Big(\boldsymbol{\psi}_{\mathcal{A}}^{*(m)} - \widehat{\boldsymbol{\psi}}_{\mathcal{A}}^{or(m)}\Big)^\top\Big[N^{-1}\sum_{k\in\mathcal{G}^{(m)}} n_k \mathbf{g}^{*(k)}\Big]_{\mathcal{A}} + 2\sum_{m=1}^{M}\Big(\boldsymbol{\psi}_{\mathcal{A}}^{*(m)} - \widehat{\boldsymbol{\psi}}_{\mathcal{A}}^{or(m)}\Big)^\top \\ &\quad \times\Big[N^{-1}\sum_{k\in\mathcal{G}^{(m)}} n_k \int_0^1 \Big\{\mathbf{V}^{(k)}\Big(\big[\boldsymbol{\theta}^{*(k)} + t(\widetilde{\boldsymbol{\theta}}^{(k)} - \boldsymbol{\theta}^{*(k)})\big]\Big) - \widetilde{\mathbf{V}}^{(k)}\Big\}(\widetilde{\boldsymbol{\theta}}^{(k)} - \boldsymbol{\theta}^{*(k)})\ dt\Big]_{\mathcal{A}}.\end{aligned} \tag{A.7}$$

Let $\boldsymbol{\alpha} = \big[\mathrm{vec}(\widehat{\boldsymbol{\psi}}_{\mathcal{A}}^{or}) - \mathrm{vec}(\boldsymbol{\psi}_{\mathcal{A}}^{*})\big]$ and $\widetilde{\mathbf{V}}^{(\mathcal{G},\mathcal{A})} = \mathrm{bdiag}(\widetilde{\mathbf{V}}_{\mathcal{A}\mathcal{A}}^{\mathcal{G}(1)}, \dots, \widetilde{\mathbf{V}}_{\mathcal{A}\mathcal{A}}^{\mathcal{G}(M)})$, where $\mathrm{vec}(\mathbf{A})$ is a vectorization of the matrix $\mathbf{A}$ by columns and $\mathrm{bdiag}(\mathbf{A}_1, \dots, \mathbf{A}_M)$ denotes the block diagonal matrix with the diagonal elements being $\mathbf{A}_1, \dots, \mathbf{A}_M$. Besides, we denote $\boldsymbol{\xi} = \big(\boldsymbol{\xi}^{(1)}, \dots, \boldsymbol{\xi}^{(M)}\big)$ and $\boldsymbol{\eta} = \big(\boldsymbol{\eta}^{(1)}, \dots, \boldsymbol{\eta}^{(M)}\big)$, where

$$\boldsymbol{\xi}^{(m)} = \Big[N^{-1}\sum_{k\in\mathcal{G}^{(m)}} n_k \mathbf{g}^{*(k)}\Big],$$

$$\boldsymbol{\eta}^{(m)} = \Big[N^{-1}\sum_{k\in\mathcal{G}^{(m)}} n_k \int_0^1 \Big\{\mathbf{V}^{(k)}\Big(\big[\boldsymbol{\theta}^{*(k)} + t(\widetilde{\boldsymbol{\theta}}^{(k)} - \boldsymbol{\theta}^{*(k)})\big]\Big) - \widetilde{\mathbf{V}}^{(k)}\Big\}(\widetilde{\boldsymbol{\theta}}^{(k)} - \boldsymbol{\theta}^{*(k)})\ dt\Big].$$

Then by (A.7), we have

$$\boldsymbol{\alpha}^\top \widetilde{\mathbf{V}}^{(\mathcal{G},\mathcal{A})}\boldsymbol{\alpha} \le \big|\boldsymbol{\alpha}^\top \mathrm{vec}(\boldsymbol{\xi}_{\mathcal{A}})\big| + \big|\boldsymbol{\alpha}^\top \mathrm{vec}(\boldsymbol{\eta}_{\mathcal{A}})\big|. \tag{A.8}$$

Since $\boldsymbol{\alpha}^\top \widetilde{\mathbf{V}}^{(\mathcal{G},\mathcal{A})}\boldsymbol{\alpha} \ge \Lambda_{\min}(\widetilde{\mathbf{V}}^{(\mathcal{G},\mathcal{A})})\|\boldsymbol{\alpha}\|_2^2$, (A.8) turns to be

$$\|\boldsymbol{\alpha}\|_2^2 \Lambda_{\min}(\widetilde{\mathbf{V}}^{(\mathcal{G},\mathcal{A})}) \le \big|\boldsymbol{\alpha}^\top \mathrm{vec}(\boldsymbol{\xi}_{\mathcal{A}})\big| + \big|\boldsymbol{\alpha}^\top \mathrm{vec}(\boldsymbol{\eta}_{\mathcal{A}})\big|. \tag{A.9}$$

To obtain the lower bound of $\Lambda_{\min}(\widetilde{\mathbf{V}}_{\mathcal{A}\mathcal{A}}^{(k)})$, noting that for each $k \in [K]$, we have

$$\begin{aligned}\Lambda_{\min}(\widetilde{\mathbf{V}}_{\mathcal{A}\mathcal{A}}^{(k)}) &\ge \Lambda_{\min}\Big[\widetilde{\mathbf{V}}_{\mathcal{A}\mathcal{A}}^{(k)} - \mathbb{E}(\mathbf{V}_{\mathcal{A}\mathcal{A}}^{*(k)})\Big] + \Lambda_{\min}[\mathbb{E}(\mathbf{V}_{\mathcal{A}\mathcal{A}}^{*(k)})] \\ &\ge\ -\Big\|\widetilde{\mathbf{V}}_{\mathcal{A}\mathcal{A}}^{(k)} - \mathbb{E}(\mathbf{V}_{\mathcal{A}\mathcal{A}}^{*(k)})\Big\|_F + \Lambda_{\min}[\mathbb{E}(\mathbf{V}_{\mathcal{A}\mathcal{A}}^{*(k)})].\end{aligned} \tag{A.10}$$

By Lemma 2, since $q^3 \log p \ll n^*$ by Condition (C7), for all $k \in [K]$,

$$\begin{aligned}\Big\|\widetilde{\mathbf{V}}_{\mathcal{A}\mathcal{A}}^{(k)} - \mathbb{E}(\mathbf{V}_{\mathcal{A}\mathcal{A}}^{*(k)})\Big\|_F &\le \Big(q^2 \cdot \max_{k\in[K]}\Big\|\widetilde{\mathbf{V}}^{(k)} - \mathbb{E}(\mathbf{V}^{*(k)})\Big\|_{\max}^2\Big)^{1/2} \\ &= O_p\Big(\sqrt{q^3\log p/n^*}\Big) = o_p(1).\end{aligned} \tag{A.11}$$

With (A.10), (A.11), and Condition (C3), for all $k \in [K]$, with probability approaching 1, $\Lambda_{\min}(\widetilde{\mathbf{V}}_{\mathcal{A}\mathcal{A}}^{(k)}) \geq C_{\min}/2$. Consequently, from (A.9) and the Cauchy-Schwarz inequality,

$$\frac{C_{\min}N_{\min}}{2N}\|\boldsymbol{\alpha}\|_2^2 \leq \|\boldsymbol{\alpha}\|_2 \left\|\mathrm{vec}(\boldsymbol{\xi}_{\mathcal{A}})\right\|_2 + \|\boldsymbol{\alpha}\|_2 \left\|\mathrm{vec}(\boldsymbol{\eta}_{\mathcal{A}})\right\|_2. \tag{A.12}$$

Now we prepare to get the upper bounds of $\|\mathrm{vec}(\boldsymbol{\xi}_{\mathcal{A}})\|_2$ and $\|\mathrm{vec}(\boldsymbol{\eta}_{\mathcal{A}})\|_2$, respectively. For $\|\mathrm{vec}(\boldsymbol{\xi}_{\mathcal{A}})\|_2$, note that the product of a sub-Gaussian random variable and a bounded random variable is also sub-Gaussian and the fact of $\mathbb{E}(n_k\mathbf{g}^{*(k)}) = \mathbb{E}(\mathbf{X}^{(k)\top}\Phi^{(k)})$ with $\Phi^{(k)} = (f'(\boldsymbol{x}_1^{(k)\top}\boldsymbol{\theta}^{*(k)}, y_1^{(k)}), \ldots, f'(\boldsymbol{x}_{n_k}^{(k)\top}\boldsymbol{\theta}^{*(k)}, y_{n_k}^{(k)}))^\top$. Then, by Conditions (C1), (C2), and Lemma 1, for all $m \in [M], j \in [p]$ and any $t > 0$, we have

$$P\left(\frac{1}{\sqrt{N_m}}\left|\sum_{k\in\mathcal{G}^{(m)}} n_k\mathbf{g}_j^{*(k)}\right| \geq t\right) \leq 2\exp\left(-\frac{C_1t^2}{C_x^2\kappa_x^2}\right).$$

Accordingly, there exists a constant $C_2 > 0$ such that

$$\mathbb{E}\left[\left(\sum_{k\in\mathcal{G}^{(m)}} n_k\mathbf{g}_j^{*(k)}\right)^2\right] \leq C_2N_m.$$

Then, by Markov's inequality,

$$\begin{aligned} P\left(\|\mathrm{vec}(\boldsymbol{\xi}_{\mathcal{A}})\|_2^2 \geq t^2\right) &\leq \frac{\sum_{m=1}^M \mathbb{E}(\|\boldsymbol{\xi}_{\mathcal{A}}^{(m)}\|_2^2)}{t^2} \\ &\leq \frac{\sum_{m=1}^M\sum_{j\in\mathcal{A}}\mathbb{E}\left[\left(\sum_{k\in\mathcal{G}^{(m)}} n_k\mathbf{g}_j^{*(k)}\right)^2\right]}{N^2t^2} \leq \frac{C_2q}{Nt^2}, \end{aligned} \tag{A.13}$$

which leads to $\|\mathrm{vec}(\boldsymbol{\xi}_{\mathcal{A}})\|_2 = O_p(\sqrt{q/N})$.

For $\|\mathrm{vec}(\boldsymbol{\eta}_{\mathcal{A}})\|_2$, note that, for all $k \in [K]$ and any $t \in [0,1]$, by Conditions (C1), (C4) and (C5), we have

$$\begin{aligned} &\left\|\left\{\mathbf{V}^{(k)}\left([\boldsymbol{\theta}^{*(k)} + t(\widetilde{\boldsymbol{\theta}}^{(k)} - \boldsymbol{\theta}^{*(k)})]\right) - \widetilde{\mathbf{V}}^{(k)}\right\}(\widetilde{\boldsymbol{\theta}}^{(k)} - \boldsymbol{\theta}^{*(k)})\right\|_\infty \\ &= \left\|\frac{1}{n_k}\sum_{i=1}^{n_k}\boldsymbol{x}_i^{(k)}\boldsymbol{x}_i^{(k)\top}\left\{f''\left([\boldsymbol{\theta}^{*(k)} + t(\widetilde{\boldsymbol{\theta}}^{(k)} - \boldsymbol{\theta}^{*(k)})]^\top\boldsymbol{x}_i^{(k)}, y_i^{(k)}\right) - f''(\widetilde{\boldsymbol{\theta}}^{(k)\top}\boldsymbol{x}_i^{(k)}, y_i^{(k)})\right\}(\widetilde{\boldsymbol{\theta}}^{(k)} - \boldsymbol{\theta}^{*(k)})\right\|_\infty \\ &\leq \frac{\max_{i,j,k}|x_{ij}^{(k)}|}{n_k}\sum_{i=1}^{n_k}\left|(\widetilde{\boldsymbol{\theta}}^{(k)} - \boldsymbol{\theta}^{*(k)})^\top\boldsymbol{x}_i^{(k)}\right| \cdot C_L\left|(1-t)(\widetilde{\boldsymbol{\theta}}^{(k)} - \boldsymbol{\theta}^{*(k)})^\top\boldsymbol{x}_i^{(k)}\right| \\ &\leq \frac{C_LC_x}{n_k}\left\|\mathbf{X}^{(k)}(\widetilde{\boldsymbol{\theta}}^{(k)} - \boldsymbol{\theta}^{*(k)})\right\|_2^2 = O_p\left(\frac{q\log p}{n^*}\right). \end{aligned}$$

Thus, we have

$$\begin{aligned}
\|\mathrm{vec}(\boldsymbol{\eta}_{\mathcal{A}})\|_2 &= \sqrt{\sum_{m=1}^{M}\left\|\boldsymbol{\eta}_{\mathcal{A}}^{(m)}\right\|_2^2} \\
&\le \sqrt{\sum_{m=1}^{M}(\sqrt{q}\|\boldsymbol{\eta}^{(m)}\|_\infty)^2} \\
&= O_p\left(\sqrt{\frac{\sum_{m=1}^{M}|\mathcal{G}^{(m)}|^2 q^3(\log p)^2}{N^2}}\right) \\
&= O_p\left(\frac{M^{1/2}|\mathcal{G}_{\max}|q^{3/2}\log p}{N}\right).
\end{aligned} \tag{A.14}$$

Combining (A.12)–(A.14), we have

$$\|\boldsymbol{\alpha}\|_2 \le O_p(r_{1N}), \tag{A.15}$$

where

$$r_{1N} = \sqrt{\frac{(K/|\mathcal{G}_{\min}|)q}{N_{\min}}} + \frac{|\mathcal{G}_{\max}|M^{1/2}q^{3/2}\log p}{N_{\min}}.$$

Step 2: Let $\widehat{\boldsymbol{\psi}}^{or} = (\widehat{\boldsymbol{\psi}}_{\mathcal{A}}^{or\top}, \mathbf{0}_{(p-q)\times M}^{\top})^{\top}$ and define

$$\mathcal{L}_1^{\mathcal{G}}(\boldsymbol{\psi}) = \frac{1}{N}\sum_{m=1}^{M}\left[\boldsymbol{\psi}^{(m)\top}\left(\sum_{k\in\mathcal{G}^{(m)}} n_k\widetilde{\mathbf{V}}^{(k)}\right)\boldsymbol{\psi}^{(m)} - 2\boldsymbol{\psi}^{(m)\top}\left(\sum_{k\in\mathcal{G}^{(m)}} n_k\widetilde{\boldsymbol{\zeta}}^{(k)}\right)\right].$$

We show that $\widehat{\boldsymbol{\psi}}^{or}$ is a local minimizer of $\mathcal{L}^{\mathcal{G}}(\boldsymbol{\psi})$ in (9) through verifying the KKT conditions

$$\frac{\partial\mathcal{L}_1^{\mathcal{G}}(\widehat{\boldsymbol{\psi}}^{or})}{\partial\boldsymbol{\psi}_j} + \partial p_\tau\left(\sqrt{\sum_{m=1}^{M}\left(|\mathcal{G}^{(m)}|^{1/2}\widehat{\psi}_j^{or(m)}\right)^2}, \lambda_1\right)/\partial\boldsymbol{\psi}_j = \mathbf{0}, \qquad j\in\mathcal{A}, \tag{A.16}$$

$$\left\|\frac{\partial\mathcal{L}_1^{\mathcal{G}}(\widehat{\boldsymbol{\psi}}^{or})}{\partial\boldsymbol{\psi}_j}\right\|_2 \le p_\tau'(0+,\lambda_1)\cdot|\mathcal{G}_{\min}|^{1/2}, \qquad j\in\mathcal{A}^c. \tag{A.17}$$

Note that $\widehat{\boldsymbol{\psi}}_{\mathcal{A}}^{or}$ is the solution of (A.4), then (A.16) holds if we can show that

$$\partial p_\tau\left(\sqrt{\sum_{m=1}^{M}\left(|\mathcal{G}^{(m)}|^{1/2}\widehat{\psi}_j^{or(m)}\right)^2}, \lambda_1\right)/\partial\boldsymbol{\psi}_j = \mathbf{0}.$$

By the properties of penalty function in Condition (C6), it suffices to show

$$\sqrt{\sum_{m=1}^{M}\left(|\mathcal{G}^{(m)}|^{1/2}\widehat{\psi}_j^{or(m)}\right)^2} > \tau\lambda_1, \qquad j\in\mathcal{A}.$$

As a result, the above KKT conditions hold if the following conditions hold

$$|\mathcal{G}_{\min}|^{1/2}\left\|\widehat{\boldsymbol{\psi}}_j^{or}\right\|_2 > \tau\lambda_1, \qquad j \in \mathcal{A}, \tag{A.18}$$

$$\left\|\frac{\partial \mathcal{L}_1^{\mathcal{G}}(\widehat{\boldsymbol{\psi}}^{or})}{\partial \boldsymbol{\psi}_j}\right\|_2 \leq \lambda_1|\mathcal{G}_{\min}|^{1/2}, \qquad j \in \mathcal{A}^c. \tag{A.19}$$

The similar proof logic has also been employed in Huang et al. (2010) and Fan and Lv (2011). At first, we show that Condition (A.18) is satisfied with probability approaching 1. By the triangle inequality and (A.15), when N is sufficiently large,

$$\begin{aligned}|\mathcal{G}_{\min}|^{1/2}\min_{j\in\mathcal{A}}\left\|\widehat{\boldsymbol{\psi}}_j^{or}\right\|_2 &\geq |\mathcal{G}_{\min}|^{1/2}\left(\min_{j\in\mathcal{A}}\|\boldsymbol{\psi}_j^*\|_2 - \max_{j\in\mathcal{A}}\|\widehat{\boldsymbol{\psi}}_j^{or}-\boldsymbol{\psi}_j^*\|_2\right)\\ &\geq |\mathcal{G}_{\min}|^{1/2}\left(\min_{j\in\mathcal{A}}\|\boldsymbol{\psi}_j^*\|_2 - \|\boldsymbol{\alpha}\|_2\right) \geq |\mathcal{G}_{\min}|^{1/2}(d_1 - Cr_{1N}) > \tau\lambda_1,\end{aligned}$$

where C is a constant. The last inequality is satisfied since $|\mathcal{G}_{\min}|^{1/2}d_1 > \tau\lambda_1$ and $\lambda_1 \gg |\mathcal{G}_{\min}|^{1/2}r_{1N}$. Accordingly, (A.18) is satisfied with probability approaching 1 when $N \to \infty$.

Second, we show that Condition (A.19) is satisfied with probability approaching 1. Note that $\left\|\partial\mathcal{L}_1^{\mathcal{G}}(\widehat{\boldsymbol{\psi}}^{or})/\partial\boldsymbol{\psi}_j\right\|_2 = \sqrt{\sum_{m=1}^M(\partial\mathcal{L}_1^{\mathcal{G}}(\widehat{\boldsymbol{\psi}}^{or})/\partial\boldsymbol{\psi}_j^{(m)})^2}$. Then Condition (A.19) holds if

$$\left\|\frac{\partial \mathcal{L}_1^{\mathcal{G}}(\widehat{\boldsymbol{\psi}}^{or})}{\partial \boldsymbol{\psi}_{\mathcal{A}^c}^{(m)}}\right\|_\infty \leq \lambda_1(|\mathcal{G}_{\min}|/M)^{1/2}, \quad m \in [M]. \tag{A.20}$$

Since for each $m \in [M]$, $\partial\mathcal{L}^{or,\mathcal{G}}(\widehat{\boldsymbol{\psi}}_{\mathcal{A}}^{or})/\partial\boldsymbol{\psi}_{\mathcal{A}}^{(m)} = \mathbf{0}$, we have

$$\widehat{\boldsymbol{\psi}}_{\mathcal{A}}^{or(m)} - \boldsymbol{\psi}_{\mathcal{A}}^{*(m)} = \left(\widetilde{\mathbf{V}}_{\mathcal{A}\mathcal{A}}^{\mathcal{G}(m)}\right)^{-1}(\boldsymbol{\xi}_{\mathcal{A}}^{(m)} + \boldsymbol{\eta}_{\mathcal{A}}^{(m)}), \quad m \in [M]. \tag{A.21}$$

Thus, combining (A.20) and (A.21) leads to

$$\begin{aligned}\frac{1}{2}\left\|\frac{\partial \mathcal{L}_1^{\mathcal{G}}(\widehat{\boldsymbol{\psi}}^{or})}{\partial \boldsymbol{\psi}_{\mathcal{A}^c}^{(m)}}\right\|_\infty &= \left\|\widetilde{\mathbf{V}}_{\mathcal{A}^c\mathcal{A}}^{\mathcal{G}(m)}(\widehat{\boldsymbol{\psi}}_{\mathcal{A}}^{or(m)} - \boldsymbol{\psi}_{\mathcal{A}}^{*(m)}) + (\boldsymbol{\xi}_{\mathcal{A}^c}^{(m)} + \boldsymbol{\eta}_{\mathcal{A}^c}^{(m)})\right\|_\infty\\ &\leq \left\|\widetilde{\mathbf{V}}_{\mathcal{A}^c\mathcal{A}}^{\mathcal{G}(m)}\left(\widetilde{\mathbf{V}}_{\mathcal{A}\mathcal{A}}^{\mathcal{G}(m)}\right)^{-1}(\boldsymbol{\xi}_{\mathcal{A}}^{(m)} + \boldsymbol{\eta}_{\mathcal{A}}^{(m)})\right\|_\infty + \left\|\boldsymbol{\xi}_{\mathcal{A}^c}^{(m)} + \boldsymbol{\eta}_{\mathcal{A}^c}^{(m)}\right\|_\infty\\ &\leq \left(\left\|\widetilde{\mathbf{V}}_{\mathcal{A}^c\mathcal{A}}^{\mathcal{G}(m)}\left(\widetilde{\mathbf{V}}_{\mathcal{A}\mathcal{A}}^{\mathcal{G}(m)}\right)^{-1}\right\|_\infty + 1\right)\left(\left\|\boldsymbol{\xi}^{(m)}\right\|_\infty + \left\|\boldsymbol{\eta}^{(m)}\right\|_\infty\right).\end{aligned} \tag{A.22}$$

Following Xue et al. (2012), we can derive the upper bound of $\|\widetilde{\mathbf{V}}_{\mathcal{A}^c\mathcal{A}}^{\mathcal{G}(m)}\left(\widetilde{\mathbf{V}}_{\mathcal{A}\mathcal{A}}^{\mathcal{G}(m)}\right)^{-1}\|_\infty, m \in [M]$. Following the definition of $\widetilde{\mathbf{V}}^{\mathcal{G}(m)}(m = 1, \ldots, M)$, we can also define $\mathbf{V}^{*\mathcal{G}(m)}(m = 1, \ldots, M)$ accordingly. By Condition (C3), we define

$$c_m := \left\|\left[\mathbb{E}(\mathbf{V}_{\mathcal{A}\mathcal{A}}^{*\mathcal{G}(m)})\right]^{-1}\right\|_\infty \leq \sqrt{q}\left\|\left[\mathbb{E}(\mathbf{V}_{\mathcal{A}\mathcal{A}}^{*\mathcal{G}(m)})\right]^{-1}\right\|_2 \leq \frac{\sqrt{q}K}{|\mathcal{G}_{\min}|C_{\min}}. \tag{A.23}$$

Furthermore, we define

$$\phi_m = \left\|\widetilde{\mathbf{V}}^{\mathcal{G}(m)}_{\mathcal{A}^c\mathcal{A}}\Big(\widetilde{\mathbf{V}}^{\mathcal{G}(m)}_{\mathcal{A}\mathcal{A}}\Big)^{-1} - \mathbb{E}\big(\mathbf{V}^{*\mathcal{G}(m)}_{\mathcal{A}^c\mathcal{A}}\big)\big[\mathbb{E}\big(\mathbf{V}^{*\mathcal{G}(m)}_{\mathcal{A}\mathcal{A}}\big)\big]^{-1}\right\|_\infty,$$
$$\phi_{1m} = \left\|\big(\widetilde{\mathbf{V}}^{\mathcal{G}(m)}_{\mathcal{A}\mathcal{A}}\big)^{-1} - \big[\mathbb{E}\big(\mathbf{V}^{*\mathcal{G}(m)}_{\mathcal{A}\mathcal{A}}\big)\big]^{-1}\right\|_\infty,$$
$$\phi_{2m} = \left\|\widetilde{\mathbf{V}}^{\mathcal{G}(m)}_{\mathcal{A}\mathcal{A}} - \mathbb{E}\big(\mathbf{V}^{*\mathcal{G}(m)}_{\mathcal{A}\mathcal{A}}\big)\right\|_\infty, \qquad \phi_{3m} = \left\|\widetilde{\mathbf{V}}^{\mathcal{G}(m)}_{\mathcal{A}^c\mathcal{A}} - \mathbb{E}\big(\mathbf{V}^{*\mathcal{G}(m)}_{\mathcal{A}^c\mathcal{A}}\big)\right\|_\infty.$$

Then by definition,

$$\begin{aligned}\phi_m = \Big\|&\Big[\widetilde{\mathbf{V}}^{\mathcal{G}(m)}_{\mathcal{A}^c\mathcal{A}} - \mathbb{E}\big(\mathbf{V}^{*\mathcal{G}(m)}_{\mathcal{A}^c\mathcal{A}}\big)\Big]\Big[\big(\widetilde{\mathbf{V}}^{\mathcal{G}(m)}_{\mathcal{A}\mathcal{A}}\big)^{-1} - \big[\mathbb{E}\big(\mathbf{V}^{*\mathcal{G}(m)}_{\mathcal{A}\mathcal{A}}\big)\big]^{-1}\Big]\\ &+ \mathbb{E}\big(\mathbf{V}^{*\mathcal{G}(m)}_{\mathcal{A}^c\mathcal{A}}\big)\big[\mathbb{E}\big(\mathbf{V}^{*\mathcal{G}(m)}_{\mathcal{A}\mathcal{A}}\big)\big]^{-1}\Big[-\widetilde{\mathbf{V}}^{\mathcal{G}(m)}_{\mathcal{A}\mathcal{A}} + \mathbb{E}\big(\mathbf{V}^{*\mathcal{G}(m)}_{\mathcal{A}\mathcal{A}}\big)\Big]\big(\widetilde{\mathbf{V}}^{\mathcal{G}(m)}_{\mathcal{A}\mathcal{A}}\big)^{-1}\\ &+ \Big[\widetilde{\mathbf{V}}^{\mathcal{G}(m)}_{\mathcal{A}^c\mathcal{A}} - \mathbb{E}\big(\mathbf{V}^{*\mathcal{G}(m)}_{\mathcal{A}^c\mathcal{A}}\big)\Big]\big[\mathbb{E}\big(\mathbf{V}^{*\mathcal{G}(m)}_{\mathcal{A}\mathcal{A}}\big)\big]^{-1}\Big\|_\infty\\ \le\ &\phi_{3m}\phi_{1m} + \varphi^{\mathcal{G}(m)}\phi_{2m}\left\|\big(\widetilde{\mathbf{V}}^{\mathcal{G}(m)}_{\mathcal{A}\mathcal{A}}\big)^{-1}\right\|_\infty + \phi_{3m}c_m\\ \le\ &\phi_{3m}\phi_{1m} + \varphi^{\mathcal{G}(m)}\phi_{2m}(c_m + \phi_{1m}) + \phi_{3m}c_m.\end{aligned}$$

Besides, ϕ_{1m} can be reformulated as

$$\begin{aligned}\phi_{1m} =\ &\left\|\big(\widetilde{\mathbf{V}}^{\mathcal{G}(m)}_{\mathcal{A}\mathcal{A}}\big)^{-1}\Big[\mathbb{E}\big(\mathbf{V}^{*\mathcal{G}(m)}_{\mathcal{A}\mathcal{A}}\big) - \widetilde{\mathbf{V}}^{\mathcal{G}(m)}_{\mathcal{A}\mathcal{A}}\Big]\big[\mathbb{E}\big(\mathbf{V}^{*\mathcal{G}(m)}_{\mathcal{A}\mathcal{A}}\big)\big]^{-1}\right\|_\infty\\ \le\ &\left\|\big(\widetilde{\mathbf{V}}^{\mathcal{G}(m)}_{\mathcal{A}\mathcal{A}}\big)^{-1}\right\|_\infty\cdot\left\|\mathbb{E}\big(\mathbf{V}^{*\mathcal{G}(m)}_{\mathcal{A}\mathcal{A}}\big) - \widetilde{\mathbf{V}}^{\mathcal{G}(m)}_{\mathcal{A}\mathcal{A}}\right\|_\infty\cdot\left\|\big[\mathbb{E}\big(\mathbf{V}^{*\mathcal{G}(m)}_{\mathcal{A}\mathcal{A}}\big)\big]^{-1}\right\|_\infty\\ \le\ &(c_m + \phi_{1m})\phi_{2m}c_m.\end{aligned}$$

Thus, as long as $\phi_{2m}c_m < 1$, we have $\phi_{1m} \le \phi_{2m}c_m^2/(1 - \phi_{2m}c_m)$, which yields

$$\phi_m \le (\phi_{3m} + \varphi^{\mathcal{G}(m)}\phi_{2m})\frac{c_m}{1 - \phi_{2m}c_m}. \tag{A.24}$$

Then, by Lemma 2,

$$\begin{aligned}\phi_{2m} = \left\|\widetilde{\mathbf{V}}^{\mathcal{G}(m)}_{\mathcal{A}\mathcal{A}} - \mathbb{E}\big(\mathbf{V}^{*\mathcal{G}(m)}_{\mathcal{A}\mathcal{A}}\big)\right\|_\infty &\le \frac{q\sum_{k\in\mathcal{G}^{(m)}} n_k\left\|\widetilde{\mathbf{V}}^{(k)}_{\mathcal{A}\mathcal{A}} - \mathbb{E}\big(\mathbf{V}^{*(k)}_{\mathcal{A}\mathcal{A}}\big)\right\|_{\max}}{N}\\ &= O_p\left(\frac{|\mathcal{G}_{\max}|}{K}\cdot\sqrt{\frac{q^3\log p}{n^*}}\right).\end{aligned} \tag{A.25}$$

Combining (A.23), (A.25), and Condition (C7), we have

$$\phi_{2m}c_m = O_p\left(\frac{|\mathcal{G}_{\max}|}{|\mathcal{G}_{\min}|}\cdot\sqrt{\frac{q^4\log p}{n^*}}\right) = o_p(1). \tag{A.26}$$

Following the proof of (A.26), we also have

$$\phi_{3m}c_m = O_p\left(\frac{|\mathcal{G}_{\max}|}{|\mathcal{G}_{\min}|}\cdot\sqrt{\frac{q^4\log p}{n^*}}\right) = o_p(1). \tag{A.27}$$

Combining (A.24), (A.26) and (A.27), with probability approaching 1, for all $m \in [M]$ $\phi_m = o_p(\varphi_{\max})$,

$$\max_{m\in[M]} \left\| \widetilde{\mathbf{V}}^{\mathcal{G}(m)}_{\mathcal{A}^c\mathcal{A}} \left(\widetilde{\mathbf{V}}^{\mathcal{G}(m)}_{\mathcal{A}\mathcal{A}} \right)^{-1} \right\|_\infty \le \max_{m\in[M]} \phi_m + \max_{m\in[M]} \left\| \mathbb{E}\big(\mathbf{V}^{*\mathcal{G}(m)}_{\mathcal{A}^c\mathcal{A}}\big) \left[\mathbb{E}\big(\mathbf{V}^{*\mathcal{G}(m)}_{\mathcal{A}\mathcal{A}}\big)\right]^{-1} \right\|_\infty \le 2\varphi_{\max}. \tag{A.28}$$

Now we consider $\|\boldsymbol{\xi}^{(m)}\|_\infty, m \in [M]$. By Conditions (C1), (C2), Lemma 1, and the union bound

$$P\left(\max_{m\in[M]} \max_{j\in[p]} \left| \sum_{k\in\mathcal{G}^{(m)}} n_k \mathbf{g}_j^{*(k)} \right| \ge t\sqrt{N_{\max}\log p} \right) \le 2pM \exp\left(-\frac{C_1 t^2 \log p}{C_x^2 \kappa_x^2} \right) \le 2p^{2-C_3},$$

where $C_3 = C_1 t^2/(C_x^2\kappa_x^2)$. When t is sufficiently large and $2 - C_3 < 0$, $2p^{2-C_3} \to 0$ when $p \to \infty$. Hence,

$$\max_{m\in[M]} \left\| \boldsymbol{\xi}^{(m)} \right\|_\infty = O_p\left(\sqrt{\frac{|\mathcal{G}_{\max}| \log p}{KN}} \right). \tag{A.29}$$

Following the proof of (A.14), we have

$$\max_{m\in[M]} \left\| \boldsymbol{\eta}^{(m)} \right\|_\infty = O_p\left(\frac{|\mathcal{G}_{\max}| q \log p}{N} \right). \tag{A.30}$$

Combining (A.22), (A.28), (A.29), (A.30), since $\varphi_{\max} r_{2N} \ll \lambda_1$, where

$$r_{2N} = \sqrt{\frac{(|\mathcal{G}_{\max}|/|\mathcal{G}_{\min}|) M \log p}{KN}} + \frac{(|\mathcal{G}_{\max}|/|\mathcal{G}_{\min}|^{1/2}) M^{1/2} q \log p}{N},$$

we have verified that (A.20) is satisfied with probability approaching 1. Therefore, the KKT conditions have been verified, and the proof of Step 2 is completed. □

A.3 Proof of Theorem 2

Define

$$\mathcal{Q}(\boldsymbol{\theta}) = \underbrace{\mathcal{L}_1(\boldsymbol{\theta}) + \mathcal{P}_1(\boldsymbol{\theta})}_{\mathcal{L}(\boldsymbol{\theta})} + \mathcal{P}_2(\boldsymbol{\theta}), \qquad \mathcal{Q}^{\mathcal{G}}(\boldsymbol{\psi}) = \underbrace{\mathcal{L}_1^{\mathcal{G}}(\boldsymbol{\psi}) + \mathcal{P}_1^{\mathcal{G}}(\boldsymbol{\psi})}_{\mathcal{L}^{\mathcal{G}}(\boldsymbol{\psi})} + \mathcal{P}_2^{\mathcal{G}}(\boldsymbol{\psi}),$$

where

$$\mathcal{L}_1(\boldsymbol{\theta}) = \frac{1}{N}\sum_{k=1}^{K} n_k\Big(\boldsymbol{\theta}^{(k)\top}\widetilde{\mathbf{V}}^{(k)}\boldsymbol{\theta}^{(k)} - 2\boldsymbol{\theta}^{(k)\top}\widetilde{\boldsymbol{\zeta}}^{(k)}\Big),$$

$$\mathcal{P}_1(\boldsymbol{\theta}) = \sum_{j=2}^{p} p_\tau\big(\|\boldsymbol{\theta}_j\|_2, \lambda_1\big),$$

$$\mathcal{P}_2(\boldsymbol{\theta}) = \sum_{k<k'} p_\tau\left(\left\|\boldsymbol{\theta}^{(k)} - \boldsymbol{\theta}^{(k')}\right\|_2, \lambda_2\right),$$

$$\mathcal{L}_1^{\mathcal{G}}(\boldsymbol{\psi}) = \frac{1}{N}\sum_{m=1}^{M}\left[\boldsymbol{\psi}^{(m)\top}\left(\sum_{k\in\mathcal{G}^{(m)}} n_k\widetilde{\mathbf{V}}^{(k)}\right)\boldsymbol{\psi}^{(m)} - 2\boldsymbol{\psi}^{(m)\top}\left(\sum_{k\in\mathcal{G}^{(m)}} n_k\widetilde{\boldsymbol{\zeta}}^{(k)}\right)\right],$$

$$\mathcal{P}_1^{\mathcal{G}}(\boldsymbol{\psi}) = \sum_{j=2}^{p} p_\tau\left(\left[\sum_{m=1}^{M}|\mathcal{G}^{(m)}|\psi_j^{(m)2}\right]^{1/2}, \lambda_1\right),$$

$$\mathcal{P}_2^{\mathcal{G}}(\boldsymbol{\psi}) = \sum_{m<m'}\big|\mathcal{G}^{(m)}\big|\big|\mathcal{G}^{(m')}\big| p_\tau\left(\left\|\boldsymbol{\psi}^{(m)} - \boldsymbol{\psi}^{(m')}\right\|_2, \lambda_2\right).$$

Define two mapping functions $\mathcal{T}^{\mathcal{G}}(\cdot)$ and $\mathcal{T}(\cdot)$. Specifically, let $\mathcal{T}^{\mathcal{G}} : \mathcal{M}_{\mathcal{G}} \to \mathbb{R}^{p\times M}$ be the function such that $\mathcal{T}^{\mathcal{G}}(\boldsymbol{\theta})$ is the $p \times M$ matrix whose mth column equals the common coefficient vector of $\boldsymbol{\theta}^{(k)}$ for $k \in \mathcal{G}^{(m)}$. Additionally, let $\mathcal{T} : \mathbb{R}^{p\times K} \to \mathbb{R}^{p\times M}$ be the function such that $\mathcal{T}(\boldsymbol{\theta}) = \big\{|\mathcal{G}^{(m)}|^{-1}\sum_{k\in\mathcal{G}^{(m)}}\boldsymbol{\theta}^{(k)}\big\}_{m=1}^{M}$. Obviously, if $\boldsymbol{\theta} \in \mathcal{M}_{\mathcal{G}}$, $\mathcal{T}^{\mathcal{G}}(\boldsymbol{\theta}) = \mathcal{T}(\boldsymbol{\theta})$.

For every $\boldsymbol{\theta} \in \mathcal{M}_{\mathcal{G}}$, we have $\mathcal{P}_2(\boldsymbol{\theta}) = \mathcal{P}_2^{\mathcal{G}}(\mathcal{T}^{\mathcal{G}}(\boldsymbol{\theta}))$. Similarly, for every $\boldsymbol{\psi} \in \mathbb{R}^{p\times M}$, we have $\mathcal{P}_2(\mathcal{T}^{\mathcal{G}^{-1}}(\boldsymbol{\psi})) = \mathcal{P}_2^{\mathcal{G}}(\boldsymbol{\psi})$. Hence,

$$\mathcal{Q}(\boldsymbol{\theta}) = \mathcal{Q}^{\mathcal{G}}(\mathcal{T}^{\mathcal{G}}(\boldsymbol{\theta})), \qquad \mathcal{Q}^{\mathcal{G}}(\boldsymbol{\psi}) = \mathcal{Q}(\mathcal{T}^{\mathcal{G}^{-1}}(\boldsymbol{\psi})). \tag{A.31}$$

Consider the neighborhood of $\boldsymbol{\theta}^*$, denoted by Θ, which is defined as

$$\Theta_1 = \left\{\boldsymbol{\theta} \in \mathbb{R}^{p\times K} : \|\boldsymbol{\theta} - \boldsymbol{\theta}^*\|_F \le C|\mathcal{G}_{\max}|^{1/2} r_{1N}\right\}.$$

Define the event

$$E_1 = \left\{\|\widehat{\boldsymbol{\theta}}^{or} - \boldsymbol{\theta}^*\|_F \le C|\mathcal{G}_{\max}|^{1/2} r_{1N}\right\}.$$

Then, by the result in Theorem 1, for any $\epsilon > 0$, there exists a constant $C_\epsilon > 0$ such that for any $C \ge C_\epsilon$, $P(E_1) \ge 1 - \epsilon$. Accordingly, $\widehat{\boldsymbol{\theta}}^{or} \in \Theta_1$ with probability at least $1 - \epsilon$. Furthermore, we define another neighborhood

$$\Theta_2 = \left\{\boldsymbol{\theta} \in \mathbb{R}^{p\times K} : \|\boldsymbol{\theta} - \widehat{\boldsymbol{\theta}}^{or}\|_F \le t_N\right\},$$

where t_N is a positive sequence. For any $\boldsymbol{\theta} = (\boldsymbol{\theta}_{\mathcal{A}}^\top, \boldsymbol{\theta}_{\mathcal{A}^c}^\top)^\top \in \Theta_1$, let $\breve{\boldsymbol{\theta}} = (\boldsymbol{\theta}_{\mathcal{A}}^\top, \mathbf{0}_{(p-q)\times K}^\top)^\top$ and $\breve{\boldsymbol{\theta}}^{\mathcal{G}} = \mathcal{T}^{\mathcal{G}^{-1}}(\mathcal{T}(\breve{\boldsymbol{\theta}}))$. Then we show that $\widehat{\boldsymbol{\theta}}^{or}$ is a strictly local minimizer of objective function (3) with probability approaching 1 through the following two steps

(a) On event E_1, $\mathcal{Q}(\breve{\boldsymbol{\theta}}^{\mathcal{G}}) > \mathcal{Q}(\widehat{\boldsymbol{\theta}}^{or})$ for any $\boldsymbol{\theta} \in \Theta_1$ and $\boldsymbol{\theta}^{\mathcal{G}} \ne \widehat{\boldsymbol{\theta}}^{or}$;

(b) On event E_1, $\mathcal{Q}(\boldsymbol{\theta}) \geq \mathcal{Q}(\breve{\boldsymbol{\theta}}) \geq \mathcal{Q}(\breve{\boldsymbol{\theta}}^{\mathcal{G}})$ for any $\boldsymbol{\theta} \in \Theta_1 \cap \Theta_2$ for a sufficiently large N.

For any $\boldsymbol{\theta} \in \Theta_1$, let $\mathcal{T}(\breve{\boldsymbol{\theta}}) = (\breve{\boldsymbol{\psi}}^{(1)}, \ldots, \breve{\boldsymbol{\psi}}^{(M)})$. Note that,

$$\mathcal{P}_2^{\mathcal{G}}(\mathcal{T}(\breve{\boldsymbol{\theta}})) = \sum_{m<m'} |\mathcal{G}^{(m)}||\mathcal{G}^{(m')}| p_\tau \left(\left\| \breve{\boldsymbol{\psi}}^{(m)} - \breve{\boldsymbol{\psi}}^{(m')} \right\|_2, \lambda_2 \right),$$

and, for any $m, m' \in [M]$ and $m \neq m'$, we have

$$\begin{aligned}
\left\| \breve{\boldsymbol{\psi}}^{(m)} - \breve{\boldsymbol{\psi}}^{(m')} \right\|_2 &\geq \min_{m,m' \in [M], m \neq m'} \left\| \boldsymbol{\psi}^{*(m)} - \boldsymbol{\psi}^{*(m')} \right\|_2 - 2 \cdot \max_{m \in [M]} \left\| \breve{\boldsymbol{\psi}}^{(m)} - \boldsymbol{\psi}^{*(m)} \right\|_2 \\
&\geq d_2 - 2 \cdot \max_{m \in [M]} \left\| |\mathcal{G}^{(m)}|^{-1} \sum_{k \in \mathcal{G}^{(m)}} (\breve{\boldsymbol{\theta}}^{(k)} - \boldsymbol{\theta}^{*(k)}) \right\|_2 \\
&\geq d_2 - 2 \cdot \max_{k \in [K]} \left\| \breve{\boldsymbol{\theta}}^{(k)} - \boldsymbol{\theta}^{*(k)} \right\|_2 \\
&\geq d_2 - 2C|\mathcal{G}_{\max}|^{1/2} r_{1N} \\
&> \tau \lambda_2,
\end{aligned} \tag{A.32}$$

where C is a constant and the last inequality follows from $d_2 > \tau\lambda_2$ and $\lambda_2 \gg |\mathcal{G}_{\max}|^{1/2} r_{1N}$. Consequently, for any $\boldsymbol{\theta} \in \Theta_1$, $\mathcal{P}_2^{\mathcal{G}}(\mathcal{T}(\breve{\boldsymbol{\theta}})) = C_N$, and $C_N > 0$ is a constant. By the result of Theorem 1, $\widehat{\boldsymbol{\psi}}^{or}$ is the local minimizer of objective function $\mathcal{L}^{\mathcal{G}}(\boldsymbol{\psi})$ with probability approaching 1. Thus we have $\mathcal{L}^{\mathcal{G}}(\widehat{\boldsymbol{\psi}}^{or}) < \mathcal{L}^{\mathcal{G}}(\mathcal{T}(\breve{\boldsymbol{\theta}}))$ for any $\boldsymbol{\theta} \in \Theta_1$ and $\mathcal{T}(\breve{\boldsymbol{\theta}}) \neq \widehat{\boldsymbol{\psi}}^{or}$. Combining this with $\mathcal{P}_2^{\mathcal{G}}(\mathcal{T}(\breve{\boldsymbol{\theta}})) = C_N$ for $\boldsymbol{\theta} \in \Theta_1$, we have $\mathcal{Q}^{\mathcal{G}}(\widehat{\boldsymbol{\psi}}^{or}) < \mathcal{Q}^{\mathcal{G}}(\mathcal{T}(\breve{\boldsymbol{\theta}}))$. By (A.31), we have

$$\mathcal{Q}^{\mathcal{G}}(\widehat{\boldsymbol{\psi}}^{or}) = \mathcal{Q}(\mathcal{T}^{\mathcal{G}^{-1}}(\widehat{\boldsymbol{\psi}}^{or})) = \mathcal{Q}(\widehat{\boldsymbol{\theta}}^{or}), \qquad \mathcal{Q}^{\mathcal{G}}(\mathcal{T}(\breve{\boldsymbol{\theta}})) = \mathcal{Q}(\mathcal{T}^{\mathcal{G}^{-1}}(\mathcal{T}(\breve{\boldsymbol{\theta}}))) = \mathcal{Q}(\breve{\boldsymbol{\theta}}^{\mathcal{G}}).$$

Accordingly, we have $\mathcal{Q}(\widehat{\boldsymbol{\theta}}^{or}) < \mathcal{Q}(\breve{\boldsymbol{\theta}}^{\mathcal{G}})$ for any $\boldsymbol{\theta} \in \Theta_1$ and $\breve{\boldsymbol{\theta}}^{\mathcal{G}} \neq \widehat{\boldsymbol{\theta}}^{or}$. This finishes the proof of the result in (a).

Next, we show that the result in (b) holds with probability approaching 1. First, we show that, on event E_1, $\mathcal{Q}(\boldsymbol{\theta}) \geq \mathcal{Q}(\breve{\boldsymbol{\theta}})$ for any $\boldsymbol{\theta} \in \Theta_1 \cap \Theta_2$. By the Taylor series expansion, we have

$$\mathcal{Q}(\boldsymbol{\theta}) - \mathcal{Q}(\breve{\boldsymbol{\theta}}) = \boldsymbol{\Omega}_{11} + \boldsymbol{\Omega}_{12} + \boldsymbol{\Omega}_{13}, \tag{A.33}$$

where

$$\begin{aligned}
\boldsymbol{\Omega}_{11} &= \sum_{j=1}^{p} \frac{\partial \mathcal{L}_1(\overline{\boldsymbol{\theta}})}{\partial \boldsymbol{\theta}_j^\top} (\boldsymbol{\theta}_j - \breve{\boldsymbol{\theta}}_j), \qquad \boldsymbol{\Omega}_{12} = \sum_{j=2}^{p} \frac{\partial \mathcal{P}_1(\overline{\boldsymbol{\theta}})}{\partial \boldsymbol{\theta}_j^\top} (\boldsymbol{\theta}_j - \breve{\boldsymbol{\theta}}_j), \\
\boldsymbol{\Omega}_{13} &= \sum_{k<k'} \left[p_\tau \left(\left\| \boldsymbol{\theta}^{(k)} - \boldsymbol{\theta}^{(k')} \right\|_2, \lambda_2 \right) - p_\tau \left(\left\| \breve{\boldsymbol{\theta}}^{(k)} - \breve{\boldsymbol{\theta}}^{(k')} \right\|_2, \lambda_2 \right) \right],
\end{aligned}$$

in which $\overline{\boldsymbol{\theta}} = \delta_1 \boldsymbol{\theta} + (1 - \delta_1) \breve{\boldsymbol{\theta}}$ for some $\delta_1 \in (0, 1)$. Note that, for any $j \in \mathcal{A}$, $\boldsymbol{\theta}_j = \breve{\boldsymbol{\theta}}_j$, and for any $j \in \mathcal{A}^c$, $\breve{\boldsymbol{\theta}}_j = \mathbf{0}$ and $\overline{\boldsymbol{\theta}}_j = \delta_1 \boldsymbol{\theta}_j$. Since $p_\tau(t, \lambda_2)$ is a nondecreasing function of t with

$t \in [0, \infty)$, $\mathbf{\Omega}_{13} \geq 0$. Besides,

$$\begin{aligned}\mathbf{\Omega}_{12} = & \sum_{j \in \mathcal{A}^c} p'_\tau \left(\left\|\overline{\boldsymbol{\theta}}_j\right\|_2, \lambda_1\right) \frac{\overline{\boldsymbol{\theta}}_j^\top (\boldsymbol{\theta}_j - \breve{\boldsymbol{\theta}}_j)}{\left\|\overline{\boldsymbol{\theta}}_j\right\|_2} \\ = & \sum_{j \in \mathcal{A}^c} p'_\tau(\delta_1 \|\boldsymbol{\theta}_j\|_2, \lambda_1) \|\boldsymbol{\theta}_j\|_2 \\ \geq & \sum_{j \in \mathcal{A}^c} p'_\tau(t_N, \lambda_1) \|\boldsymbol{\theta}_j\|_2,\end{aligned} \tag{A.34}$$

where the last inequality follows from the concavity of $p_\tau(t, \lambda_1)$. Furthermore,

$$\begin{aligned}|\mathbf{\Omega}_{11}| & = \left| \sum_{j \in \mathcal{A}^c} \frac{\partial \mathcal{L}_1(\overline{\boldsymbol{\theta}})}{\partial \boldsymbol{\theta}_j^\top} (\boldsymbol{\theta}_j - \breve{\boldsymbol{\theta}}_j) \right| \\ & \leq \sum_{j \in \mathcal{A}^c} \left\| \frac{\partial \mathcal{L}_1(\overline{\boldsymbol{\theta}})}{\partial \boldsymbol{\theta}_j^\top} \right\|_2 \|\boldsymbol{\theta}_j\|_2 \\ & \leq \sqrt{K} \max_{k \in [K]} \left\| \frac{\partial \mathcal{L}_1(\overline{\boldsymbol{\theta}})}{\partial \boldsymbol{\theta}_{\mathcal{A}^c}^{(k)}} \right\|_\infty \sum_{j \in \mathcal{A}^c} \|\boldsymbol{\theta}_j\|_2.\end{aligned} \tag{A.35}$$

Combining (A.33), (A.34), and (A.35), we have

$$\begin{aligned}\mathcal{Q}(\boldsymbol{\theta}) - \mathcal{Q}(\breve{\boldsymbol{\theta}}) & = \mathbf{\Omega}_{11} + \mathbf{\Omega}_{12} + \mathbf{\Omega}_{13} \\ & \geq \sum_{j \in \mathcal{A}^c} \left[p'_\tau(t_N, \lambda_1) - \sqrt{K} \max_{k \in [K]} \left\| \partial \mathcal{L}_1(\overline{\boldsymbol{\theta}}) / \partial \boldsymbol{\theta}_{\mathcal{A}^c}^{(k)} \right\|_\infty \right] \|\boldsymbol{\theta}_j\|_2.\end{aligned} \tag{A.36}$$

Following the proof from (A.21) to (A.30), we have

$$\sqrt{K} \max_{k \in [K]} \left\| \partial \mathcal{L}_1(\overline{\boldsymbol{\theta}}) / \partial \boldsymbol{\theta}_{\mathcal{A}^c}^{(k)} \right\|_\infty = O_p\left(\varphi_{\max}\left[\sqrt{\log p / N} + K^{1/2} q \log p / N \right] + (Kq)^{1/2} t_N \right).$$

In addition, let $t_N = o(1)$, and then $p'_\tau(t_N, \lambda_1) \to \lambda_1$. Furthermore, let $(Kq)^{1/2} t_N \ll \lambda_1$, and then

$$\lambda_1 \gg \varphi_{\max}\left[r_{2N} + \sqrt{\log p / N} \right]$$

leads to

$$\lambda_1 \gg \varphi_{\max}\left[\sqrt{\log p / N} + K^{1/2} q \log p / N \right] + (Kq)^{1/2} t_N. \tag{A.37}$$

Then, by (A.36) and (A.37), when N is sufficiently large, with probability approaching 1,

$$\mathcal{Q}(\boldsymbol{\theta}) - \mathcal{Q}(\breve{\boldsymbol{\theta}}) \geq 0.$$

Next, we show that, on event E_1, $\mathcal{Q}(\breve{\boldsymbol{\theta}}) \geq Q(\breve{\boldsymbol{\theta}}^{\mathcal{G}})$ for any $\boldsymbol{\theta} \in \Theta_1 \cap \Theta_2$. By the Taylor series expansion, we have

$$\mathcal{Q}(\breve{\boldsymbol{\theta}}) - \mathcal{Q}(\breve{\boldsymbol{\theta}}^{\mathcal{G}}) = \mathbf{\Omega}_{21} + \mathbf{\Omega}_{22} + \mathbf{\Omega}_{23},$$

where

$$\boldsymbol{\Omega}_{21} = \sum_{k=1}^{K} \frac{\partial \mathcal{L}_1(\breve{\boldsymbol{\theta}}^{\hbar})}{\partial \boldsymbol{\theta}^{(k)\top}} (\breve{\boldsymbol{\theta}}^{(k)} - \breve{\boldsymbol{\theta}}^{\mathcal{G}(k)}), \qquad \boldsymbol{\Omega}_{22} = \sum_{j=2}^{p} \frac{\partial \mathcal{P}_1(\breve{\boldsymbol{\theta}}^{\hbar})}{\partial \boldsymbol{\theta}_j^{\top}} (\breve{\boldsymbol{\theta}}_j - \breve{\boldsymbol{\theta}}_j^{\mathcal{G}}),$$

$$\boldsymbol{\Omega}_{23} = \sum_{k<k'} \frac{\partial \mathcal{P}_2(\breve{\boldsymbol{\theta}}^{\hbar})}{\partial \boldsymbol{\theta}^{(k)\top}} (\breve{\boldsymbol{\theta}}^{(k)} - \breve{\boldsymbol{\theta}}^{\mathcal{G}(k)}),$$

in which $\breve{\boldsymbol{\theta}}^{\hbar} = \delta_2 \breve{\boldsymbol{\theta}} + (1-\delta_2)\breve{\boldsymbol{\theta}}^{\mathcal{G}}$ for some $\delta_2 \in (0,1)$. Next, we bound $\boldsymbol{\Omega}_{21}$, $\boldsymbol{\Omega}_{22}$, and $\boldsymbol{\Omega}_{23}$. Recall that, for any $j \in \mathcal{A}^c$, $\breve{\boldsymbol{\theta}}^{\hbar}_{\mathcal{A}^c} = \breve{\boldsymbol{\theta}}_{\mathcal{A}^c} = \breve{\boldsymbol{\theta}}^{\mathcal{G}}_{\mathcal{A}^c} = \mathbf{0}$. Then,

$$\boldsymbol{\Omega}_{22} = \sum_{j=2}^{q} \frac{\partial \mathcal{P}_1(\breve{\boldsymbol{\theta}}^{\hbar})}{\partial \boldsymbol{\theta}_j^{\top}} (\breve{\boldsymbol{\theta}}_j - \breve{\boldsymbol{\theta}}_j^{\mathcal{G}}) = \sum_{j=2}^{q} p'_{\tau}\left(\left\|\breve{\boldsymbol{\theta}}_j^{\hbar}\right\|_2, \lambda_1\right) \frac{\breve{\boldsymbol{\theta}}_j^{\hbar\top}(\breve{\boldsymbol{\theta}}_j - \breve{\boldsymbol{\theta}}_j^{\mathcal{G}})}{||\breve{\boldsymbol{\theta}}_j^{\hbar}||_2}. \tag{A.38}$$

Note that,

$$\begin{aligned}
\left\|\breve{\boldsymbol{\theta}}_j^{\mathcal{G}} - \boldsymbol{\theta}_j^*\right\|_2 &= \sqrt{\sum_{m=1}^{M} |\mathcal{G}^{(m)}| \left(\frac{\sum_{k\in\mathcal{G}^{(m)}} (\breve{\theta}_j^{(k)} - \psi_j^{*(m)})}{|\mathcal{G}^{(m)}|} \right)^2} \\
&\le \sqrt{\sum_{m=1}^{M} |\mathcal{G}^{(m)}| \times \frac{|\mathcal{G}^{(m)}| \left[\sum_{k\in\mathcal{G}^{(m)}} \left(\breve{\theta}_j^{(k)} - \psi_j^{*(m)}\right)^2 \right]}{|\mathcal{G}^{(m)}|^2}} \\
&= \left\|\breve{\boldsymbol{\theta}}_j - \boldsymbol{\theta}_j^*\right\|_2.
\end{aligned}$$

Besides, since $\breve{\boldsymbol{\theta}}_j^{\hbar} = \delta_2 \breve{\boldsymbol{\theta}}_j + (1-\delta_2)\breve{\boldsymbol{\theta}}_j^{\mathcal{G}}$,

$$\left\|\breve{\boldsymbol{\theta}}_j^{\hbar} - \boldsymbol{\theta}_j^*\right\|_2 \le \delta_2 \left\|\breve{\boldsymbol{\theta}}_j - \boldsymbol{\theta}_j^*\right\|_2 + (1-\delta_2)\left\|\breve{\boldsymbol{\theta}}_j^{\mathcal{G}} - \boldsymbol{\theta}_j^*\right\|_2 \le \left\|\breve{\boldsymbol{\theta}}_j - \boldsymbol{\theta}_j^*\right\|_2.$$

Hence, for any $j \in \mathcal{A}$, by the triangle inequality,

$$\begin{aligned}
\left\|\breve{\boldsymbol{\theta}}_j^{\hbar}\right\|_2 &\ge \|\boldsymbol{\theta}_j^*\|_2 - \left\|\breve{\boldsymbol{\theta}}_j^{\hbar} - \boldsymbol{\theta}_j^*\right\|_2 \\
&\ge \min_{j\in\mathcal{A}} \|\boldsymbol{\theta}_j^*\|_2 - \max_{j\in\mathcal{A}} \left\|\breve{\boldsymbol{\theta}}_j - \boldsymbol{\theta}_j^*\right\|_2 \\
&\ge |\mathcal{G}_{\min}|^{1/2} d_1 - C|\mathcal{G}_{\max}|^{1/2} r_{1N} \\
&> \tau\lambda_1,
\end{aligned} \tag{A.39}$$

where the last inequality follows from $|\mathcal{G}_{\min}|^{1/2} d_1 > \tau\lambda_1 \gg |\mathcal{G}_{\max}|^{1/2} r_{1N}$. Combining (A.38) and (A.39), since $p_{\tau}(t, \lambda_1)$ is a constant for $t \ge \tau\lambda_1$, we have $\boldsymbol{\Omega}_{22} = 0$.

Now consider $\boldsymbol{\Omega}_{23}$. Recall that, for any $j \in \mathcal{A}^c$, $\breve{\boldsymbol{\theta}}^{\hbar}_{\mathcal{A}^c} = \breve{\boldsymbol{\theta}}_{\mathcal{A}^c} = \breve{\boldsymbol{\theta}}^{\mathcal{G}}_{\mathcal{A}^c} = \mathbf{0}$. Then,

$$\begin{aligned}
\boldsymbol{\Omega}_{23} = & \sum_{k<k'} \Bigg\{ p'_\tau \left(\left\| \breve{\boldsymbol{\theta}}^{\hbar(k)}_{\mathcal{A}} - \breve{\boldsymbol{\theta}}^{\hbar(k')}_{\mathcal{A}} \right\|_2, \lambda_2 \right) \left\| \breve{\boldsymbol{\theta}}^{\hbar(k)}_{\mathcal{A}} - \breve{\boldsymbol{\theta}}^{\hbar(k')}_{\mathcal{A}} \right\|_2^{-1} \\
& \qquad \times \left(\breve{\boldsymbol{\theta}}^{\hbar(k)}_{\mathcal{A}} - \breve{\boldsymbol{\theta}}^{\hbar(k')}_{\mathcal{A}} \right)^\top \left[(\breve{\boldsymbol{\theta}}^{(k)}_{\mathcal{A}} - \breve{\boldsymbol{\theta}}^{\mathcal{G}(k)}_{\mathcal{A}}) - (\breve{\boldsymbol{\theta}}^{(k')}_{\mathcal{A}} - \breve{\boldsymbol{\theta}}^{\mathcal{G}(k')}_{\mathcal{A}}) \right] \Bigg\} \\
= & \sum_{m=1}^{M} \sum_{k,k' \in \mathcal{G}^{(m)}, k<k'} p'_\tau \left(\left\| \breve{\boldsymbol{\theta}}^{\hbar(k)}_{\mathcal{A}} - \breve{\boldsymbol{\theta}}^{\hbar(k')}_{\mathcal{A}} \right\|_2, \lambda_2 \right) \left\| \breve{\boldsymbol{\theta}}^{(k)}_{\mathcal{A}} - \breve{\boldsymbol{\theta}}^{(k')}_{\mathcal{A}} \right\|_2 \\
& + \sum_{m<m'} \sum_{k \in \mathcal{G}^{(m)}, k' \in \mathcal{G}^{(m')}} \Bigg\{ p'_\tau \left(\left\| \breve{\boldsymbol{\theta}}^{\hbar(k)}_{\mathcal{A}} - \breve{\boldsymbol{\theta}}^{\hbar(k')}_{\mathcal{A}} \right\|_2, \lambda_2 \right) \left\| \breve{\boldsymbol{\theta}}^{\hbar(k)}_{\mathcal{A}} - \breve{\boldsymbol{\theta}}^{\hbar(k')}_{\mathcal{A}} \right\|_2^{-1} \\
& \qquad \times \left(\breve{\boldsymbol{\theta}}^{\hbar(k)}_{\mathcal{A}} - \breve{\boldsymbol{\theta}}^{\hbar(k')}_{\mathcal{A}} \right)^\top \left[(\breve{\boldsymbol{\theta}}^{(k)}_{\mathcal{A}} - \breve{\boldsymbol{\theta}}^{\mathcal{G}(k)}_{\mathcal{A}}) - (\breve{\boldsymbol{\theta}}^{(k')}_{\mathcal{A}} - \breve{\boldsymbol{\theta}}^{\mathcal{G}(k')}_{\mathcal{A}}) \right] \Bigg\},
\end{aligned} \tag{A.40}$$

where the first term of the second equality follows from the fact that, when $k, k' \in \mathcal{G}^{(m)}$, $\breve{\boldsymbol{\theta}}^{\mathcal{G}(k)}_{\mathcal{A}} = \breve{\boldsymbol{\theta}}^{\mathcal{G}(k')}_{\mathcal{A}}$ and $\breve{\boldsymbol{\theta}}^{\hbar(k)}_{\mathcal{A}} - \breve{\boldsymbol{\theta}}^{\hbar(k')}_{\mathcal{A}} = \delta_2(\breve{\boldsymbol{\theta}}^{(k)}_{\mathcal{A}} - \breve{\boldsymbol{\theta}}^{(k')}_{\mathcal{A}})$. Note that, for any $m \in [M]$, $k \in \mathcal{G}^{(m)}$,

$$\left\| \breve{\boldsymbol{\theta}}^{\mathcal{G}(k)}_{\mathcal{A}} - \boldsymbol{\theta}^{*(k)}_{\mathcal{A}} \right\|_2 = \left\| \frac{\sum_{k \in \mathcal{G}^{(m)}} \breve{\boldsymbol{\theta}}^{(k)}_{\mathcal{A}}}{|\mathcal{G}^{(m)}|} - \boldsymbol{\psi}^{*(m)}_{\mathcal{A}} \right\|_2 \le \max_{k \in [K]} \left\| \breve{\boldsymbol{\theta}}^{(k)}_{\mathcal{A}} - \boldsymbol{\theta}^{*(k)}_{\mathcal{A}} \right\|_2.$$

And then for any $k \in [K]$, we have $\|\breve{\boldsymbol{\theta}}^{\hbar(k)}_{\mathcal{A}} - \boldsymbol{\theta}^{*(k)}_{\mathcal{A}}\|_2 \le \max_{k \in [K]} \|\breve{\boldsymbol{\theta}}^{(k)}_{\mathcal{A}} - \boldsymbol{\theta}^{*(k)}_{\mathcal{A}}\|_2$. Hence, similar to (A.32), for any $m < m'$, $k \in \mathcal{G}^{(m)}$, $k' \in \mathcal{G}^{(m')}$,

$$\begin{aligned}
\left\| \breve{\boldsymbol{\theta}}^{\hbar(k)}_{\mathcal{A}} - \breve{\boldsymbol{\theta}}^{\hbar(k')}_{\mathcal{A}} \right\|_2 \ge & \min_{k \in \mathcal{G}^{(m)}, k' \in \mathcal{G}^{(m')}} \left\| \boldsymbol{\theta}^{*(k)}_{\mathcal{A}} - \boldsymbol{\theta}^{*(k')}_{\mathcal{A}} \right\|_2 - 2 \max_{k \in [K]} \left\| \breve{\boldsymbol{\theta}}^{\hbar(k)}_{\mathcal{A}} - \boldsymbol{\theta}^{*(k)}_{\mathcal{A}} \right\|_2 \\
\ge & \min_{k \in \mathcal{G}^{(m)}, k' \in \mathcal{G}^{(m')}} \left\| \boldsymbol{\theta}^{*(k)}_{\mathcal{A}} - \boldsymbol{\theta}^{*(k')}_{\mathcal{A}} \right\|_2 - 2 \max_{k \in [K]} \left\| \breve{\boldsymbol{\theta}}^{(k)}_{\mathcal{A}} - \boldsymbol{\theta}^{*(k)}_{\mathcal{A}} \right\|_2 \\
\ge & d_2 - 2C|\mathcal{G}_{\max}|^{1/2} r_{1N} > \tau \lambda_2.
\end{aligned} \tag{A.41}$$

Combining (A.40) and (A.41), since that

$$\left\| \breve{\boldsymbol{\theta}}^{\hbar(k)}_{\mathcal{A}} - \breve{\boldsymbol{\theta}}^{\hbar(k')}_{\mathcal{A}} \right\|_2 \le 2 \max_{k \in [K]} \left\| \breve{\boldsymbol{\theta}}^{(k)}_{\mathcal{A}} - \boldsymbol{\theta}^{*(k)}_{\mathcal{A}} \right\|_2 \le 2C|\mathcal{G}_{\max}|^{1/2} r_{1N},$$

we have

$$\begin{aligned}
\boldsymbol{\Omega}_{23} = & \sum_{m=1}^{M} \sum_{k,k' \in \mathcal{G}^{(m)}, k<k'} p'_\tau \left(\left\| \breve{\boldsymbol{\theta}}^{\hbar(k)}_{\mathcal{A}} - \breve{\boldsymbol{\theta}}^{\hbar(k')}_{\mathcal{A}} \right\|_2, \lambda_2 \right) \left\| \breve{\boldsymbol{\theta}}^{(k)}_{\mathcal{A}} - \breve{\boldsymbol{\theta}}^{(k')}_{\mathcal{A}} \right\|_2 \\
\ge & \sum_{m=1}^{M} \sum_{k,k' \in \mathcal{G}^{(m)}, k<k'} p'_\tau \Big(2C|\mathcal{G}_{\max}|^{1/2} r_N, \lambda_2 \Big) \left\| \breve{\boldsymbol{\theta}}^{(k)}_{\mathcal{A}} - \breve{\boldsymbol{\theta}}^{(k')}_{\mathcal{A}} \right\|_2 \\
\ge & \sum_{m=1}^{M} \sum_{k,k' \in \mathcal{G}^{(m)}, k<k'} \frac{\lambda_2}{2} \left\| \breve{\boldsymbol{\theta}}^{(k)}_{\mathcal{A}} - \breve{\boldsymbol{\theta}}^{(k')}_{\mathcal{A}} \right\|_2,
\end{aligned} \tag{A.42}$$

where the last inequality follows from $2C|\mathcal{G}_{\max}|^{1/2}r_{1N} \to 0$ when $N \to \infty$.

We now consider the bound of $\mathbf{\Omega}_{21}$. For $k \in [K]$, we define

$$\begin{aligned}
\boldsymbol{\omega}^{(k)} &:= \frac{\partial \mathcal{L}_1(\breve{\boldsymbol{\theta}}^{\hbar})}{\partial \boldsymbol{\theta}_{\mathcal{A}}^{(k)}} = 2\Big\{\big[(n_k/N)\widetilde{\mathbf{V}}_{\mathcal{A}\mathcal{A}}^{(k)}\big]\breve{\boldsymbol{\theta}}_{\mathcal{A}}^{\hbar(k)} - (n_k/N)\widetilde{\boldsymbol{\zeta}}_{\mathcal{A}}^{(k)}\Big\} \\
&= 2\Bigg\{\big[(n_k/N)\widetilde{\mathbf{V}}_{\mathcal{A}\mathcal{A}}^{(k)}\big](\breve{\boldsymbol{\theta}}_{\mathcal{A}}^{\hbar(k)} - \boldsymbol{\theta}_{\mathcal{A}}^{*(k)}) + (n_k/N)\mathbf{g}_{\mathcal{A}}^{*(k)} \\
&\qquad + (n_k/N)\int_0^1 \Big\{\mathbf{V}^{(k)}\Big(\big[\boldsymbol{\theta}^{*(k)} + t(\widetilde{\boldsymbol{\theta}}^{(k)} - \boldsymbol{\theta}^{*(k)})\big]\Big) - \widetilde{\mathbf{V}}^{(k)}\Big\}(\widetilde{\boldsymbol{\theta}}^{(k)} - \boldsymbol{\theta}^{*(k)})\, dt\Bigg\} \\
&:= \boldsymbol{\omega}_1^{(k)} + \boldsymbol{\omega}_2^{(k)} + \boldsymbol{\omega}_3^{(k)}.
\end{aligned}$$

Then,

$$\begin{aligned}
\mathbf{\Omega}_{21} &= \sum_{k=1}^{K} \boldsymbol{\omega}^{(k)\top}(\breve{\boldsymbol{\theta}}_{\mathcal{A}}^{(k)} - \breve{\boldsymbol{\theta}}_{\mathcal{A}}^{\mathcal{G}(k)}) = \sum_{m=1}^{M}\sum_{k,k'\in\mathcal{G}^{(m)}} \frac{\boldsymbol{\omega}^{(k)\top}(\breve{\boldsymbol{\theta}}_{\mathcal{A}}^{(k)} - \breve{\boldsymbol{\theta}}_{\mathcal{A}}^{(k')})}{|\mathcal{G}^{(m)}|} \\
&= \sum_{m=1}^{M}\sum_{k,k'\in\mathcal{G}^{(m)}} \frac{\boldsymbol{\omega}^{(k')\top}(\breve{\boldsymbol{\theta}}_{\mathcal{A}}^{(k')} - \breve{\boldsymbol{\theta}}_{\mathcal{A}}^{(k)})}{2|\mathcal{G}^{(m)}|} + \sum_{m=1}^{M}\sum_{k,k'\in\mathcal{G}^{(m)}} \frac{\boldsymbol{\omega}^{(k)\top}(\breve{\boldsymbol{\theta}}_{\mathcal{A}}^{(k)} - \breve{\boldsymbol{\theta}}_{\mathcal{A}}^{(k')})}{2|\mathcal{G}^{(m)}|} \\
&= \sum_{m=1}^{M}\sum_{k,k'\in\mathcal{G}^{(m)}} \frac{(\boldsymbol{\omega}^{(k)} - \boldsymbol{\omega}^{(k')})^\top(\breve{\boldsymbol{\theta}}_{\mathcal{A}}^{(k)} - \breve{\boldsymbol{\theta}}_{\mathcal{A}}^{(k')})}{2|\mathcal{G}^{(m)}|} \\
&= \sum_{m=1}^{M}\sum_{k,k'\in\mathcal{G}^{(m)},k<k'} \frac{(\boldsymbol{\omega}^{(k)} - \boldsymbol{\omega}^{(k')})^\top(\breve{\boldsymbol{\theta}}_{\mathcal{A}}^{(k)} - \breve{\boldsymbol{\theta}}_{\mathcal{A}}^{(k')})}{|\mathcal{G}^{(m)}|}.
\end{aligned}$$

Following the proof from (A.10) to (A.14) in Theorem 1, we can show that

$$\begin{aligned}
\max_{k\in[K]} \left\|\boldsymbol{\omega}_1^{(k)}\right\|_2 &= O_p\big(|\mathcal{G}_{\max}|^{1/2}r_{1N}/K\big), \\
\max_{k\in[K]} \left\|\boldsymbol{\omega}_2^{(k)}\right\|_2 &= O_p\big(\sqrt{q/(KN)}\big), \\
\max_{k\in[K]} \left\|\boldsymbol{\omega}_3^{(k)}\right\|_2 &= O_p\big(q^{3/2}\log p/N\big).
\end{aligned}$$

Then,

$$|\mathbf{\Omega}_{21}| \le \sum_{m=1}^{M}\sum_{k,k'\in\mathcal{G}^{(m)},k<k'} \frac{2\max_{k\in[K]}||\boldsymbol{\omega}^{(k)}||_2}{|\mathcal{G}_{\min}|}\left\|\breve{\boldsymbol{\theta}}_{\mathcal{A}}^{(k)} - \breve{\boldsymbol{\theta}}_{\mathcal{A}}^{(k')}\right\|_2. \tag{A.43}$$

Combining (A.42) and (A.43), since $\lambda_2 \gg |\mathcal{G}_{\max}|^{1/2}r_{1N}$, we have

$$\lambda_2 \gg \frac{|\mathcal{G}_{\max}|^{1/2}r_{1N}}{K|\mathcal{G}_{\min}|} + \sqrt{\frac{q}{KN|\mathcal{G}_{\min}|^2}} + \frac{q^{3/2}\log p}{N|\mathcal{G}_{\min}|},$$

which leads to

$$\mathcal{Q}(\breve{\boldsymbol{\theta}}) - \mathcal{Q}(\breve{\boldsymbol{\theta}}^{\mathcal{G}}) \ge \sum_{m=1}^{M}\sum_{k,k'\in\mathcal{G}^{(m)},k<k'} \left\{\frac{\lambda_2}{2} - \frac{2\max_{k\in[K]}\|\boldsymbol{\omega}^{(k)}\|_2}{|\mathcal{G}_{\min}|}\right\}\left\|\breve{\boldsymbol{\theta}}_{\mathcal{A}}^{(k)} - \breve{\boldsymbol{\theta}}_{\mathcal{A}}^{(k')}\right\|_2 \ge 0,$$

for a sufficiently large N with probability approaching 1. Thus, we have proved the result in (b). This finishes all the proofs of Theorem 2. □

Appendix B. Additional Numerical Results

This section contains simulation results for Examples 2–6 and additional data application results.

Table 4: The variable selection accuracy: mean (sd) based on 100 replicates in Example 2.

		$n = 200$			$n = 400$			$n = 800$		
	Method	TPR	FPR	MS	TPR	FPR	MS	TPR	FPR	MS
$K = 64$	ICR	**1.000**	**0.000**	**33.920**	**1.000**	**0.000**	**32.000**	**1.000**	**0.000**	**32.000**
		(0.000)	(0.000)	(3.959)	(0.000)	(0.000)	(0.000)	(0.000)	(0.000)	(0.000)
	IP	**1.000**	**0.000**	34.880	**1.000**	**0.000**	**32.000**	**1.000**	**0.000**	**32.000**
		(0.000)	(0.000)	(5.514)	(0.000)	(0.000)	(0.000)	(0.000)	(0.000)	(0.000)
	ICFL	0.903	0.099	65.280	0.896	0.016	34.680	0.900	0.002	29.360
		(0.193)	(0.062)	(27.846)	(0.203)	(0.024)	(12.777)	(0.201)	(0.006)	(7.083)
	OCFL	0.909	0.137	79.360	0.895	0.018	35.120	0.900	0.001	29.000
		(0.183)	(0.067)	(29.144)	(0.205)	(0.024)	(12.771)	(0.201)	(0.003)	(6.639)
	SHIR	0.760	0.001	389.220	**1.000**	0.001	512.700	**1.000**	0.004	533.770
		(0.042)	(0.004)	(21.718)	(0.000)	(0.003)	(6.400)	(0.000)	(0.005)	(30.463)
	SMA	0.763	0.001	390.500	**1.000**	0.001	513.330	**1.000**	0.003	528.000
		(0.042)	(0.004)	(21.367)	(0.000)	(0.003)	(9.002)	(0.000)	(0.005)	(27.852)
	Local	0.853	0.148	1309.750	0.987	0.198	1673.740	**1.000**	0.218	1794.750
		(0.020)	(0.010)	(63.847)	(0.005)	(0.010)	(56.069)	(0.001)	(0.011)	(63.438)
	SK(har)	0.945	0.526	222.880	0.996	0.934	375.480	**1.000**	0.967	387.880
		(0.098)	(0.456)	(169.339)	(0.028)	(0.137)	(51.331)	(0.000)	(0.020)	(7.335)
	SK(gap)	**1.000**	0.995	199.000	**1.000**	0.999	199.840	**1.000**	0.987	273.200
		(0.000)	(0.008)	(1.518)	(0.000)	(0.003)	(0.615)	(0.000)	(0.020)	(92.285)
	DLSA	0.125	**0.000**	1.030	0.125	**0.000**	1.000	0.125	**0.000**	1.000
		(0.000)	(0.002)	(0.171)	(0.000)	(0.000)	(0.000)	(0.000)	(0.000)	(0.000)
$K = 128$	ICR	**1.000**	**0.000**	**34.800**	**1.000**	**0.000**	32.080	**1.000**	**0.000**	**32.000**
		(0.000)	(0.000)	(5.005)	(0.000)	(0.000)	(0.800)	(0.000)	(0.000)	(0.000)
	IP	**1.000**	**0.000**	36.560	**1.000**	**0.000**	**32.000**	**1.000**	**0.000**	**32.000**
		(0.000)	(0.000)	(7.391)	(0.000)	(0.000)	(0.000)	(0.000)	(0.000)	(0.000)
	ICFL	0.915	0.033	41.240	0.940	0.005	31.760	0.890	**0.000**	28.600
		(0.189)	(0.046)	(19.750)	(0.163)	(0.016)	(8.372)	(0.208)	(0.002)	(6.760)
	OCFL	0.915	0.056	49.880	0.940	0.006	32.240	0.890	**0.000**	28.600
		(0.189)	(0.062)	(25.732)	(0.163)	(0.018)	(8.932)	(0.208)	(0.002)	(6.760)
	SHIR	0.784	**0.000**	802.560	**1.000**	0.005	1077.770	**1.000**	0.011	1152.000
		(0.068)	(0.000)	(70.120)	(0.000)	(0.005)	(63.485)	(0.000)	(0.000)	(0.000)
	SMA	0.781	**0.000**	800.000	**1.000**	0.004	1067.530	**1.000**	0.011	1152.000
		(0.074)	(0.000)	(75.835)	(0.000)	(0.005)	(60.954)	(0.000)	(0.000)	(0.000)
	Local	0.851	0.147	2602.260	0.987	0.199	3351.110	**1.000**	0.219	3599.230
		(0.015)	(0.007)	(94.466)	(0.003)	(0.007)	(84.503)	(0.000)	(0.007)	(85.526)
	SK(har)	0.899	0.505	216.400	**1.000**	0.988	394.320	**1.000**	0.980	392.440
		(0.121)	(0.497)	(189.078)	(0.000)	(0.100)	(38.757)	(0.000)	(0.141)	(52.333)
	SK(gap)	**1.000**	1.000	199.980	**1.000**	1.000	200.000	**1.000**	0.990	299.240
		(0.000)	(0.001)	(0.200)	(0.000)	(0.000)	(0.000)	(0.000)	(0.100)	(102.996)
	DLSA	0.125	**0.000**	1.000	0.125	**0.000**	1.000	0.125	**0.000**	1.000
		(0.000)	(0.000)	(0.000)	(0.000)	(0.000)	(0.000)	(0.000)	(0.000)	(0.000)

Table 5: The clustering accuracy: mean (sd) based on 100 replicates in Example 2.

		$n = 200$				$n = 400$				$n = 800$			
	Method	$\widehat{M}$	Per	RI	ARI	$\widehat{M}$	Per	RI	ARI	$\widehat{M}$	Per	RI	ARI
$K = 64$	ICR	4.240	0.790	**0.998**	**0.995**	**4.000**	**1.000**	**1.000**	**1.000**	**4.000**	**1.000**	**1.000**	**1.000**
		(0.495)	(-)	(0.004)	(0.010)	(0.000)	(-)	(0.000)	(0.000)	(0.000)	(-)	(0.000)	(0.000)
	IP	4.360	0.740	0.997	0.992	**4.000**	**1.000**	**1.000**	**1.000**	**4.000**	**1.000**	**1.000**	**1.000**
		(0.689)	(-)	(0.005)	(0.015)	(0.000)	(-)	(0.000)	(0.000)	(0.000)	(-)	(0.000)	(0.000)
	ICFL	**4.000**	**1.000**	0.944	0.865	**4.000**	**1.000**	0.951	0.881	**4.000**	**1.000**	0.941	0.858
		(0.000)	(-)	(0.075)	(0.181)	(0.000)	(-)	(0.072)	(0.174)	(0.000)	(-)	(0.076)	(0.183)
	OCFL	**4.000**	**1.000**	0.944	0.865	**4.000**	**1.000**	0.951	0.881	**4.000**	**1.000**	0.941	0.858
		(0.000)	(-)	(0.075)	(0.181)	(0.000)	(-)	(0.072)	(0.174)	(0.000)	(-)	(0.076)	(0.183)
	SK(har)	3.980	0.980	0.994	0.984	**4.000**	**1.000**	**1.000**	**1.000**	**4.000**	**1.000**	**1.000**	**1.000**
		(0.141)	(-)	(0.020)	(0.048)	(0.000)	(-)	(0.000)	(0.000)	(0.000)	(-)	(0.000)	(0.000)
	SK(gap)	2.000	0.000	0.746	0.487	2.000	0.000	0.746	0.488	2.780	0.390	0.845	0.688
		(0.000)	(-)	(0.002)	(0.003)	(0.000)	(-)	(0.000)	(0.000)	(0.980)	(-)	(0.124)	(0.251)
$K = 128$	ICR	4.350	0.640	0.991	0.978	4.010	0.990	**1.000**	**1.000**	**4.000**	**1.000**	**1.000**	**1.000**
		(0.626)	(-)	(0.030)	(0.070)	(0.100)	(-)	(0.000)	(0.001)	(0.000)	(-)	(0.000)	(0.000)
	IP	4.570	0.620	**0.998**	**0.994**	**4.000**	**1.000**	**1.000**	**1.000**	**4.000**	**1.000**	**1.000**	**1.000**
		(0.924)	(-)	(0.003)	(0.010)	(0.000)	(-)	(0.000)	(0.000)	(0.000)	(-)	(0.000)	(0.000)
	ICFL	**4.000**	**1.000**	0.944	0.866	**4.000**	**1.000**	0.952	0.885	**4.000**	**1.000**	0.935	0.844
		(0.000)	(-)	(0.075)	(0.179)	(0.000)	(-)	(0.072)	(0.172)	(0.000)	(-)	(0.077)	(0.184)
	OCFL	**4.000**	**1.000**	0.944	0.866	**4.000**	**1.000**	0.952	0.885	**4.000**	**1.000**	0.935	0.844
		(0.000)	(-)	(0.075)	(0.179)	(0.000)	(-)	(0.072)	(0.172)	(0.000)	(-)	(0.077)	(0.184)
	SK(har)	**4.000**	0.960	0.994	0.984	3.980	0.980	0.997	0.994	3.990	0.990	0.999	0.997
		(0.201)	(-)	(0.019)	(0.044)	(0.141)	(-)	(0.018)	(0.041)	(0.100)	(-)	(0.013)	(0.029)
	SK(gap)	2.000	0.000	0.748	0.494	2.000	0.000	0.748	0.494	3.030	0.510	0.878	0.754
		(0.000)	(-)	(0.000)	(0.000)	(0.000)	(-)	(0.000)	(0.000)	(1.000)	(-)	(0.126)	(0.253)

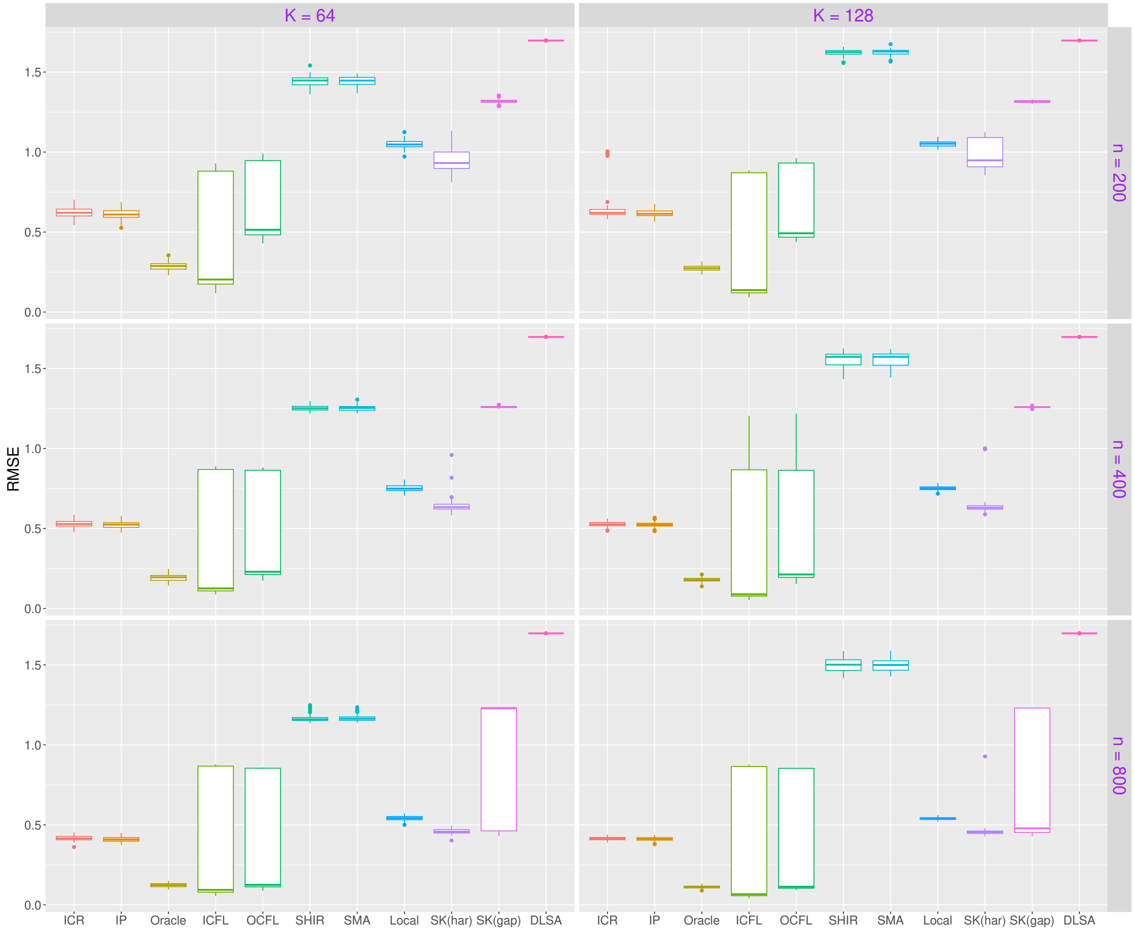

Figure 5: Boxplots of RMSE in Example 2.

Table 6: The variable selection accuracy: mean (sd) based on 100 replicates in Example 3.

		$K = 16$			$K = 32$			$K = 64$		
	Method	TPR	FPR	MS	TPR	FPR	MS	TPR	FPR	MS
$\sigma = 1$	ICR	**1.000**	**0.000**	**16.000**	**1.000**	**0.000**	**16.000**	**1.000**	**0.000**	16.060
		(0.000)	(0.000)	(0.000)	(0.000)	(0.000)	(0.000)	(0.000)	(0.002)	(0.343)
	IP	**1.000**	**0.000**	**16.000**	**1.000**	**0.000**	**16.000**	**1.000**	**0.000**	**16.000**
		(0.000)	(0.000)	(0.000)	(0.000)	(0.000)	(0.000)	(0.000)	(0.000)	(0.000)
	ICFL	0.980	0.070	28.640	0.990	0.007	17.140	**1.000**	**0.000**	16.040
		(0.141)	(0.027)	(5.832)	(0.100)	(0.010)	(2.366)	(0.000)	(0.002)	(0.281)
	OCFL	0.980	0.156	44.300	0.991	0.042	23.580	**1.000**	0.003	16.580
		(0.141)	(0.046)	(9.344)	(0.088)	(0.024)	(4.942)	(0.000)	(0.006)	(1.112)
	SHIR	**1.000**	0.019	129.920	**1.000**	0.022	258.030	**1.000**	0.015	513.410
		(0.000)	(0.016)	(2.347)	(0.000)	(0.015)	(1.374)	(0.000)	(0.014)	(1.248)
	SMA	**1.000**	0.001	128.410	**1.000**	0.002	256.190	**1.000**	0.003	512.240
		(0.000)	(0.003)	(2.257)	(0.000)	(0.005)	(0.419)	(0.000)	(0.005)	(0.495)
	Local	0.990	0.116	297.450	0.989	0.117	598.030	0.990	0.115	1184.390
		(0.008)	(0.022)	(32.006)	(0.007)	(0.015)	(43.364)	(0.004)	(0.011)	(62.616)
	SK(har)	0.994	0.382	198.580	0.998	0.571	300.420	**1.000**	0.802	383.890
		(0.033)	(0.183)	(74.091)	(0.018)	(0.201)	(95.568)	(0.000)	(0.142)	(90.112)
	SK(gap)	**1.000**	0.646	134.820	**1.000**	0.861	174.340	**1.000**	0.982	196.700
		(0.000)	(0.104)	(19.080)	(0.000)	(0.062)	(11.353)	(0.000)	(0.019)	(3.416)
	DLSA	0.445	0.431	43.250	0.424	0.402	40.370	0.395	0.372	37.420
		(0.141)	(0.104)	(10.011)	(0.195)	(0.109)	(10.977)	(0.161)	(0.102)	(9.995)
	WONDER	0.124	0.113	11.390	0.073	0.066	6.640	0.026	0.029	2.880
		(0.251)	(0.231)	(23.132)	(0.202)	(0.186)	(18.659)	(0.127)	(0.125)	(12.483)
$\sigma = 2$	ICR	0.996	**0.000**	**16.190**	**1.000**	**0.000**	**16.000**	**1.000**	**0.000**	16.060
		(0.021)	(0.003)	(1.522)	(0.000)	(0.000)	(0.000)	(0.000)	(0.002)	(0.343)
	IP	0.996	**0.000**	16.200	**1.000**	**0.000**	**16.000**	**1.000**	**0.000**	**16.000**
		(0.021)	(0.001)	(1.435)	(0.000)	(0.000)	(0.000)	(0.000)	(0.000)	(0.000)
	ICFL	0.899	0.344	77.300	0.951	0.195	49.970	**1.000**	0.057	26.440
		(0.251)	(0.069)	(15.793)	(0.199)	(0.100)	(17.030)	(0.000)	(0.024)	(4.500)
	OCFL	0.823	0.480	101.120	0.858	0.333	73.870	0.990	0.166	46.300
		(0.254)	(0.100)	(21.438)	(0.294)	(0.116)	(23.646)	(0.068)	(0.072)	(13.353)
	SHIR	**1.000**	0.135	228.320	**1.000**	0.144	436.990	**1.000**	0.139	762.890
		(0.000)	(0.064)	(81.849)	(0.000)	(0.054)	(116.607)	(0.000)	(0.034)	(134.836)
	SMA	**1.000**	0.111	283.240	**1.000**	0.110	515.970	**1.000**	0.088	772.130
		(0.000)	(0.036)	(55.266)	(0.000)	(0.057)	(182.428)	(0.000)	(0.048)	(266.909)
	Local	0.816	0.099	250.050	0.818	0.100	504.280	0.819	0.099	1000.380
		(0.034)	(0.020)	(31.474)	(0.022)	(0.015)	(44.000)	(0.016)	(0.011)	(65.717)
	SK(har)	0.986	0.319	168.58	0.996	0.485	255.770	**1.000**	0.701	365.340
		(0.053)	(0.166)	(71.974)	(0.038)	(0.203)	(97.201)	(0.000)	(0.204)	(118.276)
	SK(gap)	**1.000**	0.555	118.370	**1.000**	0.809	165.630	**1.000**	0.967	193.840
		(0.000)	(0.150)	(26.845)	(0.000)	(0.084)	(15.860)	(0.000)	(0.026)	(4.745)
	DLSA	0.648	0.657	65.630	0.590	0.622	61.900	0.649	0.638	63.860
		(0.178)	(0.076)	(7.498)	(0.170)	(0.075)	(7.132)	(0.160)	(0.073)	(7.027)
	WONDER	0.141	0.137	13.690	0.088	0.093	9.250	0.028	0.033	3.290
		(0.249)	(0.233)	(23.212)	(0.211)	(0.212)	(21.085)	(0.112)	(0.110)	(10.931)

Table 7: The clustering accuracy: mean (sd) based on 100 replicates in Example 3.

		$K = 16$				$K = 32$				$K = 64$			
	Method	$\widehat{M}$	Per	RI	ARI	$\widehat{M}$	Per	RI	ARI	$\widehat{M}$	Per	RI	ARI
$\sigma = 1$	ICR	**2.000**	**1.000**	**1.000**	**1.000**	**2.000**	**1.000**	**1.000**	**1.000**	**2.000**	**1.000**	**1.000**	**1.000**
		(0.000)	(-)	(0.000)	(0.000)	(0.000)	(-)	(0.000)	(0.000)	(0.000)	(-)	(0.000)	(0.000)
	IP	**2.000**	**1.000**	**1.000**	**1.000**	**2.000**	**1.000**	**1.000**	**1.000**	**2.000**	**1.000**	**1.000**	**1.000**
		(0.000)	(-)	(0.000)	(0.000)	(0.000)	(-)	(0.000)	(0.000)	(0.000)	(-)	(0.000)	(0.000)
	ICFL	**2.000**	**1.000**	0.990	0.980	**2.000**	**1.000**	0.995	0.990	**2.000**	**1.000**	**1.000**	**1.000**
		(0.000)	(-)	(0.074)	(0.141)	(0.000)	(-)	(0.051)	(0.100)	(0.000)	(-)	(0.000)	(0.000)
	OCFL	**2.000**	**1.000**	0.990	0.980	**2.000**	**1.000**	0.995	0.990	**2.000**	**1.000**	**1.000**	**1.000**
		(0.000)	(-)	(0.074)	(0.141)	(0.000)	(-)	(0.051)	(0.100)	(0.000)	(-)	(0.000)	(0.000)
	SK(har)	4.900	0.000	0.752	0.483	5.340	0.000	0.739	0.468	4.790	0.000	0.744	0.484
		(1.418)	(-)	(0.085)	(0.182)	(1.821)	(-)	(0.088)	(0.182)	(1.274)	(-)	(0.065)	(0.132)
	SK(gap)	**2.000**	**1.000**	**1.000**	**1.000**	**2.000**	**1.000**	**1.000**	**1.000**	**2.000**	**1.000**	**1.000**	**1.000**
		(0.000)	(-)	(0.000)	(0.000)	(0.000)	(-)	(0.000)	(0.000)	(0.000)	(-)	(0.000)	(0.000)
$\sigma = 2$	ICR	2.020	0.980	**0.999**	**0.998**	**2.000**	**1.000**	**1.000**	**1.000**	**2.000**	**1.000**	**1.000**	**1.000**
		(0.141)	(-)	(0.008)	(0.017)	(0.000)	(-)	(0.000)	(0.000)	(0.000)	(-)	(0.000)	(0.000)
	IP	2.030	0.970	0.998	0.996	**2.000**	**1.000**	**1.000**	**1.000**	**2.000**	**1.000**	**1.000**	**1.000**
		(0.171)	(-)	(0.013)	(0.026)	(0.000)	(-)	(0.000)	(0.000)	(0.000)	(-)	(0.000)	(0.000)
	ICFL	**1.990**	**0.990**	0.926	0.860	1.970	0.970	0.964	0.930	**2.000**	**1.000**	**1.000**	**1.000**
		(0.100)	(-)	(0.183)	(0.349)	(0.171)	(-)	(0.132)	(0.256)	(0.000)	(-)	(0.000)	(0.000)
	OCFL	**1.990**	**0.990**	0.827	0.665	1.970	0.970	0.887	0.779	**2.000**	**1.000**	0.983	0.965
		(0.100)	(-)	(0.225)	(0.433)	(0.171)	(-)	(0.199)	(0.389)	(0.000)	(-)	(0.074)	(0.149)
	SK(har)	4.800	0.000	0.764	0.507	5.180	0.000	0.754	0.497	5.200	0.000	0.736	0.466
		(1.589)	(-)	(0.097)	(0.207)	(1.850)	(-)	(0.095)	(0.195)	(1.589)	(-)	(0.077)	(0.156)
	SK(gap)	2.020	0.980	0.996	0.993	2.010	0.990	0.998	0.997	**2.000**	**1.000**	0.999	0.999
		(0.141)	(-)	(0.019)	(0.039)	(0.100)	(-)	(0.013)	(0.026)	(0.000)	(-)	(0.004)	(0.009)

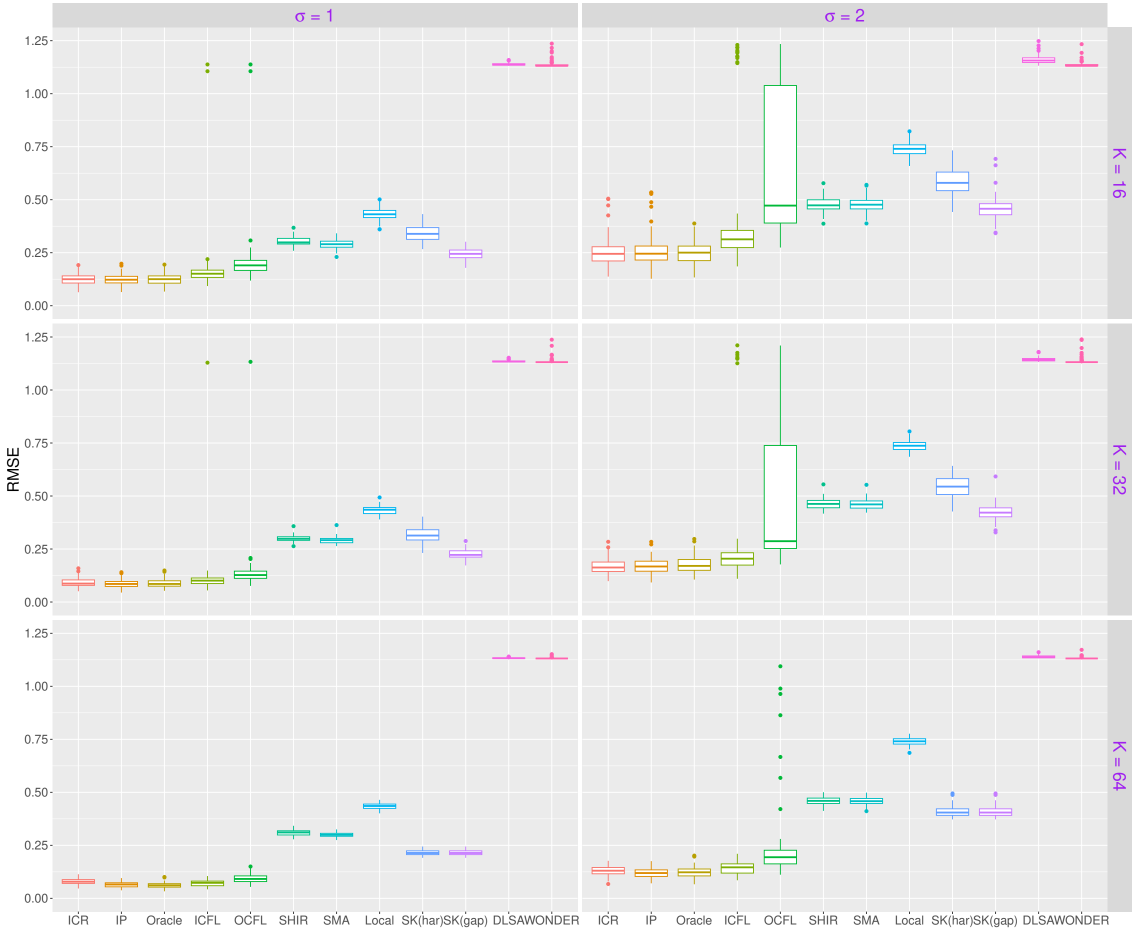

Figure 6: Boxplots of RMSE in Example 3.

Table 8: The variable selection accuracy: mean (sd) based on 100 replicates in Example 4.

		$n=100$			$n=200$			$n=400$		
	Method	TPR	FPR	MS	TPR	FPR	MS	TPR	FPR	MS
$\sigma=1$	ICR	**1.000**	**0.000**	32.040	**1.000**	**0.000**	**32.000**	**1.000**	**0.000**	**32.000**
		(0.000)	(0.001)	(0.400)	(0.000)	(0.000)	(0.000)	(0.000)	(0.000)	(0.000)
	IP	**1.000**	**0.000**	**32.000**	**1.000**	**0.000**	**32.000**	**1.000**	**0.000**	**32.000**
		(0.000)	(0.000)	(0.000)	(0.000)	(0.000)	(0.000)	(0.000)	(0.000)	(0.000)
	ICFL	0.915	0.012	33.250	0.885	**0.000**	28.440	0.900	**0.000**	28.880
		(0.189)	(0.057)	(22.316)	(0.211)	(0.002)	(6.891)	(0.201)	(0.002)	(6.522)
	OCFL	0.918	0.040	43.440	0.885	0.001	28.600	0.900	**0.000**	28.880
		(0.185)	(0.050)	(21.119)	(0.211)	(0.005)	(7.200)	(0.201)	(0.002)	(6.522)
	SHIR	**1.000**	0.077	531.640	**1.000**	0.081	524.450	**1.000**	0.072	523.670
		(0.000)	(0.029)	(31.566)	(0.000)	(0.030)	(17.815)	(0.000)	(0.030)	(17.975)
	SMA	**1.000**	0.060	539.570	**1.000**	0.078	524.870	**1.000**	0.073	525.040
		(0.000)	(0.026)	(38.983)	(0.000)	(0.030)	(19.013)	(0.000)	(0.028)	(21.384)
	Local	0.989	0.204	1709.120	**1.000**	0.199	1685.360	**1.000**	0.195	1658.000
		(0.005)	(0.012)	(70.651)	(0.000)	(0.011)	(63.342)	(0.000)	(0.011)	(64.948)
	SK(har)	0.999	0.932	375.080	**1.000**	0.933	375.160	**1.000**	0.937	376.680
		(0.013)	(0.137)	(50.832)	(0.000)	(0.098)	(36.120)	(0.000)	(0.036)	(13.237)
	SK(gap)	**1.000**	0.998	203.380	**1.000**	0.967	301.720	**1.000**	0.959	320.040
		(0.000)	(0.010)	(24.503)	(0.000)	(0.036)	(89.220)	(0.000)	(0.039)	(83.909)
	DLSA	0.359	0.305	30.950	0.203	0.069	7.930	0.168	0.025	3.660
		(0.167)	(0.116)	(11.400)	(0.123)	(0.067)	(6.753)	(0.076)	(0.035)	(3.514)
	WONDER	0.006	0.012	1.120	0.000	**0.000**	0.000	0.000	**0.000**	0.000
		(0.037)	(0.072)	(6.896)	(0.000)	(0.000)	(0.000)	(0.000)	(0.000)	(0.000)
$\sigma=2$	ICR	**1.000**	**0.000**	32.400	**1.000**	**0.000**	**32.000**	**1.000**	**0.000**	32.040
		(0.000)	(0.000)	(1.752)	(0.000)	(0.000)	(0.000)	(0.000)	(0.001)	(0.400)
	IP	**1.000**	**0.000**	**32.240**	**1.000**	**0.000**	**32.000**	**1.000**	**0.000**	**32.000**
		(0.000)	(0.000)	(1.372)	(0.000)	(0.000)	(0.000)	(0.000)	(0.000)	(0.000)
	ICFL	0.916	0.175	91.030	0.915	0.048	47.080	0.905	0.008	31.840
		(0.179)	(0.101)	(40.681)	(0.189)	(0.042)	(18.872)	(0.197)	(0.030)	(13.465)
	OCFL	0.915	0.340	150.420	0.915	0.065	53.200	0.905	0.009	32.120
		(0.163)	(0.119)	(49.251)	(0.189)	(0.049)	(21.846)	(0.197)	(0.031)	(13.709)
	SHIR	**1.000**	0.270	1542.320	**1.000**	0.229	1246.210	**1.000**	0.197	1084.500
		(0.000)	(0.080)	(486.815)	(0.000)	(0.076)	(367.035)	(0.000)	(0.068)	(350.924)
	SMA	**1.000**	0.263	1575.730	**1.000**	0.230	1246.960	**1.000**	0.188	1058.500
		(0.000)	(0.0840)	(469.041)	(0.000)	(0.076)	(391.310)	(0.000)	(0.068)	(374.399)
	Local	0.719	0.127	1116.360	0.944	0.176	1519.300	0.998	0.194	1650.780
		(0.030)	(0.013)	(85.809)	(0.014)	(0.012)	(75.027)	(0.002)	(0.011)	(64.969)
	SK(har)	0.908	0.285	140.730	0.994	0.868	351.280	0.999	0.928	373.640
		(0.106)	(0.410)	(161.999)	(0.033)	(0.223)	(83.034)	(0.013)	(0.101)	(37.494)
	SK(gap)	0.998	0.980	196.360	**1.000**	0.998	199.580	**1.000**	0.991	230.660
		(0.025)	(0.100)	(18.750)	(0.000)	(0.005)	(0.955)	(0.000)	(0.022)	(68.584)
	DLSA	0.614	0.608	60.830	0.368	0.228	23.920	0.248	0.044	6.070
		(0.191)	(0.086)	(8.705)	(0.159)	(0.109)	(10.723)	(0.132)	(0.048)	(5.127)
	WONDER	0.036	0.045	4.440	0.000	**0.000**	0.000	0.000	**0.000**	0.000
		(0.136)	(0.135)	(13.444)	(0.000)	(0.000)	(0.000)	(0.000)	(0.000)	(0.000)

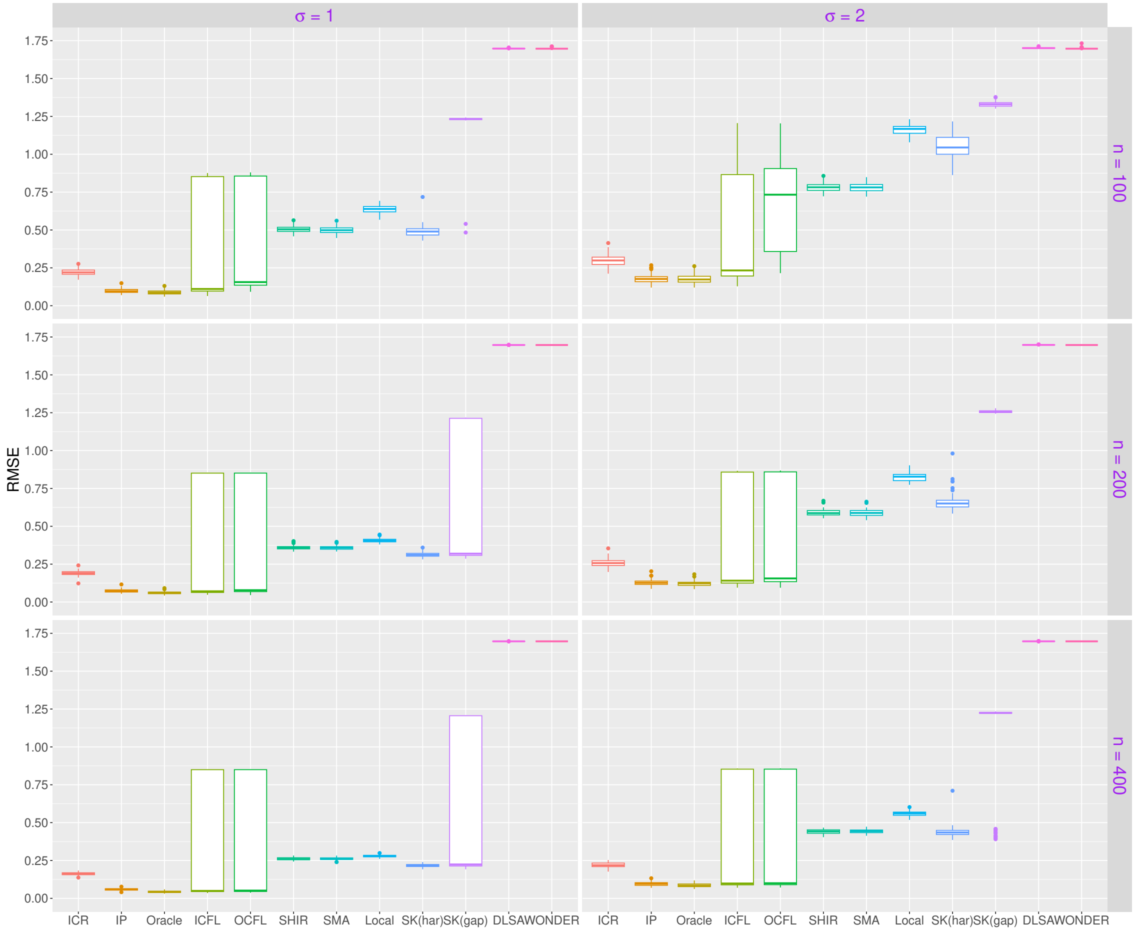

Figure 7: Boxplots of RMSE in Example 4.

Table 9: The clustering accuracy: mean (sd) based on 100 replicates in Example 4.

		$n = 100$				$n = 200$				$n = 400$			
	Method	$\widehat{M}$	Per	RI	ARI	$\widehat{M}$	Per	RI	ARI	$\widehat{M}$	Per	RI	ARI
$\sigma = 1$	ICR	**4.000**	**1.000**	**1.000**	**1.000**	**4.000**	**1.000**	**1.000**	**1.000**	**4.000**	**1.000**	**1.000**	**1.000**
		(0.000)	(-)	(0.000)	(0.000)	(0.000)	(-)	(0.000)	(0.000)	(0.000)	(-)	(0.000)	(0.000)
	IP	**4.000**	**1.000**	**1.000**	**1.000**	**4.000**	**1.000**	**1.000**	**1.000**	**4.000**	**1.000**	**1.000**	**1.000**
		(0.000)	(-)	(0.000)	(0.000)	(0.000)	(-)	(0.000)	(0.000)	(0.000)	(-)	(0.000)	(0.000)
	ICFL	3.930	0.930	0.956	0.894	**4.000**	**1.000**	0.943	0.863	**4.000**	**1.000**	0.944	0.865
		(0.256)	(-)	(0.067)	(0.160)	(0.000)	(-)	(0.075)	(0.181)	(0.000)	(-)	(0.075)	(0.181)
	OCFL	3.930	0.930	0.956	0.894	**4.000**	**1.000**	0.943	0.863	**4.000**	**1.000**	0.944	0.865
		(0.256)	(-)	(0.067)	(0.160)	(0.000)	(-)	(0.075)	(0.181)	(0.000)	(-)	(0.075)	(0.181)
	SK(har)	**4.000**	**1.000**	**1.000**	**1.000**	**4.000**	**1.000**	**1.000**	**1.000**	**4.000**	**1.000**	**1.000**	**1.000**
		(0.000)	(-)	(0.000)	(0.000)	(0.000)	(-)	(0.000)	(0.000)	(0.000)	(-)	(0.000)	(0.000)
	SK(gap)	2.040	0.020	0.751	0.498	3.140	0.570	0.891	0.780	3.350	0.670	0.917	0.833
		(0.281)	(-)	(0.036)	(0.072)	(0.995)	(-)	(0.126)	(0.255)	(0.936)	(-)	(0.119)	(0.240)
$\sigma = 2$	ICR	4.050	0.950	**1.000**	**0.999**	**4.000**	**1.000**	**1.000**	**1.000**	**4.000**	**1.000**	**1.000**	**1.000**
		(0.219)	(-)	(0.002)	(0.005)	(0.000)	(-)	(0.000)	(0.000)	(0.000)	(-)	(0.000)	(0.000)
	IP	**4.030**	**0.970**	**1.000**	**0.999**	**4.000**	**1.000**	**1.000**	**1.000**	**4.000**	**1.000**	**1.000**	**1.000**
		(0.171)	(-)	(0.001)	(0.004)	(0.000)	(-)	(0.000)	(0.000)	(0.000)	(-)	(0.000)	(0.000)
	ICFL	3.880	0.890	0.946	0.872	**4.000**	**1.000**	0.949	0.877	**4.000**	**1.000**	0.949	0.876
		(0.356)	(-)	(0.072)	(0.168)	(0.000)	(-)	(0.073)	(0.176)	(0.000)	(-)	(0.073)	(0.177)
	OCFL	3.880	0.890	0.930	0.832	**4.000**	**1.000**	0.949	0.877	**4.000**	**1.000**	0.949	0.876
		(0.356)	(-)	(0.073)	(0.173)	(0.000)	(-)	(0.073)	(0.176)	(0.000)	(-)	(0.073)	(0.177)
	SK(har)	4.150	0.860	0.977	0.935	**4.000**	**1.000**	**1.000**	**1.000**	**4.000**	**1.000**	**1.000**	**1.000**
		(0.386)	(-)	(0.021)	(0.058)	(0.000)	(-)	(0.000)	(0.000)	(0.000)	(-)	(0.000)	(0.000)
	SK(gap)	2.000	0.000	0.745	0.485	2.000	0.000	0.746	0.488	2.340	0.170	0.789	0.575
		(0.000)	(-)	(0.005)	(0.010)	(0.000)	(-)	(0.000)	(0.000)	(0.755)	(-)	(0.096)	(0.193)

Table 10: The variable selection accuracy: mean (sd) under 100 replicates in Example 5.

		$p = 200$			$p = 500$			$p = 800$		
	Method	TPR	FPR	MS	TPR	FPR	MS	TPR	FPR	MS
$U = 5$	ICR	**1.000**	**0.000**	32.560	**1.000**	**0.000**	32.420	**1.000**	**0.000**	32.160
		(0.000)	(0.000)	(2.051)	(0.000)	(0.000)	(2.128)	(0.000)	(0.000)	(1.126)
	IP	**1.000**	**0.000**	**32.400**	**1.000**	**0.000**	**32.240**	**1.000**	**0.000**	**32.000**
		(0.000)	(0.000)	(1.752)	(0.000)	(0.000)	(1.372)	(0.000)	(0.000)	(0.000)
	ICFL	0.934	0.184	170.840	0.964	0.249	520.680	0.934	0.250	822.080
		(0.161)	(0.098)	(79.131)	(0.126)	(0.089)	(177.677)	(0.170)	(0.086)	(275.921)
	OCFL	0.934	0.103	109.160	0.963	0.016	61.840	0.926	0.002	37.040
		(0.162)	(0.045)	(37.966)	(0.121)	(0.024)	(47.272)	(0.175)	(0.004)	(14.345)
	SHIR	**1.000**	0.065	332.450	**1.000**	0.039	339.050	**1.000**	0.026	340.210
		(0.000)	(0.025)	(4.719)	(0.000)	(0.013)	(6.475)	(0.000)	(0.008)	(6.217)
	SMA	**1.000**	0.064	332.210	**1.000**	0.032	335.700	**1.000**	0.020	335.470
		(0.000)	(0.027)	(5.149)	(0.000)	(0.010)	(5.098)	(0.000)	(0.005)	(4.270)
	Local	0.838	0.100	1038.480	0.769	0.048	1199.800	0.733	0.032	1263.060
		(0.035)	(0.010)	(88.302)	(0.042)	(0.006)	(132.189)	(0.036)	(0.004)	(119.201)
	SK(har)	0.969	0.432	373.770	0.944	0.193	429.890	0.948	0.127	468.470
		(0.082)	(0.305)	(242.129)	(0.104)	(0.194)	(397.009)	(0.096)	(0.145)	(503.149)
	SK(gap)	**1.000**	0.881	355.890	**1.000**	0.624	632.980	**1.000**	0.486	786.440
		(0.000)	(0.046)	(24.108)	(0.000)	(0.094)	(95.989)	(0.000)	(0.061)	(96.642)
	DLSA	0.128	0.008	2.510	0.355	0.373	186.350	0.591	0.589	471.300
		(0.018)	(0.017)	(3.274)	(0.141)	(0.067)	(33.167)	(0.189)	(0.060)	(48.197)
$U = 10$	ICR	**1.000**	**0.000**	32.560	**1.000**	**0.000**	**32.080**	**1.000**	**0.000**	**32.000**
		(0.000)	(0.000)	(2.346)	(0.000)	(0.000)	(0.800)	(0.000)	(0.000)	(0.000)
	IP	**1.000**	**0.000**	**32.320**	**1.000**	**0.000**	**32.080**	**1.000**	**0.000**	**32.000**
		(0.000)	(0.000)	(1.576)	(0.000)	(0.000)	(0.800)	(0.000)	(0.000)	(0.000)
	ICFL	0.959	0.098	106.240	0.946	0.117	260.600	0.964	0.131	446.760
		(0.133)	(0.101)	(79.170)	(0.154)	(0.072)	(145.067)	(0.144)	(0.070)	(223.048)
	OCFL	0.959	0.066	81.240	0.958	0.020	69.400	0.966	0.008	55.360
		(0.134)	(0.076)	(59.372)	(0.128)	(0.019)	(39.399)	(0.137)	(0.036)	(114.654)
	SHIR	**1.000**	0.074	335.320	**1.000**	0.046	342.410	**1.000**	0.022	337.390
		(0.000)	(0.024)	(9.045)	(0.000)	(0.015)	(7.429)	(0.000)	(0.008)	(6.334)
	SMA	**1.000**	0.070	334.280	**1.000**	0.045	342.740	**1.000**	0.020	335.550
		(0.000)	(0.022)	(7.721)	(0.000)	(0.015)	(8.864)	(0.000)	(0.006)	(4.659)
	Local	0.919	0.121	1223.780	0.883	0.061	1488.800	0.865	0.043	1639.920
		(0.028)	(0.009)	(75.231)	(0.031)	(0.005)	(97.978)	(0.037)	(0.005)	(160.497)
	SK(har)	0.983	0.480	402.570	0.973	0.331	702.860	0.976	0.225	765.640
		(0.053)	(0.313)	(242.951)	(0.072)	(0.203)	(413.880)	(0.068)	(0.163)	(531.936)
	SK(gap)	**1.000**	0.913	366.800	**1.000**	0.719	723.220	**1.000**	0.587	945.320
		(0.000)	(0.096)	(36.036)	(0.000)	(0.043)	(42.432)	(0.000)	(0.081)	(128.451)
	DLSA	0.130	**0.000**	1.090	0.133	0.033	17.240	0.300	0.293	234.810
		(0.030)	(0.002)	(0.379)	(0.035)	(0.039)	(19.086)	(0.153)	(0.075)	(59.824)

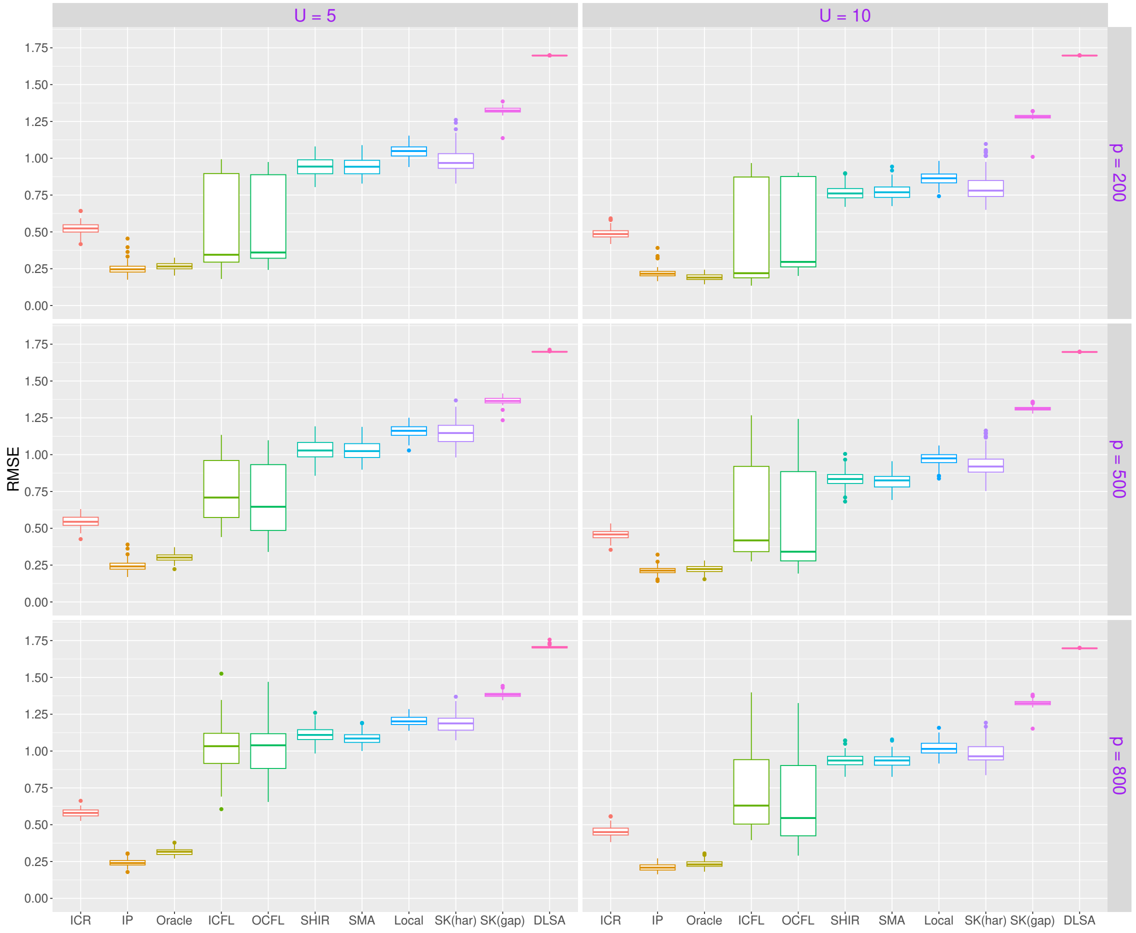

Figure 8: Boxplots of RMSE in Example 5.

Table 11: The clustering accuracy: mean (sd) based on 100 replicates in Example 5.

		$p = 200$				$p = 500$				$p = 800$			
	Method	$\widehat{M}$	Per	RI	ARI	$\widehat{M}$	Per	RI	ARI	$\widehat{M}$	Per	RI	ARI
$U = 5$	ICR	4.070	0.930	**0.999**	**0.998**	4.040	0.960	**1.000**	**0.999**	4.020	0.980	**1.000**	0.999
		(0.256)	(-)	(0.003)	(0.008)	(0.197)	(-)	(0.002)	(0.007)	(0.141)	(-)	(0.002)	(0.005)
	IP	4.050	0.950	**0.999**	**0.998**	4.030	0.970	**1.000**	**0.999**	**4.000**	**1.000**	**1.000**	**1.000**
		(0.219)	(-)	(0.003)	(0.007)	(0.171)	(-)	(0.002)	(0.006)	(0.000)	(-)	(0.000)	(0.000)
	ICFL	**4.000**	**1.000**	0.955	0.890	**4.000**	**1.000**	0.936	0.839	**4.000**	**1.000**	0.876	0.696
		(0.000)	(-)	(0.070)	(0.170)	(0.000)	(-)	(0.070)	(0.169)	(0.000)	(-)	(0.081)	(0.184)
	OCFL	**4.000**	**1.000**	0.955	0.890	**4.000**	**1.000**	0.935	0.836	**4.000**	**1.000**	0.876	0.695
		(0.000)	(-)	(0.070)	(0.170)	(0.000)	(-)	(0.070)	(0.169)	(0.000)	(-)	(0.081)	(0.185)
	SK(har)	4.180	0.830	0.964	0.901	4.250	0.780	0.939	0.833	4.340	0.750	0.936	0.820
		(0.796)	(-)	(0.038)	(0.103)	(1.029)	(-)	(0.048)	(0.127)	(1.165)	(-)	(0.042)	(0.116)
	SK(gap)	2.010	0.000	0.744	0.481	2.020	0.000	0.743	0.477	2.000	0.000	0.742	0.476
		(0.100)	(-)	(0.014)	(0.023)	(0.141)	(-)	(0.016)	(0.027)	(0.000)	(-)	(0.007)	(0.013)
$U = 10$	ICR	4.070	0.940	0.999	0.998	4.010	0.990	**1.000**	**1.000**	**4.000**	**1.000**	**1.000**	**1.000**
		(0.293)	(-)	(0.003)	(0.010)	(0.100)	(-)	(0.001)	(0.003)	(0.000)	(-)	(0.000)	(0.000)
	IP	4.040	0.960	**1.000**	**0.999**	4.010	0.990	**1.000**	**1.000**	**4.000**	**1.000**	**1.000**	**1.000**
		(0.197)	(-)	(0.002)	(0.007)	(0.100)	(-)	(0.001)	(0.003)	(0.000)	(-)	(0.000)	(0.000)
	ICFL	**4.000**	**1.000**	0.958	0.898	**4.000**	**1.000**	0.943	0.860	**4.000**	**1.000**	0.942	0.855
		(0.000)	(-)	(0.065)	(0.157)	(0.000)	(-)	(0.075)	(0.185)	(0.000)	(-)	(0.073)	(0.178)
	OCFL	**4.000**	**1.000**	0.958	0.898	**4.000**	**1.000**	0.943	0.860	**4.000**	**1.000**	0.942	0.854
		(0.000)	(-)	(0.065)	(0.157)	(0.000)	(-)	(0.075)	(0.185)	(0.000)	(-)	(0.073)	(0.178)
	SK(har)	4.050	0.900	0.981	0.950	4.130	0.890	0.974	0.926	4.100	0.910	0.967	0.907
		(0.575)	(-)	(0.033)	(0.081)	(0.800)	(-)	(0.032)	(0.090)	(0.541)	(-)	(0.033)	(0.090)
	SK(gap)	2.010	0.000	0.744	0.481	2.000	0.000	0.743	0.479	2.010	0.000	0.744	0.480
		(0.100)	(-)	(0.013)	(0.023)	(0.000)	(-)	(0.003)	(0.007)	(0.100)	(-)	(0.012)	(0.019)

Table 12: The Computational time: mean (sd) based on 100 replicates in Example 5. For methods (ICR, IP, SHIR and SMA), computation time refers to the average computation time for each tuning parameter based on a set of tuning parameters.

		Method	ICR	IP	ICFL	OCFL	SHIR	SMA
$U = 5$	$p = 200$	Time (seconds)	20.18	288.51	2.15	0.03	112.05	120.77
			(4.62)	(48.07)	(0.29)	(0.01)	(20.15)	(21.23)
	$p = 500$	Time (seconds)	77.72	1248.41	8.34	0.12	425.79	307.55
			(13.77)	(136.72)	(1.40)	(0.02)	(64.02)	(68.99)
	$p = 800$	Time (seconds)	151.32	2199.70	13.55	0.19	222.01	111.53
			(18.14)	(331.44)	(1.93)	(0.02)	(41.48)	(25.39)
$U = 10$	$p = 200$	Time (seconds)	13.80	293.61	6.31	0.08	130.55	180.83
			(3.80)	(36.41)	(1.24)	(0.01)	(13.36)	(23.52)
	$p = 500$	Time (seconds)	71.21	954.14	23.44	0.26	599.04	497.45
			(32.37)	(79.07)	(6.22)	(0.04)	(91.95)	(76.09)
	$p = 800$	Time (seconds)	91.09	3284.76	42.68	0.46	390.31	206.13
			(14.66)	(272.30)	(12.91)	(0.11)	(66.77)	(36.91)

Table 13: The variable selection accuracy: mean (sd) under 100 replicates in Example 6.

	$n_0 = 200$			$n_0 = 400$		
Method	TPR	FPR	MS	TPR	FPR	MS
ICR	**1.000**	**0.000**	**70.400**	**1.000**	**0.000**	**66.220**
	(0.000)	(0.000)	(11.274)	(0.000)	(0.000)	(3.823)
IP	0.999	**0.000**	70.560	**1.000**	**0.000**	**66.220**
	(0.009)	(0.000)	(11.654)	(0.000)	(0.000)	(3.823)
ICFL	0.891	0.054	87.900	0.867	0.013	64.380
	(0.164)	(0.086)	(50.671)	(0.175)	(0.061)	(35.674)
OCFL	0.895	0.046	83.580	0.875	0.009	62.520
	(0.149)	(0.079)	(46.077)	(0.171)	(0.060)	(34.506)
SHIR	0.963	**0.000**	826.750	0.990	0.002	942.920
	(0.058)	(0.002)	(114.240)	(0.039)	(0.005)	(86.582)
SMA	0.805	**0.000**	480.570	0.987	0.002	936.620
	(0.121)	(0.001)	(83.700)	(0.047)	(0.004)	(97.650)
Local	0.889	0.239	2794.920	0.961	0.285	3236.110
	(0.026)	(0.015)	(135.467)	(0.014)	(0.014)	(119.034)
SK(har)	0.987	0.870	470.700	**1.000**	0.934	561.980
	(0.070)	(0.262)	(171.349)	(0.000)	(0.155)	(120.517)
SK(gap)	0.975	0.960	193.320	0.936	0.870	177.460
	(0.129)	(0.197)	(39.317)	(0.177)	(0.338)	(66.105)
DLSA	0.704	**0.000**	7.740	0.777	**0.000**	8.550
	(0.159)	(0.000)	(1.750)	(0.149)	(0.000)	(1.641)

Table 14: The clustering accuracy: mean (sd) based on 100 replicates in Example 6.

	$n_0 = 200$				$n_0 = 400$			
Method	$\widehat{M}$	Per	RI	ARI	$\widehat{M}$	Per	RI	ARI
ICR	6.400	0.680	**0.993**	0.976	6.020	0.910	**0.977**	**0.992**
	(1.025)	(-)	(0.019)	(0.058)	(0.348)	(-)	(0.013)	(0.040)
IP	6.420	0.680	**0.993**	**0.977**	6.020	0.910	**0.977**	**0.992**
	(1.056)	(-)	(0.018)	(0.056)	(0.348)	(-)	(0.013)	(0.040)
ICFL	**6.000**	**1.000**	0.941	0.828	**6.000**	**1.000**	0.947	0.839
	(0.000)	(-)	(0.060)	(0.152)	(0.000)	(-)	(0.046)	(0.123)
OCFL	**6.000**	**1.000**	0.940	0.824	**6.000**	**1.000**	0.947	0.838
	(0.000)	(-)	(0.060)	(0.152)	(0.000)	(-)	(0.046)	(0.123)
SK(har)	5.240	0.420	0.940	0.837	5.970	0.510	0.977	0.932
	(1.334)	(-)	(0.073)	(0.178)	(0.989)	(-)	(0.043)	(0.115)
SK(gap)	2.010	0.000	0.652	0.320	2.020	0.000	0.651	0.321
	(0.100)	(-)	(0.021)	(0.024)	(0.141)	(-)	(0.035)	(0.038)

Table 15: The computational time: mean (sd) based on 100 replicates in Example 6. For methods (ICR, IP, SHIR and SMA), computation time refers to the average computation time for each tuning parameter based on a set of tuning parameters.

	Method	ICR	IP	ICFL	OCFL	SHIR	SMA
$n_0 = 200$	Time (seconds)	22.63	347.57	5.83	0.09	125.66	99.63
		(4.25)	(43.96)	(0.45)	(0.01)	(54.08)	(33.50)
$n_0 = 400$	Time (seconds)	19.65	657.10	20.88	0.26	84.15	59.21
		(3.44)	(84.94)	(3.11)	(0.03)	(23.17)	(11.94)

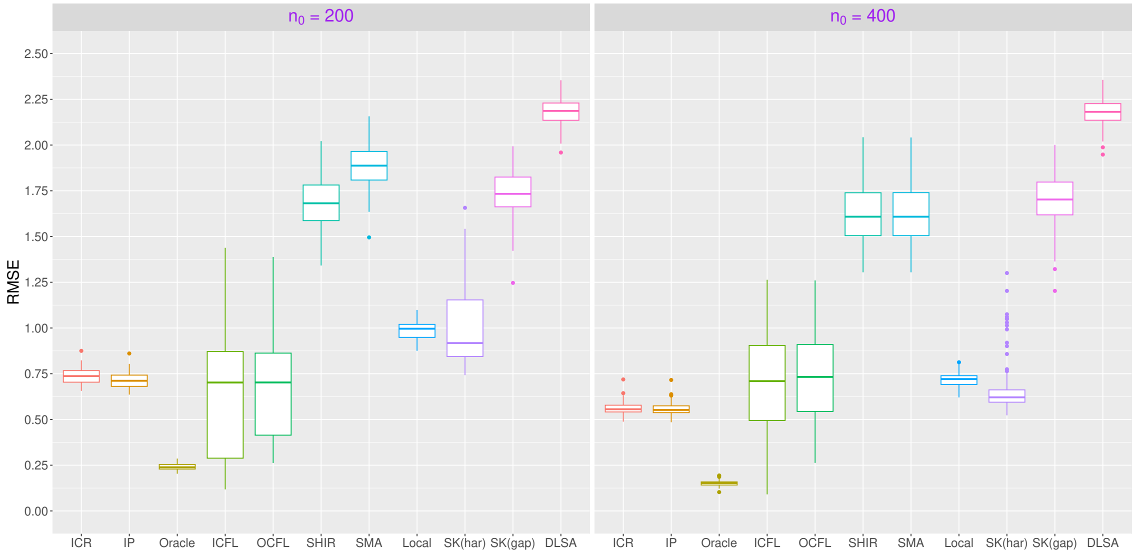

Figure 9: Boxplots of RMSE in Example 6.

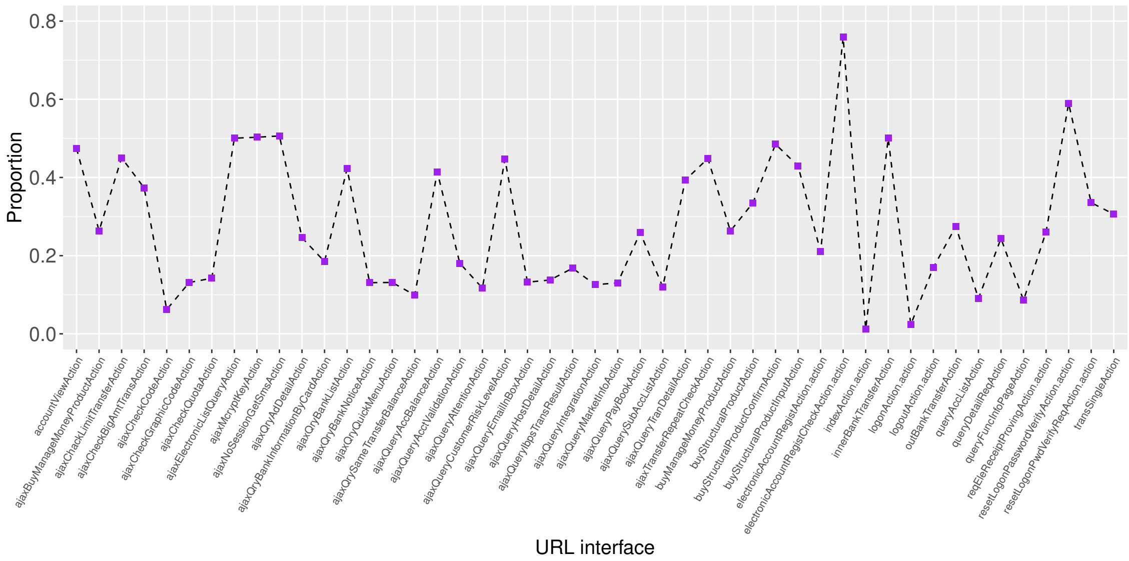

Figure 10: The abnormal proportions among the 47 URL interfaces in data analysis.

Table 16: The identified important variables and their estimates using the five integrative analysis methods in data analysis. For the proposed method, only estimates for the nontrivial clusters are shown.

Variable	$\text{ICFL}_{10}^{(1)}$	$\text{ICFL}_{10}^{(2)}$	$\text{ICFL}_{10}^{(3)}$	$\text{ICFL}_{10}^{(4)}$	$\text{ICFL}_{10}^{(5)}$	$\text{ICFL}_{10}^{(6)}$	$\text{ICFL}_{10}^{(7)}$	$\text{ICFL}_{10}^{(8)}$	$\text{ICFL}_{10}^{(9)}$
Intercept	1.401	-2.545	1.199	1.437	1.149	-0.970	0.926	0.868	1.406
Gnum	-0.181	0.142	-0.205	-0.239	–	0.288	-0.284	-0.139	0.606
Glen	-0.191	0.117	-0.231	-0.210	–	–	-0.316	–	-0.523
Pnum	–	-0.120	0.159	–	-0.207	0.903	0.323	–	-0.714
Plen	-0.380	-0.338	-0.334	–	–	0.417	–	2.115	0.149
$Gl0$	1.534	0.594	1.032	1.895	–	-0.272	1.372	2.800	-0.867
$Pl0$	1.031	1.026	1.730	–	0.394	0.641	2.282	0.431	–
$Pl1$	2.494	1.778	2.359	2.118	2.770	1.831	2.237	0.202	0.533
$Pl2$	2.369	1.712	2.103	2.730	2.840	0.797	0.376	–	0.363
$Pl3$	–	–	0.196	–	–	0.390	–	–	0.110
$Pl4$	0.106	0.101	0.101	–	0.228	0.276	–	–	2.439
$Pl5$	–	–	–	–	0.113	–	–	–	1.205
$Pl6$	–	–	–	–	–	–	–	–	0.622
$Pl7$	–	–	–	–	–	0.151	–	–	0.579
$Pl8$	–	–	–	–	–	–	–	–	-1.714
$Pl9$	–	–	–	–	0.118	–	–	–	0.288
$Pl10$	–	–	–	–	0.137	–	–	–	0.219
$Pl11$	–	–	–	–	–	–	–	–	0.168
$Pl12$	–	–	–	–	–	–	–	–	0.169
$Pl13$	–	–	–	–	–	–	–	–	0.139
$Pl14$	–	–	–	–	–	0.107	–	–	0.157
$Pl15$	–	–	–	–	–	0.110	–	–	0.175
$Pl17$	–	–	–	-0.131	–	–	–	–	–
$Pl18$	–	–	–	–	–	–	-0.104	–	–
$Pl19$	–	–	–	0.129	–	–	–	–	–
$GPw6$	–	–	–	–	-0.103	–	–	–	–
$GPw39$	–	0.118	–	–	–	–	–	–	–
$GPw68$	–	0.111	–	–	–	–	–	–	–
$GPw74$	–	-0.112	–	–	–	–	–	–	–

Table 17: One record of the initial request logs in data analysis.

URL interface	GET Parameter	POST Parameter
ajaxNoSessionGetSmsAction	s=captcha	_method=__construct&filter[]=phpinfo&<br>method=get&server[REQUEST_METHOD]=1
